# Social-ecological vulnerability of fishing communities to climate change: A U.S. West Coast case study

**DOI:** 10.1371/journal.pone.0272120

**Published:** 2022-08-17

**Authors:** Laura E. Koehn, Laura K. Nelson, Jameal F. Samhouri, Karma C. Norman, Michael G. Jacox, Alison C. Cullen, Jerome Fiechter, Mercedes Pozo Buil, Phillip S. Levin

**Affiliations:** 1 School of Environmental and Forest Sciences, University of Washington, Seattle, WA, United States of America; 2 Northwest Fisheries Science Center, National Marine Fisheries Service, National Oceanic and Atmospheric Administration, Seattle, WA, United States of America; 3 Southwest Fisheries Science Center, National Marine Fisheries Service, National Oceanic and Atmospheric Administration, Monterey, CA, United States of America; 4 Evans School of Public Policy and Governance, University of Washington, Seattle, WA, United States of America; 5 Ocean Sciences Department, University of California at Santa Cruz, Santa Cruz, CA, United States of America; 6 Institute of Marine Sciences, University of California Santa Cruz, Santa Cruz, CA, United States of America; 7 The Nature Conservancy in Washington, Seattle, WA, United States of America; University of New Haven, UNITED STATES

## Abstract

Climate change is already impacting coastal communities, and ongoing and future shifts in fisheries species productivity from climate change have implications for the livelihoods and cultures of coastal communities. Harvested marine species in the California Current Large Marine Ecosystem support U.S. West Coast communities economically, socially, and culturally. Ecological vulnerability assessments exist for individual species in the California Current but ecological and human vulnerability are linked and vulnerability is expected to vary by community. Here, we present automatable, reproducible methods for assessing the vulnerability of U.S. West Coast fishing dependent communities to climate change within a social-ecological vulnerability framework. We first assessed the ecological risk of marine resources, on which fishing communities rely, to 50 years of climate change projections. We then combined this with the adaptive capacity of fishing communities, based on social indicators, to assess the potential ability of communities to cope with future changes. Specific communities (particularly in Washington state) were determined to be at risk to climate change mainly due to economic reliance on at risk marine fisheries species, like salmon, hake, or sea urchins. But, due to higher social adaptive capacity, these communities were often not found to be the most vulnerable overall. Conversely, certain communities that were not the most at risk, ecologically and economically, ranked in the category of highly vulnerable communities due to low adaptive capacity based on social indicators (particularly in Southern California). Certain communities were both ecologically at risk due to catch composition and socially vulnerable (low adaptive capacity) leading to the highest tier of vulnerability. The integration of climatic, ecological, economic, and societal data reveals that factors underlying vulnerability are variable across fishing communities on the U.S West Coast, and suggests the need to develop a variety of well-aligned strategies to adapt to the ecological impacts of climate change.

## Introduction

Coastal communities are on the front line of climate change. Sea level rise is already threatening shoreline infrastructure [1], and extreme weather events are resulting in the destruction of the built environment along coastlines [2–4]. Climate change will also likely cause changes in fisheries resource availability [5] as a result of distributional shifts in fisheries species, declines in productivity, direct impacts on species due to warming, acidification, or declines in oxygen, and indirect loss through food-web disturbances [6–8]. Potential subsequent reductions in fisheries have significant implications for food security [9], culture [10] and livelihoods in coastal communities [11]. The impacts of such changes will vary among communities, households and individuals (e.g.,[12]), and may exacerbate existing inequalities within and among coastal communities [13]. The acute impacts of climate change on social-ecological systems have created an urgent need to strengthen the ability of coastal communities to adapt.

Along the West coast of the United States, marine fish and invertebrates are already experiencing substantial impacts from climate variability and change. Marine heat waves, such as the “warm blob”, have likely led to losses of commercial harvest in the California Current Ecosystem, including drastic declines in salmon abundance and landings, loss of revenue from closures in the Dungeness crab (*Metacarcinus magister*) fishery due to harmful algal blooms, and severe reduction in squid landings [14–19].

In total, fisheries in the U.S. portion of the California Current support over 220,000 jobs in communities in the states of Washington, Oregon, and California, and lead to total sales from commercial and recreational fishing in the region near $35 billion annually [20]. Community connection to fisheries resources is not just economic; the California Current Ecosystem supports community well-being by sustaining cultural values and practices, connections to nature, and social connections [21, 22]. These non-economic connections are particularly evident in tribal communities like Neah Bay, Washington, where residents catch and consume an average of 125 pounds per person of seafood annually and rely on subsistence catch to support culturally important household sharing networks and traditional knowledge [23]. Additionally, in non-tribal communities along the U.S. West Coast, core components of individual and community well-being reside in the marine environment and in human interactions with it [22, 24], and resident fishers derive non-monetary benefits from fishing practices that support expressions of identity and social capital [25].

Because communities rely upon fisheries economically and socially, the ecological impacts of climate change on fisheries resources are likely to affect livelihoods and well-being in the region. One way to assess these impacts is by describing vulnerability—the degree to which a system is likely to experience harm due to exposure to a hazard [26]. Following the Intergovernmental Panel on Climate Change (IPCC) [27], vulnerability to climate change can be conceptualized as a combination of exposure to climate change, the degree to which a system is affected by climate change (i.e., sensitivity), and the capacity to adapt to that change. In the context of environmental management, vulnerability assessments are useful for building an understanding of patterns of vulnerability (e.g., [28]), improving adaptation planning (e.g.,[29]), and revealing patterns of inequity [30].

Ecological approaches to vulnerability assessment in fisheries systems typically focus on target species [31]. For instance, Crozier and colleagues [32] evaluated the vulnerability of Pacific salmon to climate change by assessing the magnitude of the projected change in conditions resulting from climate shifts (exposure), the sensitivity of salmon to such changes, and the ability of salmon to modify phenotypes to cope with new climate conditions (adaptive capacity). In contrast, approaches to vulnerability assessment rooted in social-ecological systems theory (e.g., [33]), recognize that ecological and human vulnerability are linked [26] and thus integrate biophysical and social exposure, sensitivity and adaptive capacity [34–39]. Cinner and colleagues [35], for example, combined ecological exposure and sensitivity with social sensitivity and adaptive capacity to assess social-ecological vulnerability in 12 Kenyan coastal communities. Fully-integrated social-ecological vulnerability assessments developed at scales that align geographically with existing governance systems allow for more frictionless uptake and implementation of policies intended to reduce coastal community vulnerability (e.g., national level: [40, 41]; regional level: [42, 43]). Because of the fast-changing ecological and social contexts of these assessments, those which can be easily updated with new data and that are scalable are less likely to become quickly outdated (see, for example, [44, 45] and references therein).

Here, we develop and deploy an assessment that is grounded in both ecological and social-ecological vulnerability approaches. Our goal is to identify communities reliant on the U.S. California Current Ecosystem that are likely to become most imperiled by the impacts of climate change on fisheries. We first assess the ecological risk of the California Current major fisheries species to climate change into the medium-term future (50 years). We next estimate the degree to which fishing communities are at risk from climate-impacted fisheries species. Finally, we integrate the climate risk faced by California Current fishing communities with the adaptive capacity of these communities to assess the overall vulnerability of these coastal populations. Using readily available public data, our overall aim is to produce an assessment that is easily reproducible, automatable, and scalable and in this case aligned with the scale of the federal fisheries governing body, the Pacific Fisheries Management Council (PFMC). In doing so, we strive to provide needed information that can guide management interventions which inform more equitable climate adaptation.

## Methods

### Overview

**Fig 1:**
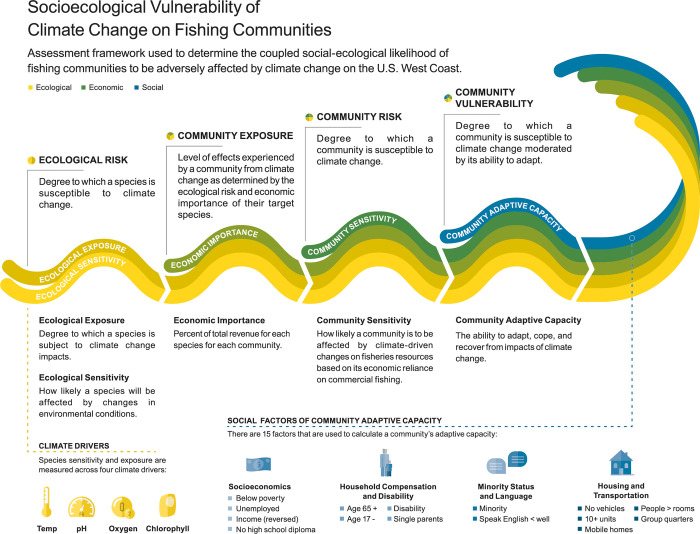
Vulnerability assessment framework. Framework used to determine the coupled social-ecological vulnerability of fishing communities to climate change on the U.S. West Coast. The initial components of the framework are the ecological sensitivity and exposure to climate change of marine resources that fishing communities depend on. Ecological sensitivity and exposure are determined for four climate change variables–temperature, pH, oxygen, and chlorophyll–and are combined to determine ecological risk. Community exposure is derived by weighting ecological risk of species by the economic importance to each community. When community exposure is combined with community sensitivity, this forms community risk to climate change. When community risk is combined with adaptive capacity, which is made up of 15 social indicators, this produces overall community vulnerability which is made up of ecological (yellow), economic (green), and social indicators (blue). Design by SJ Bowden.

We adapted the vulnerability assessment framework outlined by Marshall and colleagues [46] and Thiault and colleagues [47] to investigate the vulnerability of fishing communities (Fig 1). We first determine ecological risk, a combination of the ecological exposure and ecological sensitivity of target species to changing climate conditions (Table 1, Fig 1). Ecological risk directly informs community exposure, such that ecological risk of each target species is weighted by economic importance of those species for each community (Table 1). Community sensitivity is determined by the economic reliance of communities on the fishing industry, which when combined with community exposure, gives community risk to climate change (Table 1). We then consider the adaptive capacity of fishing communities, or the ability to adapt, absorb, and recover from climate change impacts, which is influenced by demographic and social factors [48]. Combining community risk with community adaptive capacity generates the overall extent that communities are vulnerable to climate change, or “community vulnerability” (Table 1). We calculate ecological risk for approximately 50 years into the future so that community vulnerability reflects medium-term future conditions. This time-frame is similar to that considered by the PFMC Climate and Community Initiative [49, 50], and is 1) a period where climate models and emissions are more certain (compared to further out, see [51]); 2) long-enough in the future where climate trends have emerged; and 3), near-term enough to still be management relevant. We focus our analysis on U.S. fishing communities in the California Current Ecosystem and define a fishing community as a geographic location that is a specified census designated place, with at least some level of commercial fishing activity associated with commercial fisheries along the continental U.S. West Coast (as defined by the National Oceanic and Atmospheric Administration and California Current Integrated Ecosystem Assessment, IEA [52]).

**Table 1:** Terms and definitions used in the vulnerability framework for coupled social-ecological systems.

Term & Variable used in equations	Definition
Ecological Exposure $\bar{e_{i,m}}$	Degree to which a species is subject to (exposed to) changing environmental conditions due to climate change. Calculated as the expected change in environmental conditions a species will face in their range (overlap between the historic range and future range of climate values a species experiences in its spatial range where greater overlap = lower exposure). Species range data comes from Aquamaps [53]. Average exposure for each species (*i*) for each climate model (*m*) across four climate variables: temperature, pH, chlorophyll, and oxygen (Eq 1)
Ecological Sensitivity $\bar{s_{i,m}}$	Conditions determining how likely a species will tolerate future changes in environmental conditions. Here, use a proxy calculated as present-day breadth of environmental conditions a species experiences in its range (where greater breadth implies greater tolerance). Average sensitivity for each species (*i*) for each climate model (*m*) across four climate variables: temperature, pH, chlorophyll, and oxygen (Eq 1)
Ecological risk *θ*_*i*,*m*_	Degree to which a species is susceptible to climate change. Combination of ecological exposure and ecological sensitivity to climate change (calculated as the Euclidean distance between sensitivity and exposure). Calculated for each species (*i*) and climate model (*m*) (Eq 1)
Community Exposure *E*_*c*_	Degree to which a community is subject to impacts of climate change based on the species targeted by the community. Ecological risk for each species in the community’s top 90% of landings, weighted by the percent revenue of each species for that community (*c*) (Eq 2) (similar to methods in [37]).
Community Sensitivity *S*_*c*_	Conditions determining how likely a community will be impacted by climate change. Calculated as community economic reliance on commercial fishing (from IEA [52] and see [54]). Calculated for each community (*c*).
Community Risk *R*_*c*_	Likelihood a community will be adversely affected by a climate change. Combination of community exposure and community sensitivity calculated for each community (*c*) (Eq 3).
Community Adaptive Capacity *A*_*c*_	Ability to adapt, absorb, and recover from climate change. Demographic and social indicators are known to directly impact a community’s adaptability [48]. Made up of a combination of social indicators from CDC [55]) for each community (*c*) where highest adaptive capacity = 0 and lowest = 1, so that for all axes—adaptive capacity, exposure, sensitivity—higher values equate to higher vulnerability.
Community vulnerability *V*_*c*_	Cumulative measure of potential climate change effects based on community exposure, community sensitivity, and adaptive capacity calculated for each community (*c*); calculated as the Euclidean distance between community sensitivity, exposure, and adaptive capacity (where higher values of each are associated with higher vulnerability) (Eq 4)

### Ecological

As defined above, ecological risk is the extent to which fishery target species are predicted to be affected by climate impacts. For ecological risk, we adapted the approach of Samhouri et al [56], where ecological risk is a combination of the expected change in environmental conditions a species will face in their range (ecological exposure) and the present-day breadth of environmental conditions a species experiences in its range (ecological sensitivity, where greater breadth implies greater tolerance). To estimate ecological risk, we compiled data and model output on commercial species ranges/distributions and environmental/climate conditions in those ranges for species that are the top landed species for communities. These quantitative methods are automatable through readily available data and scalable depending on species and geographic range.

#### Ecological data

To determine the commercial fishery species that communities are most dependent on, we relied upon landings receipts derived from the Pacific Fisheries Information Network (PacFIN; http://pacfin.psmfc.org/) database for the years 2009–2018 (10-year range to capture potential variability from year to year). Fish ticket data for 2009–2016 and 2017–2018 were downloaded in April 2017 and June 2019, respectively (the final quarter of 2016 was not complete at the time of the initial data query and not included in analysis, specifically 9/9/2016 through 12/31/2016). To assess landings, we identified fishing ports recognized by the Pacific Fisheries Management Council and state fish and wildlife agencies. Data on species landings are provided by PacFIN; however, PacFIN data aggregate multiple ports to higher level PacFIN “port groups”. As a consequence, we assumed landings data for each individual port are the same as the PacFIN port group that the individual port is a member of (see https://pacfin.psmfc.org/pacfin_pub/codes.php for all port groupings). We excluded ports where a high percent of landings data were confidential (see below). Because the PacFIN database is updated as information is released from California, Oregon, and Washington Departments of Fish and Wildlife to Pacific States Marine Fisheries Commission, it is possible that the top species by landings and/or assignments of landings to ports may change over time (even potentially becoming not confidential). The value of the method and framework introduced in this paper is that the analysis can be updated in parallel as data is updated.

For each port, we calculated the total landed weight over the 10-year span, found the percent of total landed weight each species comprised, and only considered the species that contributed to the top 90% of landed weight for the remainder of our analyses. We focused on the top 90% of landed weight, not revenue, to focus on the majority of what is caught in the community (versus a rare but highly valued species caught). Communities were removed if >20% of landings were listed as “confidential” (species landed by <3 vessels) as the majority of data either had <20% or more than 20% (break in the data) and then less then 75% of the landings were accounted for if more then 20% was confidential. Catch was removed for landings listed as “other” or “miscellaneous” and that made up less than 5% of catch. We determined species level for “unidentified” catches of taxonomic families (unidentified urchins, unidentified hagfish, etc.) based on information from state fish and wildlife agencies (see S1 Table for which species make up each group). Additional specifics on the preparation of landings data for analysis are provided in the S1 Appendix.

Our approach to ecological risk required historic and future climate data within species distributions. We chose four climate variables that may impact species and are major climate drivers in the California Current System [57]; temperature, pH, oxygen, and chlorophyll (see S1 Appendix for justification for each variable). We used multiple variables as recent studies have indicated that using temperature alone to specifically predict range shifts can underestimate or hide vulnerability [58] and these four variables have been used in other assessments [28, 32, 59, 60].

Species specific distributions for all species in the top 90% of landings were collated from Aquamaps [53] and we rasterized species range data using methods presented in O’Hara et al [61]. Final data include species distributions for the U.S. West Coast area of 180-100W, 20-60N, that are constrained further to the ocean model domain for climate variables (see below). We developed extent of occurrence [62] for each species based on probability of occurrence of ≥ 0.4 across 50 km by 50 km grid cells. We only included areas with species probability of occurrence ≥ 0.4 because areas of low probability of occurrence may not actually be within a species “core range” or relative suitable habitat and may only represent vagrant or extralimital occurrences. Other studies have suggested a cut-off of 0.6 for relative habitat suitability (specifically [63], but this study focused on marine mammals). A threshold of 0.4 is also often used or tested (for example [56, 64, 65]) and is more precautionary; some species suitable habitat ranges may be overestimated, but gives more confidence that we have captured the entire suitable habitat of a species.

A few species within the top 90% of landings by port had missing distribution data or distributions primarily outside of our range. These species were either removed from analysis or information from a closely related species was used depending on how prominent the species were in the landings data (see S1 Appendix). Of the 72 species in the top 90% of landings (for ports with <20% confidential and including species that make up unspecified species groups), we were able to estimate climate risk for 68 of them, and use a surrogate species for 1, for a total of 69 species (S1 Table). For each species, the metric used for the climate experienced by the species, for each climate variable (pH, temperature, oxygen, and chlorophyll), depended on the species primary habitat (benthic or pelagic); e.g. bottom temperature was used for benthic species and surface temperature for pelagic (see S1 Appendix on how primary habitat was determined and metrics used for bottom versus surface, and see S1 Table for info for each species).

We used a California Current configuration of the Regional Ocean Modeling System (ROMS) coupled with a biogeochemical model (NEMUCSC) to simulate historical and future climate conditions for temperature, pH, chlorophyll, or oxygen within species geographic ranges [66, 67]. The ROMS-NEMUCSC model domain spans 30–48˚N and from the coast to 134˚W with a horizontal resolution of 0.1 degrees (~10 km) and 42 terrain-following vertical levels. The coupled physical-biogeochemical model was forced by output from three global earth system models (ESMs) via dynamical regional downscaling, using a “time-varying delta” method for 1980–2100 under the CMIP5 high emissions scenario (RCP 8.5). The three global ESMs were GFDL-ESM2M [68, 69]), HadGEM2-ES [70]), and IPSL-CM5A-MR [71]. These three models were chosen because they capture the CMIP5 range of potential future physical and biogeochemical change in the California Current Ecosystem. Hereafter, we refer to the different downscaled climate projections as GFDL, IPSL, and HAD.

Calculations for ecological exposure and sensitivity were run with output from each of the three ESMs downscaled climate projections. “Historical” climate experienced by species in their ranges was calculated for the reference period 1980–2010, while future conditions were calculated for 2030–2060 (using an approximately 30 year time period to capture decadal variability in climate change). To exclude numerical artifacts near the model open boundaries, we only extracted data from 31–47˚N and from the coast to 133˚W, omitting ~100 km next to the open boundaries. While some of the species ranges extend beyond the model boundaries, we focus on the California Current domain that is proximate to the U.S. West Coast communities of interest and for which the higher resolution downscaled climate projections can resolve important finer-scale processes associated with coastal upwelling dynamics.

#### Ecological sensitivity and exposure

Ecological exposure of a species to a climate variable is the expected change (future-historical) in that climate variable within the species’ California Current range. Here, exposure is defined by the overlap between the historic and future distributions of climate conditions a species experiences. Specifically, for each species, ESM, and environmental variable (temperature, pH, etc.), we found the percent of the distribution of future values that falls in the 5^th^-95^th^ percentile range of the historical distribution, and then subtracted it from 100%. Greater overlap between historic and future climate experienced implies lower exposure to climate change, while lower overlap suggests higher exposure to shifts in climate conditions. This method varies from Samhouri et al. [56] where exposure was calculated as the change in the mean climate experienced between historic and future. We used the percent of future values that fall within the historical distribution to capture both magnitude of change in the mean as well as variability around the mean.

Ecological sensitivity was defined as the inverse of the current breadth of climate conditions experienced by a species within its current spatial range. Following Dickinson et al. [72], the climatic breadth is the percentile range (5^th^-95^th^) experienced by each species in its historical spatial distribution (calculated as the 95^th^ percentile value minus the 5^th^ percentile value and inversed). Species with greater climatic breadth are assumed to be less sensitive.

Ecological sensitivity and exposure for each species were then determined for each climate variable within each of the three downscaled climate projections. We log10-transformed exposure and sensitivity for each of the four climate variables separately across species to minimize influence of extreme values and then scaled to range between 0 and 1 for ease of interpretation and standardization (closer to 1 = higher sensitivity or exposure). We then combined estimates of ecological sensitivity or exposure for each species by averaging across the four climate variables, giving species average ecological exposure ($\bar{e_{i,m}}$) and species average ecological sensitivity ($\bar{s_{i,m}}$), for each species *i* and model *m* for all climate variables. This approach assumes that ecological exposure and sensitivity to each climate variable for each species represent equal contribution to overall ecological exposure and sensitivity to climate change.

#### Ecological risk

Estimates for average species’ ecological sensitivity and exposure were then combined using Euclidean distance to find ecological risk (cf. [73]):

$$\theta_{i,m}=\sqrt{{\left(\bar{e_{i,m}}-min(\bar{e_{i,m}}\right)}^{2}+{\left(\bar{s_{i,m}}-\min\left(\bar{s_{i,m}}\right)\right)}^{2}}$$ Eq 1

where *θ*_*i*,*m*_ is ecological risk for species *i* and model *m*, and ranges from zero to larger values. This calculation means that risk increases with distance from the origin in exposure-sensitivity space and that both exposure and sensitivity have equal contribution to risk. We averaged across the three climate models and use these average values of ecological risk moving forward (but see S2 Table for ecological risk calculated from each model). Ecological risk is moderately correlated across models when species are ranked (Spearman rank correlation; R = 0.46–0.7, S1 Fig). We also calculated ecological risk for additional species important to West Coast Indigenous communities (see S3 Table). Olive rockfish was included in calculations though was only caught by communities that were removed due to >20% confidential catch. Removal of this species does not change risk values for the other species.

### Community risk

#### Community exposure

Community exposure is the degree to which communities are subject to the impact of climate change. Therefore, the exposure of a community is directly related to ecological risk of the species they depend on. To estimate community exposure, we weighted ecological risk for each species by the percent of total revenue for each species for each community, and then summed across the weighted values. Since we have removed confidential catch and species with missing information, the percent of revenue is not out of the total revenue for that community, but the percent revenue of the total revenue for species in the top 90% of landings for that community left after removing missing and confidential data.


$$E_{c}=\sum_{i}\bar{\theta_{i}}p_{i,c}$$ Eq 2

Where *E*_*c*_ is exposure for each community, $\bar{\theta_{i}}$ is average ecological risk per species across the three climate models, and *p*_*i*,*c*_ is the proportion of revenue of species *i* in community *c*. We then found the percentile ranking of the community exposure score across communities to scale exposure for each community between 0 and 1. Note that we weighted by percent revenue but weighting by percent landings (for species) produced similar rankings across communities (correlation coefficient = 0.94, see S2 Fig). Community exposure is highly correlated across the three climate models that we averaged across to find average ecological risk (see S2 Fig, all correlations).

#### Community sensitivity

Community sensitivity reflects the conditions determining how likely a community will be affected by climate change. Since we are primarily concerned with changes in fisheries, we defined the sensitivity of a community to climate change as its economic reliance on the commercial fishing industry. For each community, we used an index of commercial fishery reliance that includes landings, revenue, permits and processing from the California Current Integrated Ecosystem Assessment [52] for the year 2017 (most recent year available). To scale commercial reliance between 0 and 1, we found the percentile rank for each community across reliance for all communities (which we denote *S*_*c*_), where multiple communities had the lowest reliance and have sensitivity scores of 0.

#### Community risk

We combined community exposure and community sensitivity, using Euclidean distance to get community risk (*R*_*c*_) for each community (where minimum exposure and sensitivity are both 0).


$$R_{c}=\sqrt{{\left(E_{c}\right)}^{2}+{\left(S_{c}\right)}^{2}}$$ Eq 3

In calculations of community sensitivity, exposure, and risk, we used the species in the top 90% of landings (by weight) in current and recent years. We recognize that this species composition of landings may not be the same for future landings; however, absent other information it was the best available information.

### Community adaptive capacity

We next estimated the adaptive capacity of each fishing community. Here, adaptive capacity refers to the ability of a community to cope with a hazard or pressure such as climate change [74]. We based our analysis of adaptive capacity on Flanagan and colleagues’ [48] work, following approaches similar to those used by Davies et al. [30] and Messager et al. [75] to assess the vulnerability of communities to wildfire and flooding, respectively.

Like Flanagan et al. [48], we used an index of adaptive capacity based on metrics (n = 15) nested within four themes: 1) socioeconomic—persons below poverty, unemployed civilians, per capita income, persons with no high school diploma; 2) household composition/disability—persons age 65+, persons age 17 and less, noninstitutionalized population with a disability, number of single parent households; 3) minority status/language—persons of minority, persons older than 5 that don’t speak English well; and 4) housing/transportation—housing structures with 10+ units, estimates of mobile homes, households with more people than rooms (crowding), households with no vehicles, and persons in institutionalized group quarters. These data are compiled by the Centers for Disease Control and Prevention (CDC) from the American Community Survey and are presented as an index (called the CDC Social Vulnerability index) averaged over years 2014–2018 [55]. Hereafter, we refer to this index as the “CDC Index”.

The metrics used in the CDC Index are estimated at a census tract level; therefore, for each port community, we found the census tracts that intersect with each community using the R packages acs [76], sf [77], tidycensus [78], and tigris [79]. We first found the geographic boundary of each community by state, and then found the census tracts that intersected at least partially with each community boundary. We matched data from the CDC Index to the census tracts in each community and averaged values across census tracts to find an overall score for each community for each of the 15 metrics for 2018 CDC data. We percentile ranked each of the 15 metrics across communities so that each metric is on the same scale (0 to 1), then summed across metrics for each community, and percentile ranked again across communities so that adaptive capacity is on the same scale as community exposure and sensitivity (cf [55]). This gives an index value between 0 and 1.

Increasing values of the CDC Index indicate worse conditions (e.g., more unemployment, more households without vehicles, etc), so for the per capita income metric, we reversed the percentile ranking (1 minus original ranking) so that higher values equal worse conditions in line with the other metrics (cf. [55]). As larger values equate to worse conditions, we present adaptive capacity as the CDC Index but where 0 (smallest value of the CDC Index) represents the greatest capacity to adapt and 1 (highest value of CDC Index) reflects the lowest adaptive capacity. From this, all axes of vulnerability—sensitivity, exposure, and adaptive capacity–are such that greater values equal greater vulnerability.

NOAA also calculates an annual social index using similar data [52], using methods described in [37, 54]. The adaptive capacity generated using the NOAA index is highly correlated to that generated using the CDC index (R = 0.94, see S1 Appendix for more information on the NOAA index and S3 Fig for correlation). Because the CDC Index encompasses more communities and the two indices are highly correlated, we show the results based solely on the CDC Index.

### Overall community vulnerability

We estimated community vulnerability as the Euclidean distance of community risk and adaptive capacity as:

$$V_{c}=\sqrt{{\left(R_{c}\right)}^{2}+{\left({AC}_{c}\right)}^{2}}$$ Eq 4

where *V*_*c*_ is community vulnerability by community (*c*), *R*_*c*_ is community risk, and *AC*_*c*_ is adaptive capacity.

Note that across all methods of calculating ecological risk, community risk, and community vulnerability, scaling values at various times in relation to averaging or calculating Euclidean distance only minimally changes the results for species risk or final community vulnerability. For example, re-scaling ecological sensitivity and exposure before calculating ecological risk using Euclidean distance or re-scaling ecological risk by climate model before averaging, does not qualitatively change the final result—communities with the highest vulnerability remain the same, and rank order of communities with lower vulnerability shifts only slightly. As presented throughout the methods, estimates of each component (community sensitivity, exposure, and adaptive capacity) were correlated across data and models showing that our results are robust to certain sources of uncertainty (see supporting information figures). We do not present further analysis of uncertainty for community vulnerability because, though we present results across multiple sources of uncertainty for species risk and associated community exposure (such as climate model used, weighting by revenue vs. landings, see supporting information figures and tables), we do not have the equivalent ability to represent uncertainty for the other components (only one measure of community sensitivity, only two adaptive capacity sources). Therefore, we present analyses of uncertainty for the individual components where we can, but not for the final community vulnerability scores. All analysis was completed using R [80] in Rstudio [81].

## Results

### Ecological risk

**Fig 2:**
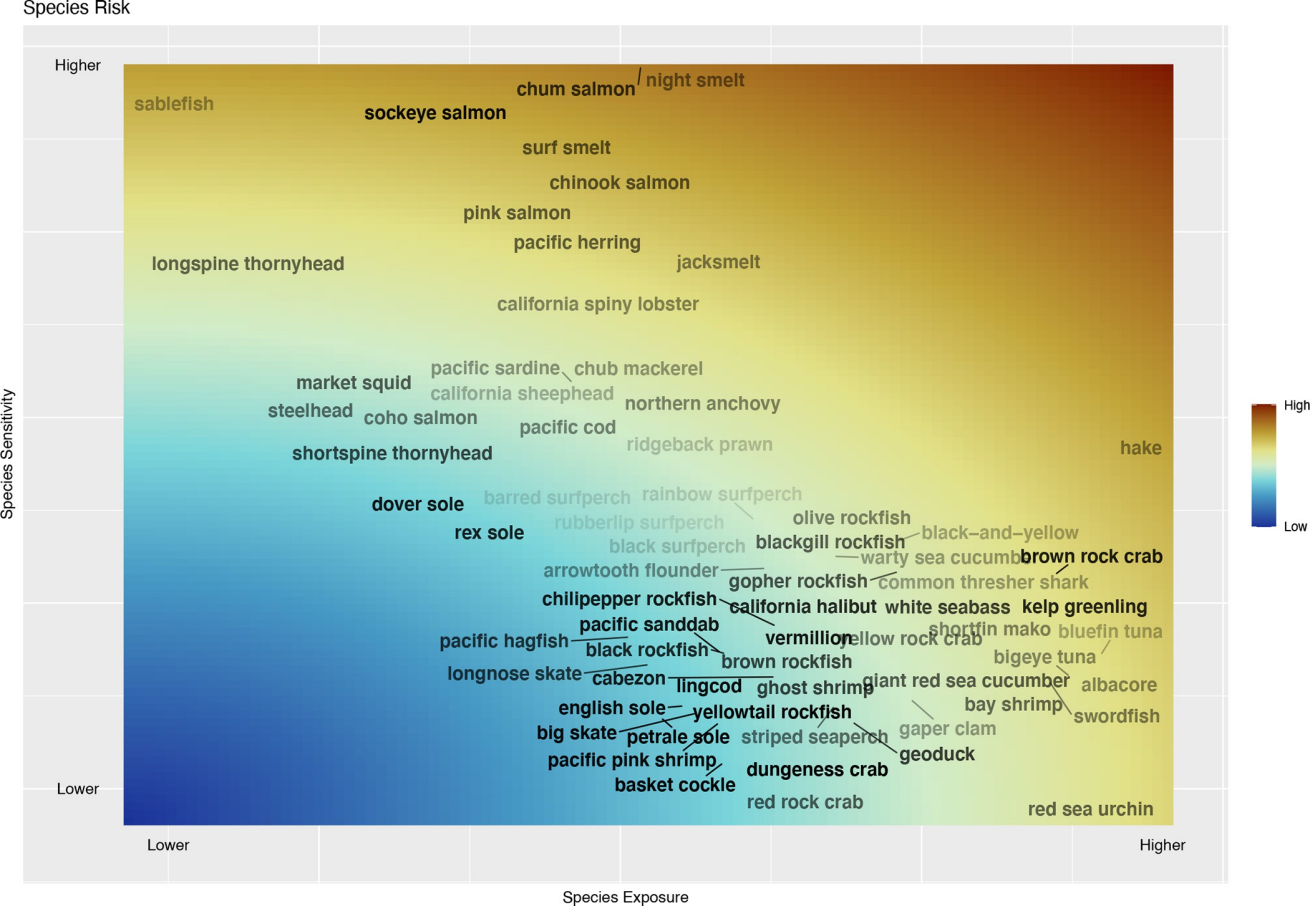
Ecological exposure and ecological sensitivity to climate changes for fisheries species. Ecological risk to climate change (changes in pH, temperature, chlorophyll, and oxygen) which is the Euclidean distance between ecological exposure and ecological sensitivity. Ecological exposure and sensitivity are averaged across the four climate variables (each ranging from 0 to 1) for each climate model and then averaged across three climate models for species in top 90% of landings (by weight) for US West Coast fishing communities. See S2 Table for individual species risk. Transparency of the name corresponds to standard deviation in exposure (more transparent equals higher deviation/uncertainty) across the three climate models (relative to the other species groups).

Overall, across climate models and climate variables (pH, oxygen, chlorophyll, and temperature), the species most at risk from projected climate change on the US West Coast include Pacific hake (*Merluccius productus*), smelts (*Atherinopsis californiensis*, *Spirinchus starksi*, *Hypomesus pretiosus*), salmon (*Oncorhynchus* spp.), Pacific herring (*Clupea pallasii*), spiny lobster (*Panulirus interruptus*), sablefish (*Anoplopoma fimbria*), sharks (*Alopias vulpinus*, *Isurus oxyrinchus*), albacore (*Thunnus alalunga*), bluefin tuna (*Thunnus orientalis*), and red sea urchins (*Strongylocentrotus franciscanus*) (Fig 2, S2 Table). Some of these species such as Pacific hake and sea urchins are at risk because of their high exposure to climate change, while others (e.g, salmon, smelt, and Pacific herring) have high sensitivity to climate change because they experience a relatively narrow range of environmental conditions within the California Current ecosystem. Many of the top at risk species had high exposure and/or sensitivity to temperature (most smelt, hake, salmon) (S4 Table). The top 10 most at risk species also had high sensitivity to oxygen changes (except hake, but hake had high values for exposure and sensitivity to pH). A few most at risk species had high exposure to chlorophyl changes or sensitivity to pH changes (hake, sablefish and jack smelt), but no species in the top 10 at risk had high sensitivity to chlorophyl changes or high exposure to pH (but see spiny lobster and red sea urchin in the top 20).

### Community risk

**Fig 3:**
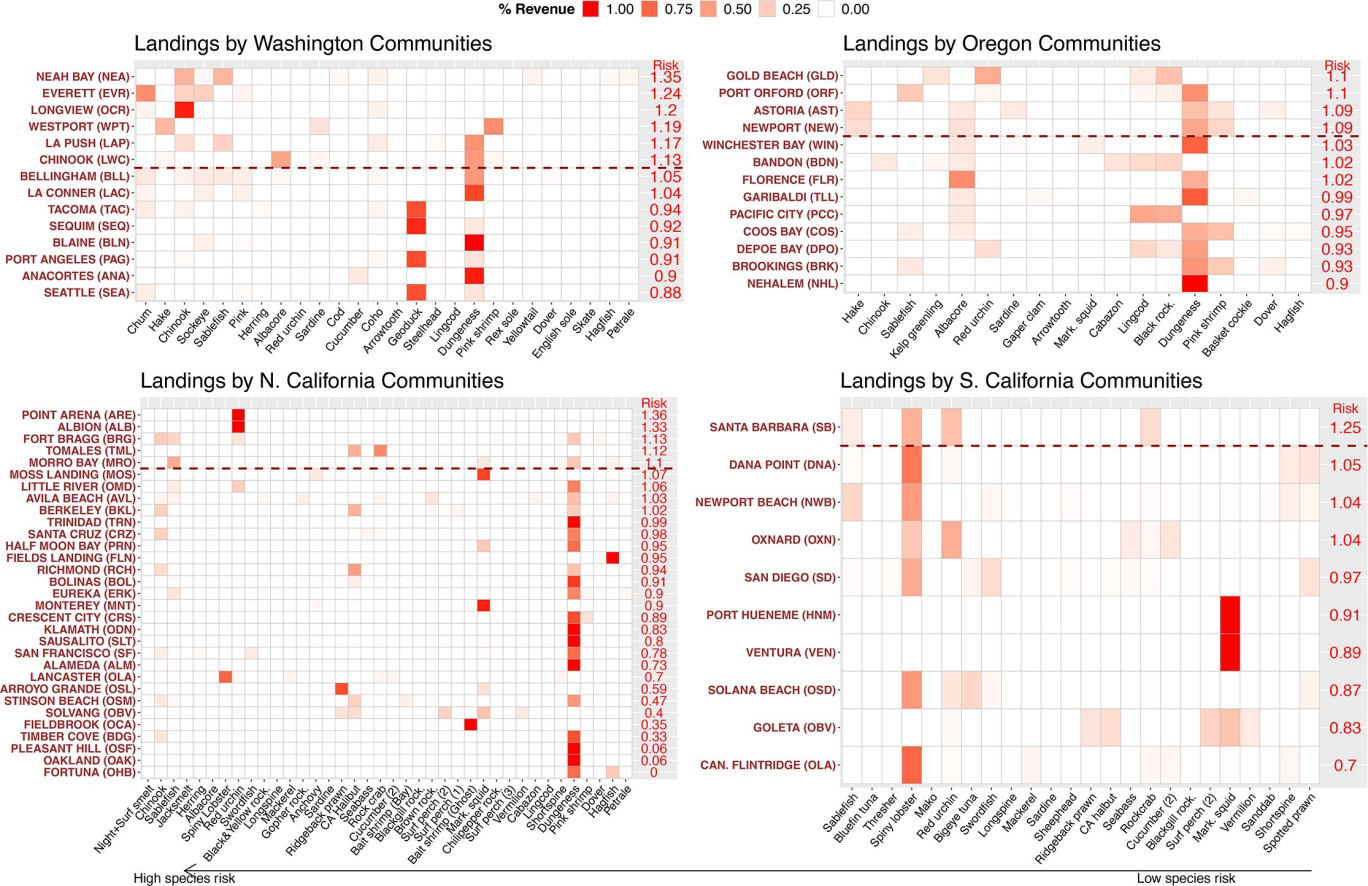
Percent revenue composition by community for species landed. Major fishery landings by community by state, where transparency of red is based on percent revenue for that species/catch group (solid red = greater percent revenue). Percent revenue is out of the total revenue for that community, for the species that were in the top 90% of landings for that community. Communities are ordered from highest risk (community exposure combined with community sensitivity [reliance]) to lowest (“Risk” on figure). For communities with the same landings composition (part of the same port group), a random community was picked and plotted (190 communities), to specifically show landings compositions that give high risk. Depending on the random community in each port group, risk will change due to variation in sensitivity but landings composition does not vary. Port group name abbreviations are in “()” and see S5 Table for full port group names. Species are plotted from highest to lowest ecological risk. Communities above the red dotted line are in the top 10 percentile for risk. Overall there are different combinations of species landings that lead to high community risk.

Communities such as Neah Bay WA, Everett WA, Longview WA, Westport WA, Point Arena CA, Albion CA, (Fig 3, S5 Table) are highly at risk to climate change because they are reliant on species that are at high ecological risk such as salmon, red sea urchins, or Pacific hake. However, other communities with economies that depend heavily upon fisheries are highly sensitive to climate impacts even though their catch of species at high ecological risk is relatively low (e.g., Tomales CA, La Push WA, Chinook WA/Ilwaco port; S5 Table). The majority of communities with 25% or more revenue from a single or multiple salmon species are in the top ten percent of at-risk communities (Fig 3, S5 Table). Most communities with greater than or approximately 20% of revenue from Pacific hake or sablefish were also in the top ten percent of at-risk communities. Communities with high percent revenue from red sea urchins are also highly at-risk when revenue from sea urchins is very high (near 100%, Point Arena and Albion, CA), or the communities’ catch is made up of a high percent revenue from multiple species with high ecological risk (for example, Santa Barbara–California spiny lobster, red sea urchin, and sablefish; Gold Beach, OR–kelp greenling [*Hexagrammos decagrammus*] and red sea urchins). Large catch of species somewhat at-risk ecologically, combined with high community sensitivity (i.e., economic dependence on fisheries), also leads to high community risk (Chinook, WA, albacore catch). The only other community in the top 10% for community risk, Tomales, CA, has reliance on a few species at moderate ecological risk but has high community sensitivity (see S5 Table).

Washington and Oregon have disproportionately higher numbers of at-risk communities compared to California. Communities in Washington and Oregon receive much of their revenue from species with high ecological risk; thus, a higher percent of communities in Washington and Oregon (46% and 29%) ranked at higher risk (i.e., top 10 percentile, risk > 1.077) to climate change than in California (5%) (S5 Table). This is especially true for Washington as 6 of the top 10 communities by risk are in Washington (other 4 in California). Revenue of greater ~20% from the highest risk species like salmon, hake, and/or sablefish (all in the top 10 species for ecological risk) leads to higher community risk, but most California communities have low percent revenue from these species. Communities in Northern California exhibit the greatest range and variability in community risk, with several communities in the lowest 10 percentile (Pleasant Hill, Oakland, Fortuna) and others in the highest (e.g., Point Arena, Albion, Fort Bragg), and still with many others in the middle (e.g., Arroyo Grande, Klamath, Fieldbrook) but with lower community risk than any of the communities in Oregon and Washington.

### Community adaptive capacity

**Fig 4:**
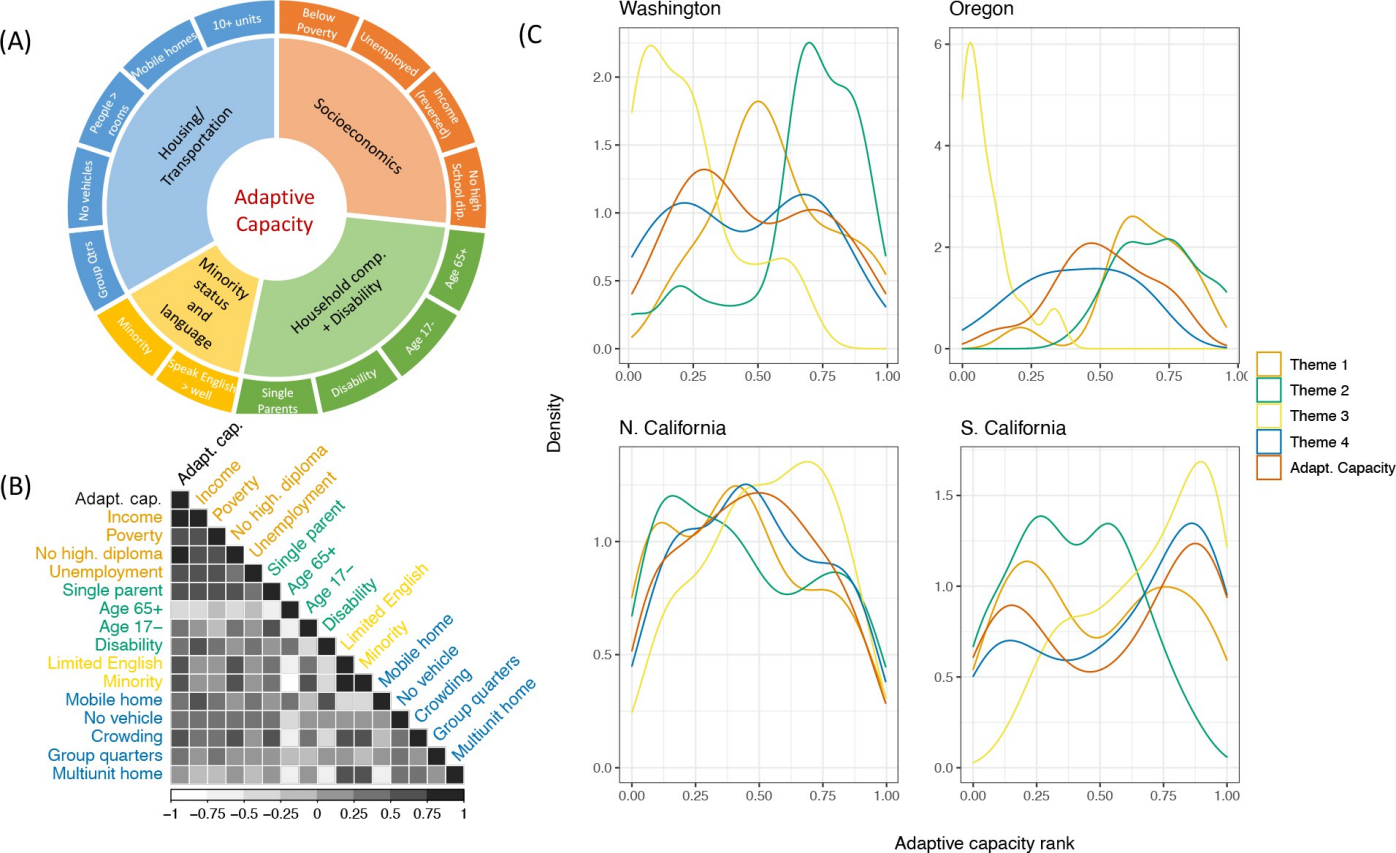
Social indicators and themes that make up adaptive capacity and relation to final adaptive capacity scores. (A) the four themes of adaptive capacity and individual indicators that make up each. Theme 1 is socioeconomic indicators (orange), theme 2 (green) is household composition and disability, theme 3 (yellow) is minority status and language, and theme 4 (blue) is housing/transportation. (B) The correlation between adaptive capacity and each individual indicator colored by theme. (C) The density distribution of scores for each theme and overall adaptive capacity (where greater values = lower adaptive capacity) for each geographic region.

Adaptive capacity in fishing communities was greatly influenced by socioeconomic factors overall, but other social themes that contributed most to adaptive capacity varied by region (Fig 4). Communities with the lowest adaptive capacity had the highest values for indicators such as high percent in poverty, low per capita income, high unemployment, and high percent with no high school diploma (S4A and S4B Fig, S6 Table). Adaptive capacity was most correlated with socioeconomics overall (Fig 4B). In Southern California communities with low adaptive capacity were also associated with minority status/language indicators, specifically percent minority and percent of the population that does not speak English well, as well as housing and transportation indicators (Fig 4C). Overall, our estimates of adaptive capacity were lowest for Southern California fishing communities (Fig 4, S5 Table). In addition to socioeconomic factors, Washington, Oregon, and Northern California fishing communities with low adaptive capacity also had relatively high values for household composition/disability indicators (Fig 4).

### Community vulnerability

**Fig 5:**
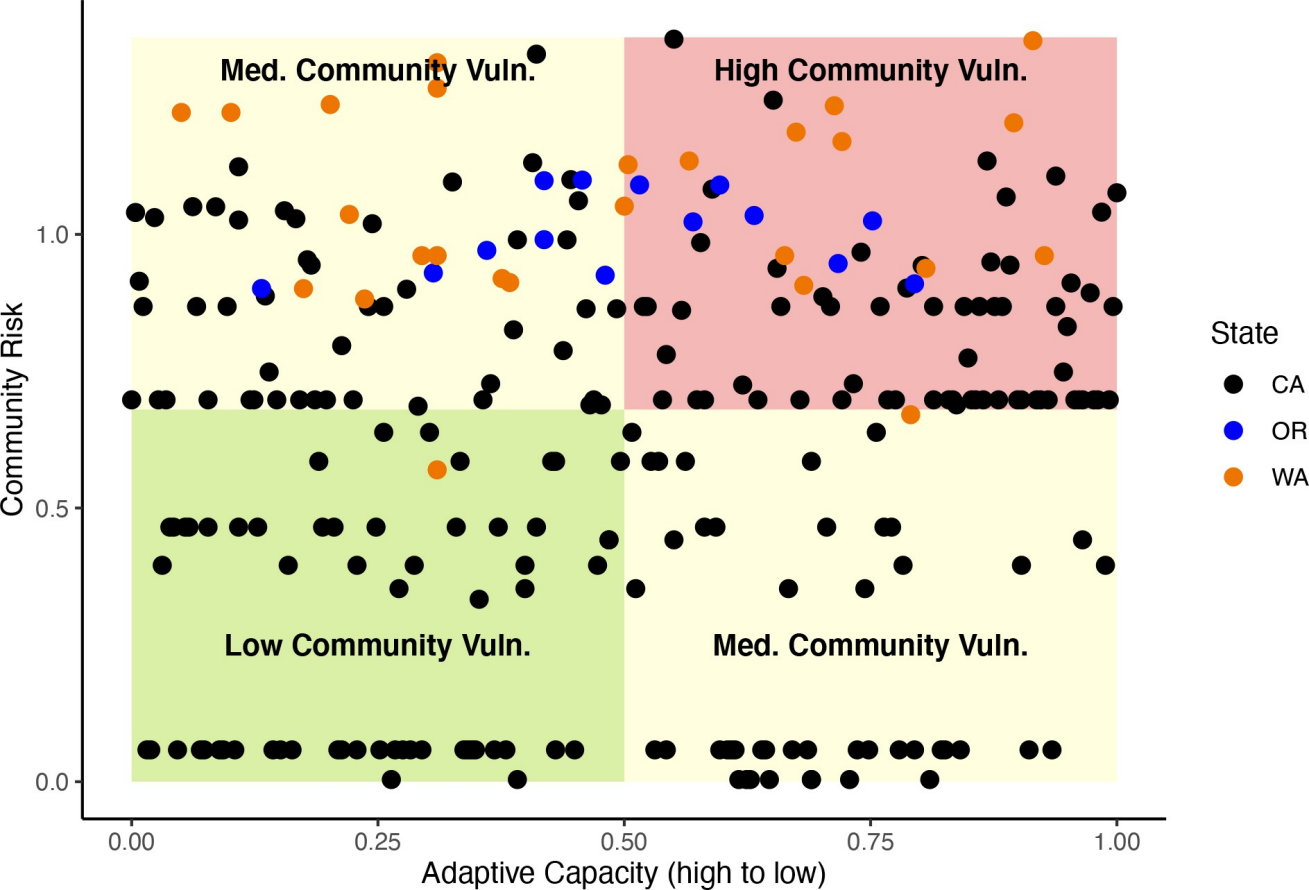
Community vulnerability as a function of community risk versus adaptive capacity (where greater values = lower adaptive capacity). Quadrants represent high, medium, or low community vulnerability where communities can have medium vulnerability either be having low adaptive capacity (and low risk) or high adaptive capacity but high risk. Points are color coordinated by state. All states have communities with high vulnerability but the most vulnerable communities are disproportionately represented in Washington and California.

**Fig 6:**
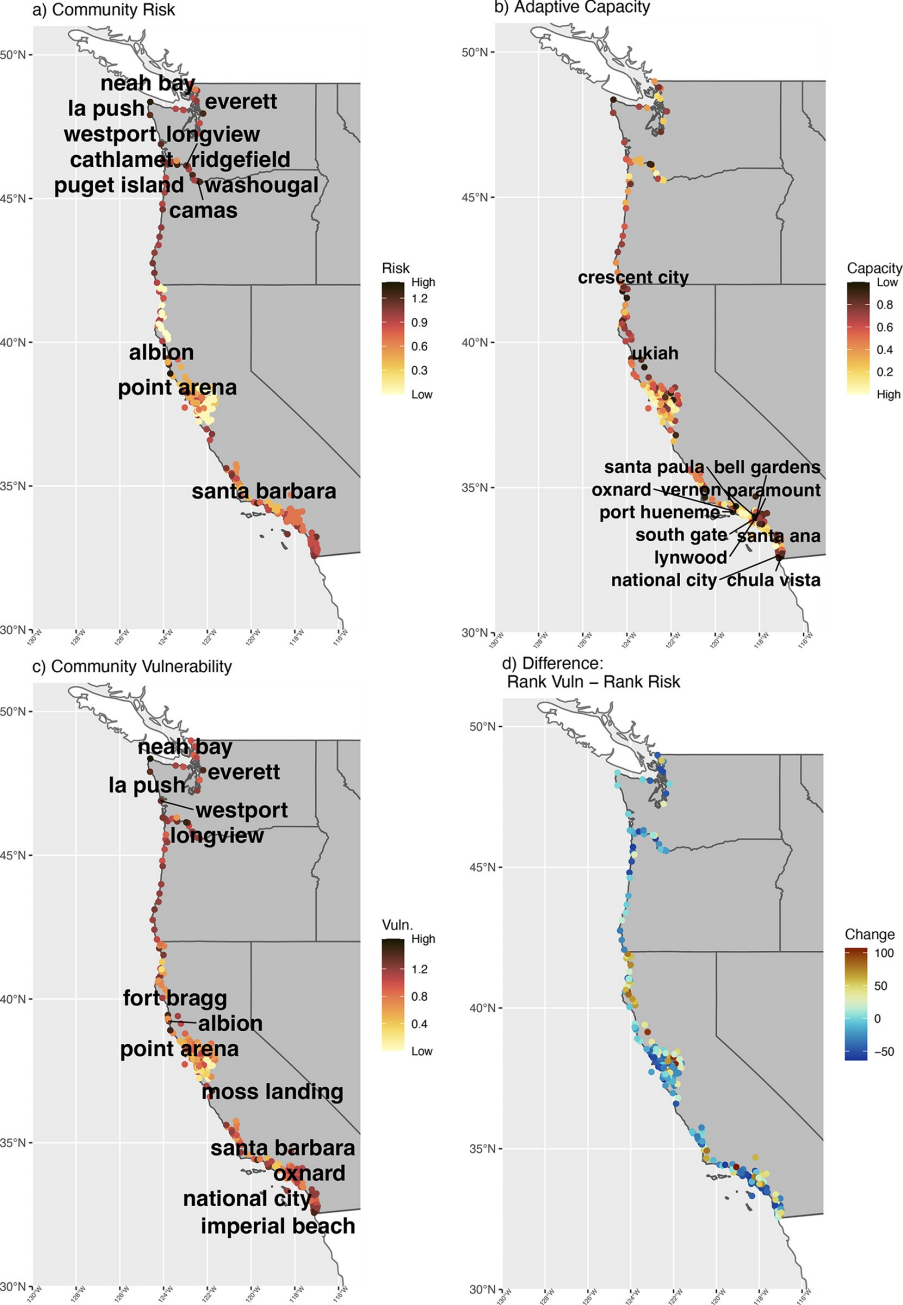
Top communities by risk, adaptive capacity, and vulnerability, as well as difference in rank scores between risk and vulnerability by community. Communities ranked by risk (top left) and adaptive capacity (top right), community vulnerability (bottom left) across the U.S. West Coast. Communities labeled are those in top 5 percentile of risk, adaptive capacity, or vulnerability. Considering social information (adaptive capacity) compared to solely ecological/economic data (risk) changes which communities are in the top for most imperiled, though others are ranked high across the board. Also, the “difference” (bottom right) is the rank position of the community based on vulnerability (#1 rank is most vulnerable) minus it’s rank position from risk.

Integrating community risk and adaptive capacity to estimate community vulnerability to climate change reveals that communities from each state rank in the highest quadrant of vulnerability but communities in Washington and California are the most vulnerable (Figs 5 and 6). The inclusion of adaptive capacity produces a much different picture of vulnerability compared to estimates of community risk that omit adaptive capacity (Fig 6).

In some cases, not including adaptive capacity masks communities that are imperiled. In California, for instance, National City ranks 27^th^ (out of 259) in risk, but when adaptive capacity is included, it rises to 3^rd^ (Fig 6, see S5 Table for other examples). Indeed, the inclusion of adaptive capacity exposes a number of California fishing communities that may be less resilient to climate change than expected when considering only community exposure and sensitivity (Fig 6d, S5 Table). Many communities in Southern California ranked lowest for adaptive capacity and had the highest values for social indicator Theme 3—minority status/language.

In other instances, the relatively high adaptive capacity of some communities appears to mitigate the risk inherent in their reliance on species at ecological risk from climate change. For example, a number of Washington communities along the Columbia River from the port group “Other Columbia River [OCR]” (e.g., Longview, Cathlamet, Puget Island, Washougal, Camas, and Ridgefield) are reliant on species such as salmon that are threatened by climate change. Thus, these communities were among the highest ranked communities for risk (Figs 3 and 6a), but their relatively high adaptive capacity mitigates some of the ecological risk, lowering their overall vulnerability (Fig 6, S5 Table).

Some communities ranked high no matter if we considered community risk or community vulnerability. These include cases where the community relies heavily on species at high ecological risk from climate change and adaptive capacity is low (e.g., Neah Bay, WA; Point Arena, CA amongst others; Fig 6, S5 Table). Alternatively, many communities that make up the “Other San Francisco” port group focus on fishery species that are at lower ecological risk due to climate change, and they have relatively high adaptive capacity, and ranked the lowest for both community risk and vulnerability (S5 Table).

Note, there are two communities where we miss high valued species in the landings composition because we looked at the top 90% of landings by weight instead of revenue. These are Westport, WA where using species composition by revenue instead would likely lower the vulnerability of the community because the missing species are less at risk species, and Fields Landing, CA where vulnerability would likely remain the same since the missing species are of similar risk as those used.

## Discussion

Vulnerability is a boundary concept (one that translates and is understood across disciplines) [82] grounded in theory that spans the social and biophysical sciences [83], and is a critical element of ecosystem-based management [84–86]. Our social-ecological vulnerability assessment of fishing communities of the California Current revealed a number of fishery species at risk from climate change, pinpointed communities that are highly dependent on these species, and highlighted communities with varying capacity to adapt to disruptions in fisheries species and fisheries economies. Our results emphasize that focusing solely on either the ecological risk to fishery species, the economic dependence of communities on fishing or the adaptive capacity of communities provides an incomplete evaluation of the potential vulnerability of fishing communities to climate change. Therefore, similar to recent conclusions by Payne et al [39], there is likely no single solution that can be applied to address vulnerability moving forward. Indeed, our work highlights that what we observe is not climate vulnerability in itself, but vulnerability revealed through our method of questioning (cf. [87]). Reducing the vulnerability actually experienced by the 259 U.S. West Coast communities we investigated requires that the breadth of an assessment aligns with the full set of factors that contribute to vulnerability.

Our work reveals that fishing communities across all states of the West Coast of the U.S. are highly vulnerable to climate change. These include towns such as Neah Bay and Longview in Washington State and National City, Oxnard, and Imperial Beach in California where relatively low adaptive capacity contributes to increased vulnerability. Thus, increasing adaptive capacity and economic diversification of fisheries could be beneficial to such communities (see also [88]). Our results suggested that adaptive capacity rankings were mainly driven overall by socio-economic metrics (adaptive capacity correlated most with socioeconomics than other social indicators), and, indeed, economic assets are often cited as a key component of adaptive capacity (e.g., [30, 89, 90]). As such, management strategies that build financial assets as well as foster economic flexibility can improve adaptive capacity. For instance, quota swapping/interchangeable quota, and side payments have been discussed as potential opportunities to increase flexibility [91, 92]. Additionally, Barnes and colleagues [93] show that beyond building financial assets, attention to social networks, education, risk perception and fostering agency can greatly enhance adaptive capacity of fishing communities.

Our work also shows that certain communities in California are moderately vulnerable to climate change because, although they exhibit low community risk, they have low adaptive capacity. Thiault et al. [88] suggest that such communities have high latent vulnerability. For instance, our analysis suggests that Santa Paula, California has relatively low vulnerability because of a high percent of landings of California market squid (*Doryteuthis opalescens*)—a species with relatively low exposure to the ecological impacts of climate change (within this study). However, the relatively low adaptive capacity of Santa Paula means that predicted declines in squid populations (e.g., [94]) or a focus on more at-risk species (e.g. sea urchins), could reveal an underlying vulnerability resulting from the relatively low adaptive capacity of the community. In cases such as these, investments in adaptive capacity will reduce latent vulnerability, allow communities to become better prepared for future impacts, and improve the resilience of these communities (see [95]).

In contrast, other communities are moderately vulnerable to climate change because they exhibit high levels of community risk, but have high adaptive capacity. The high community risk mainly results from the dependence of these communities on species that are at high risk from climate change. These communities are “potential adapters” [88]. Although these communities are vulnerable, they have the capacity to diversify the species they target (e.g., [96]), invest in emerging fisheries [97], or expand into other maritime ventures [98]. As species’ distributions shift [5, 6, 99], communities may be able to adapt and target species that were previously not easily accessible [100]. For example, predicted shifts in Pacific sardine (*Sardinops sagax*) distribution could lead to increased catch of this species in the northernly portion of the California Current system [66, 101], a potentially adaptative response as other highly at risk species that these communities target become less accessible. However, as noted, certain socioeconomic factors (see [102]) and/or management regulations and flexibility [100, 103] may influence fisher ability to take advantage of such shifts.

Our analysis suggests that Indigenous communities may be particularly vulnerable to future climate change (see also, [104])—some of the communities that we scored among the most vulnerable have large Indigenous populations (e.g., Neah Bay and La Push, home of the Makah Tribe and Quileute Nation, respectively). Cultures of coastal Indigenous people are closely tied to particular marine species [105, 106]), and food security of these communities depends on access to seafood [107]. For example, our results showed high ecological risk for salmon species—a major food source for many West Coast tribes and species that play significant cultural and social roles in Indigenous communities [108, 109]. We did show that adaptive capacity led to lower vulnerability compared to risk for the port group “Other Columbia River ports” that likely includes many Indigenous communities and tribal landings on salmon, but this may illustrate the challenges with using broad geographic census tract information to represent community adaptive capacity (see discussion below), where multiple cultural communities may be represented.

Importantly, our assessment of adaptive capacity relied on census data and thus may provide an incomplete portrayal for Indigenous communities. Measures of adaptive capacity based on census data directly or indirectly emphasize economic assets. In particular, our use of a standard, empirical data-based index for adaptive capacity is certainly imperfect for Indigenous communities where locally value-based, cultural indicators may more accurately represent Indigenous community resilience and adaptability (see [110]). Additionally, domains of adaptive capacity such as flexibility, social organization and learning [93] are not captured by our approach. Also, because communities are socially-constructed based on both shared meaning and geography, using census data as a proxy for communities is imperfect. Nonetheless, we chose to use census data because these data are widely used due to their coverage and availability, and because they are available for the entire U.S. West Coast at a scale relevant to policy and management.

Indeed, our work both follows from and utilizes some of the national, cross-regional community social indicators, developed from census and fisheries data nationally, and provided to the public and to fishery management councils (FMCs) by National Marine Fisheries Service social scientists as part of a broad move toward enhancing and expanding social metrics available for integrated social-ecological approaches [54, https://www.fisheries.noaa.gov/national/socioeconomics/social-indicators-coastal-communities]. In both our reproducible use of census data and our decision to operationalize our approach at the scale of the census-designated place, the same geographic unit in use for the NMFS’ community social indicators approach, we have sought to augment the NMFS community indicators with additional layers of analysis appropriate to more specific interests in climate vulnerabilities and impacts. Accordingly, while our work is distinct from similar climate-oriented innovations for NMFS social indicators in other fishery management regions [37], it is generally in keeping with the aim of developing the community social vulnerability indicators and information available for a framework that accounts for climate exposure, sensitivity and risk. In effect, our approach presents a replicable means of expanding the utility of NMFS social indicators as potential tools for management, with particular attention to climate change. Additionally, by using the more universal CDC index, future work could look at vulnerability of geographic communities across multiple climate stressors (see for instance [30] and use of the CDC index to determine community vulnerability to wildfires).

A caveat to developing a high-level, quantitative, automatable, tool for assessing vulnerability for communities across ecological, economic, and social dimensions is that no particular focus is put on any one dimension and detailed specifics may be overlooked when working at the scale of this study. On the ecological side, our high-level analysis did not account for indirect impacts of climate change on species or impacts across species life stages which is illustrated by Dungeness crab results. Specifically, Dungeness crab larvae are at risk to climate change [111, 112], but our analysis focused on adult life stages and showed low risk for Dungeness crab. Moreover, studies that incorporated food web effects found risk to Dungeness crabs due to effects on prey from pH changes [113]; but such indirect effects were not accounted for here. An additional caveat is that there is widespread concern about shifts in species distributions due to climate change [114], which we did not model explicitly here (though is indirectly captured in changes in climate variables experienced by a species); such work remains an important area of future research. Our assessment also does not include species in Alaska targeted by West Coast fishermen which is outside the scope of our study. Finally, our method for calculating ecological risk does not take into account seasonal shifts in distributions for migratory species; we used full ranges (with probability of occurrence >0.4), potentially resulting in an overestimate or underestimate of risk for these species.

For adaptive capacity, the social indicators we used are at a geographic community scale and therefore, may not capture specific qualities of particular persons engaged in fishing. Fishers themselves likely have varying adaptive capacity at an individual level or fleet level, outside of the geographic community as a whole, and future work should assess more specified adaptability. Moving forward, assessments of vulnerability could incorporate information from surveys aimed at eliciting individual fishers’ perceptions on adaptive capacity, since information that captures personal experiences likely better captures an individual person’s ability to adapt [115] (and see [116]), compared to broad community assessments such as the census. Though the scope of our analysis may exaggerate or understate vulnerabilities or risk for certain communities or species, our results were robust across multiple sources of uncertainty, data (three climate models, two adaptive capacity indicators, revenue versus landings data) and methods of calculations (see S1–S3 Figs) and therefore, represents an overall picture of communities likely to be vulnerable to future climate change.

Confidentiality requirements for specific landings data led to the need to remove certain ports where a higher percent of landings were confidential, including the removal of certain communities that are potentially highly vulnerable. Approximately 16% of communities were removed because of a high percent of confidential landings data. Though we do not have all landings (and therefore can’t calculate exposure), we can calculate sensitivity and adaptive capacity for the removed communities. Of those communities, four and seven had high sensitivity and low adaptive capacity respectively (90th percentile of all communities) and thus have a higher likelihood of having high vulnerability, though it is ultimately dependent on the unknown exposure (see S7 Table). In particular, Taholah, WA, a community with a large Indigenous population, scored high for sensitivity and low for adaptive capacity, consistent with our findings about the climate change vulnerability of coastal Indigenous communities. One of our objectives of this work was to create a reproducible, automatable assessment that is generally aligned with the scale of the federal fisheries governing body, the PFMC, so that the assessment may be used by the Council. Should the PFMC choose to use this assessment methodology, they may want to work with state Fish and Wildlife agencies to access certain confidential fisheries data under their jurisdiction in order to gain a broader understanding of fishing community vulnerability. Additionally, further work could focus specifically on communities with majority confidential landings but high sensitivity and low adaptive capacity.

The recent release of the sixth assessment from the IPCC [117] emphasizes the urgent need to strengthen the ability of coastal communities to adapt to our changing oceans. Climate change is disrupting fisheries across the world, impacting individual livelihoods and testing the resilience of coastal communities. Our results reveal that not all fishing communities are equally threatened by climate change, and mechanisms underlying disparities in climate vulnerability differ: ecological, economic and social factors each influence vulnerability but their contribution varies among communities. Recognition that the foundation of climate vulnerability varies among communities highlights the need to consider a diversity of solutions that have the potential to reduce the exposure and sensitivity while increasing the adaptive capacity of communities. Also, which communities are vulnerable and how they experience that vulnerability may change through time, or may change as we acquire more knowledge on climate change impacts. Importantly, climate change disproportionately burdens under-resourced and marginalized communities and can exacerbate existing inequities; however, existing approaches to climate risk in fisheries do not always adequately reflect reality. The methods we develop here provide a systematic, rigorous and scalable means to identify those fishing communities that are excessively affected by climate change. With analyses such as the one we present here, our hope is that we can move swiftly to chart a course to a more resilient and just future for all those who depend on fisheries and healthy oceans.

## Supporting information

S1 Appendix Supporting methods. (DOCX)

S1 Fig Correlations between ecological risk derived from three different climate models. Correlations between ranked estimates of species ecological risk to climate change derived from three different downscaled climate projection models—Geophysical Fluid Dynamics Laboratory Earth System’s Model GFDL-ESM2M ([68,69]; referred to as “GFDL”), the Met Office Hadley Centre Earth Systems Model HadGEM2-ES ([70]; “HAD”), and the Institut Pierre Simon Laplace Model IPSL-CM5A-MR ([71]; “IPSL”). Correlation coefficient is given in red.
(TIF)

S2 Fig Correlations between community exposure derived from three different climate models. Correlations between ranked estimates of community exposure to climate change derived from three estimates of species risk from three different downscaled climate projection models—Geophysical Fluid Dynamics Laboratory Earth System’s Model GFDL-ESM2M ([68,69]; referred to as “GFDL”), the Met Office Hadley Centre Earth Systems Model HadGEM2-ES ([70]; “HAD”), and the Institut Pierre Simon Laplace Model IPSL-CM5A-MR ([71]; “IPSL”). And correlation between ranked community exposure estimates when species risk is weighted by percent landings by species versus percent revenue. Correlation coefficient is given in red.
(TIF)

S3 Fig Correlations between indices of adaptive capacity. Correlation between two ranked estimates of adaptive capacity via social indicators–the CDC index [55] and the NOAA index from the Integrated Ecosystem Assessment for the California Current [52] for U.S. West coast communities (correlation coefficient in red).
(TIF)

S4 Fig **A.** Social indicator ranked scores for communities in Washington and Oregon. Percent rank scores for each social indicator theme from the CDC that make up adaptive capacity for each fishing community in Washington and Oregon, ordered from least adaptive (top) to most adaptive community (bottom). Top 10 percent of least adaptive communities are labeled in red. Theme 1 is socioeconomic indicators, Theme 2 is made up of household composition/disability indicators, Theme 3 consists of minority status/language indicators and Theme 4 is community housing and transportation indicators. Percent ranks are not rescaled by state so still comparable across state. For every state, all communities with low adaptability rank high for theme 1, but Southern California is the only location where least adaptable rank high for theme 3, and these communities have lowest adaptability overall. **B.** Social indicator ranked scores for communities in Northern and Southern California. Percent rank scores for each social indicator theme from the CDC that make up adaptive capacity for each fishing community in Northern and Southern California, ordered from least adaptive (top) to most adaptive community (bottom). Top 10 percent of least adaptive communities are labeled in red. Theme 1 is socioeconomic indicators, Theme 2 is made up of household composition/disability indicators, Theme 3 consists of minority status/language indicators and Theme 4 is community housing and transportation indicators. Percent ranks are not rescaled by state so still comparable across state. For every state, all communities with low adaptability rank high for theme 1, but Southern California is the only location where least adaptable rank high for theme 3, and these communities have lowest adaptability overall.
(TIF)

S1 Table Species that make-up the top 90% of landings for West Coast fishing communities. Species (common name, scientific name, and pacFIN code) in the top 90% of landings for fishing communities on the US West Coast, for ports where <20% of catch is confidential. For catch that was labeled as an “unspecified” group (see PacFIN code column, https://pacfin.psmfc.org/pacfin_pub/data_rpts_pub/code_lists/sp_tree.txt), species that make up all unspecified species groups are listed and numbers indicate which are grouped together. Primary habitat (benthic [or demersal] vs. pelagic) determines which climate variables (for example surface temperature versus bottom temperature) are used when calculating ecological risk for each species (see supplemental information).
(DOCX)

S2 Table Species-specific ecological exposure, sensitivity, and risk derived from three climate models. Ecological exposure, sensitivity, and risk (Euclidean distance between exposure and sensitivity) of species to climate change, averaged across exposure and sensitivity to temperature, pH, oxygen, and chlorophyll, using three different climate models (GFDL, HAD, and IPSL), and average exposure, sensitivity, and risk across the three models for all species (in the top 90% of landings for communities and species that were not removed due to missing data or other reason, see supplemental information). Ordered from most to least ecologically at risk (Risk rank).
(DOCX)

S3 Table Species-specific ecological exposure, sensitivity, and risk derived from three climate models for additional species. Ecological exposure, sensitivity, and risk of species to climate change, averaged across exposure and sensitivity to temperature, pH, oxygen, and chlorophyll, using three different climate models (GFDL, HAD, and IPSL) for a subset of species in the California Current including species caught by Indigenous populations. Average risk is the average across the three models.
(DOCX)

S4 Table Species-specific ecological exposure and sensitivity to each climate variable. Average exposure and sensitivity (across the three climate models, HAD, IPSL, GFDL) to each climate variable (temperature, pH, chlorophyll, and oxygen) for each species in the risk assessment. Species are order from most at risk (overall) to least.
(DOCX)

S5 Table Community exposure, sensitivity, adaptive capacity, and vulnerability for West Coast fishing communities. Fishing communities included in the analysis, pacFIN port group/name (https://pacfin.psmfc.org/pacfin_pub/data_rpts_pub/code_lists/pc_tree.txt) that each community is part of, axes of community vulnerability, and overall community vulnerability scores and rankings for each community. Community exposure is the average ecological risk (across climate models) weighted by percent revenue of species for each community. Adaptive capacity, based on social indicators, is calculated so that smaller values (closer to 0) equal greater adaptive capacity and greater values (close to 1) equal lower adaptive capacity. Risk is calculated as the Euclidean distance between exposure and sensitivity and vulnerability is the Euclidean distance between exposure, sensitivity, and adaptive capacity. Rank values are from most vulnerable, most at risk, or least adaptive, to least vulnerable, least at risk, or most adaptive, respectively.
(DOCX)

S6 Table Social indicator scores that make-up adaptive capacity for each West Coast fishing community. For each community, social indicators, percentile ranked, that make up adaptive capacity within four themes: 1) socioeconomic—persons below poverty, unemployed civilians, per capita income, persons with no high school diploma; 2) household composition/disability—persons age 65+, persons age 17 and less, noninstitutionalized population with a disability, number of single parent households; 3) minority status/language—persons of minority, persons older than 5 that don’t speak English well; and 4) housing/transportation—housing structures with 10+ units, estimates of mobile homes, households with more people than rooms, households with no vehicles, and persons in institutionalized group quarters. Communities are listed from lowest adaptive capacity to highest.
(DOCX)

S7 Table Community sensitivity and adaptive capacity for West Coast fishing communities removed from analysis because of confidential data (unable to calculate exposure). Fishing communities removed from analysis because of confidential landings data. Adaptive capacity, based on social indicators, is calculated so that smaller values (closer to 0) equal greater adaptive capacity and greater values (close to 1) equal lower adaptive capacity. Sensitivity is community economic reliance on commercial fishing. Values are from percentile ranks across all communities (included and removed).
(DOCX)

S1 Data (DOCX)
