# Supplementary material for: Social-ecological vulnerability of fishing communities to climate change: A U.S. West Coast case study
Source: PLoS One. 2022 Aug 17;17(8):e0272120. doi: 10.1371/journal.pone.0272120 (PMC9385011; doi:10.1371/journal.pone.0272120)
Supplement: S1 Appendix — (DOCX) [file pone.0272120.s001.docx]

Supplemental Information

Methods

*Data Cleaning*

*Landings data from PacFIN*

For some ports, a percentage of landings was confidential (species landed by <3 vessels). If a substantial portion of landings were confidential (>20%), those ports were removed from analysis as it is unknown which species contribute large proportions of landings/revenue. For ports with confidential landings <20%, those landings were removed, the port was used in analysis, and the percent of each species (by revenue or landings) was adjusted to be out of the new total after removing the confidential percentage. We did this because a species that is only landed by less than 3 vessels in the community is probably unlikely to be significant contribution to that communities catch and income.

Similar methods were used for PacFIN landings that could not be identified at a species level. For landings reported as “other” (examples: other mollusks, other urchins), it is difficult to know which specific species this refers to. In all cases, these landings made up <5% of the top 90%. Therefore, we removed these. The port of San Diego as well as “Other” San Diego ports (defined by PacFIN) also had “miscellaneous fish” landings. We removed this catch as we do not know which species make up this group. There were also landings listed as “nominal” followed by a specific species; these were assumed to be the specified species following pacFIN specifications (https://pacfin.psmfc.org/faqs/what-is-a-nominal-nom-fish-species/).

Additionally, there were also landings reported as “unspecified”, for example: unspecified hagfish, unspecified sea urchin, etc. For each of these, we contacted US Fish and Wildlife institutes in the state of the catch (Washington, Oregon, or California) to determine what species most likely constituted these landings based on where the landings occurred and/or gear type. See Table S1 for which species make up each unspecified group. If multiple species make up an “unspecified” group, the average of their risk is used when calculating exposure for each community.

*Climate drivers used*

We included additional species sensitivity and species exposure to pH and oxygen compared to temperature and chlorophyll alone [1] because these additional climate variables are likely to also impact species. We included pH as many species groups are likely to have negative effects to lower pH in the California Current including commercially important species [2,3], pH levels have been declining in the California Current, and there has been increased shell dissolution in lower trophic calcifying organisms [4,5]. We included oxygen as a climate factor because low oxygen can impact respiration and function [6] and declines in dissolved oxygen have been reported for the California Current [7,8]. Instances of expanding hypoxia (low oxygen) conditions have been observed in the California Current [9] and this vertical expansion of the oxygen minimum zone may impact pelagic species through habitat compression and community reorganization [7].

*Species distribution data – species removed*

For some species in the top 90% of landings by port, habitat spatial range/distribution is not readily available. Specifically, there was no distribution for Leopard shark (*Triakis semifasciata*), but this species was only present in top 90% of landings for one port and made up <5% of the top landings. We therefore removed this species. Also, an accurate distribution for Spotted prawn (*Pandalus platyceros*) was also not readily available but this species made up >10% of the top 90% of landings for certain ports. Therefore, we used risk for Pacific pink shrimp (*Pandalus jordani*) to be equal to the risk for Spot prawn as these species are both in the family Pandalidae. Pacific halibut (*Hippoglossus stenolepis*) was removed from analysis as only a small proportion of their habitat falls within the specified region and range of the climate projections used.

*Species primary habitat designation*

Species exposure and sensitivity to a climate factor will depend on the species primary habitat. For example, if a species is benthic, it will likely be primarily exposed and sensitive to changes in bottom temperature compared to changes in surface temperature. Therefore, for analyses, we needed to determine primary habitat (benthic vs. pelagic) for each species. We referred to fishbase.org [10] or Sealifebase.org [11] to determine main habitat but primary habitat for a few species was modified if one designation resulted in outlier values for exposure and/or sensitivity. For benthic species, we found exposure and sensitivity to bottom temperature and bottom pH, while for pelagic species we used surface temperature and surface pH. For oxygen, for benthic species, we found the exposure and sensitivity to bottom oxygen concentration and for pelagic species, exposure and sensitivity to changes in the depth of the OMZ (specifically the depth of the OMZ based on 3.5 ml l^-1^ dissolved oxygen threshold), since pelagic species are most likely impacted by the expanding zone of hypoxia. We used a cut-off of 3.5 because for species like tunas, dissolved oxygen less than 3.5 ml l^-1^ may result in stress symptoms and possible mortality at long-term exposure [12,13]. For chlorophyll, we found sensitivity and exposure for all species to changes in chlorophyll concentration integrated over the upper 50m of the water column.

It is difficult to designate a main habitat preference for certain species. Particularly hake (*Merluccius productus*) is thought to be benthic but does move upward in the water column at night to feed [14]. Designation of hake as benthic made hake an outlier (substantially larger species risk), therefore, we classified hake as pelagic which still results in high risk for this species but less extreme. We also removed yellowtail amberjack (*Seriola lalandi*) from the analysis at this stage because they constitute <2% of landings for only one port and our geographic range only captures a small portion of their full range and classification as “pelagic” (their primary habitat) resulted in outlier values as well because we do not capture the full extent of climate experienced by the species.

*Adaptive Capacity/CDC index*

We compared two indices that represent adaptive capacity - the CDC index [15] to the NOAA IEA social index [16]. The IEA social index is calculated from six indices based on Jepson and Colburn [17] that are known to be markers of adaptive capacity. These include: (1) personal disruption index – variables that might impact a person’s ability to work; (2) population composition index – demographic make-up of the community; (3) poverty index – including young and old population in poverty; (4) labor force structure index – represents engagement in the labor force; (5) housing characteristics index – represents characteristics of available housing; and (6) housing disruptions index – factors that disrupt the housing market possibly from changing home values. For both labor force structure and housing characteristics, the index is reversed so that for all indices, higher values equate to higher vulnerability (and so that higher index values equal lower adaptive capacity).

Data for each of the six indices is collated by NOAA each year based on data from the American Community Survey though certain components of the six indices are not included in every year. Because of this, we used averages of each index from the last four years (2014-2017) for final index scores. Therefore, some community values may only be one year of data if data for that community is not calculated every year. If we did not have data for any of the six indices for a community across the 4 years, that community was removed from analysis. We combined the six indices into a final index (i.e. the IEA social index) by first percentile ranking each index (so they are all on the same scale) across communities, summing scores across indices for each community, and then again percentile ranking the summed score across communities for a final social index score per community. As stated in the main text, the NOAA index is correlated to the CDC index used for adaptive capacity (R=0.94).

References

1. Samhouri JF, Earl L, Barcelo C, Bograd S, Brodeur R, Cianelli L, et al. Assessment of risk due to climate change for coastal pelagic species in the California Current marine ecosystem. In: Arvey CJ, Garfield N, Hazen EL, Williams GD, editors. California Current Integrated Ecosystem Assessment: Phase III Report. The California Current Integrated Ecosystem Assessment: Phase III Report. Available from http://www.noaa.gov/iea/CCIEA-Report/index; 2013.

2. Marshall KN, Kaplan IC, Hodgson EE, Hermann A, Busch DS, McElhany P, et al. Risks of ocean acidification in the California Current food web and fisheries: ecosystem model projections. Glob Chang Biol. 2017;23: 1525–1539. doi:10.1111/gcb.13594

3. Busch DS, McElhany P. Estimates of the Direct Effect of Seawater pH on the Survival Rate of Species Groups in the California Current Ecosystem. Gobler CJ, editor. PLoS One. 2016;11: e0160669. doi:10.1371/journal.pone.0160669

4. Bednaršek N, Feely RA, Reum JCP, Peterson B, Menkel J, Alin SR, et al. Limacina helicina shell dissolution as an indicator of declining habitat suitability owing to ocean acidification in the California Current Ecosystem. Proc R Soc B Biol Sci. 2014;281. doi:10.1098/rspb.2014.0123

5. Feely RA, Alin SR, Carter B, Bednaršek N, Hales B, Chan F, et al. Chemical and biological impacts of ocean acidification along the west coast of North America. Estuar Coast Shelf Sci. 2016;183: 260–270. doi:10.1016/j.ecss.2016.08.043

6. Cisneros-Montemayor AM, Cheung WWL, Ota Y. Predicting future oceans: Sustainability of ocean and human systems amidst global environmental change. Predicting Future Oceans: Sustainability of Ocean and Human Systems Amidst Global Environmental Change. Elsevier; 2019. doi:10.1016/C2018-0-02416-0

7. Bograd SJ, Castro CG, Di Lorenzo E, Palacios DM, Bailey H, Gilly W, et al. Oxygen declines and the shoaling of the hypoxic boundary in the California Current. Geophys Res Lett. 2008;35: n/a-n/a. doi:10.1029/2008GL034185

8. Peterson JO, Morgan CA, Peterson WT, Di Lorenzo E. Seasonal and interannual variation in the extent of hypoxia in the northern California Current from 1998-2012. Limnol Oceanogr. 2013;58: 2279–2292. doi:10.4319/lo.2013.58.6.2279

9. Chan F, Barth JA, Lubchenco J, Kirincich A, Weeks H, Peterson WT, et al. Emergence of anoxia in the California current large marine ecosystem. Science. American Association for the Advancement of Science; 2008. p. 920. doi:10.1126/science.1149016

10. Froese R, Pauly D, editors. FishBase. World Wide Web electronic publication. (06/2021). 2021. Available: www.fishbase.org

11. Palomares MLD, Pauly D, editors. SeaLifeBase. World Wide Web electronic publication. (08/2021). 2021. Available: www.sealifebase.org

12. Brill RW. A review of temperature and oxygen tolerance studies of tunas pertinent to fisheries oceanography, movement models and stock assessments. Fish Oceanogr. 1994;3: 204–216. doi:10.1111/j.1365-2419.1994.tb00098.x

13. Brill RW, Bigelow KA, Musyl MK, Fritsches KA, Warrant EJ. Bigeye Tuna (Thunnus Obesus) Behavior and Physiology and their Relevance to Stock Assessments and Fishery Biology. Sci Pap ICCAT. 2005. Available: https://www.iccat.int/Documents/CVSP/CV057_2005/n_2/CV057020142.pdf

14. Love M. Probably more than you want to know about the ﬁshes of the Paciﬁc Coast. Really Big Press, Santa Barbara, CA; 1996.

15. Centers for Disease Control [CDC] and Prevention/ Agency for Toxic Substances and Disease Registry/ Geospatial Research, Analysis and SP. CDC/ATSDR Social Vulnerability Index 2018 Database Washington, Oregon, and California. 2020 [cited 29 Apr 2020]. Available: https://www.atsdr.cdc.gov/placeandhealth/svi/data_documentation_download.html.

16. Harvey C, Garfield N (Toby), Williams G, Tolimieri N, Andrews K, Barnas K, et al. Ecosystem Status Report of the California Current for 2019-20: A Summary of Ecosystem Indicators Compiled by the California Current Integrated Ecosystem Assessment Team (CCIEA). 2020. doi:10.25923/E5RB-9F55

17. Jepson M, Colburn LL. Development of social indicators of fishing community vulnerability and resilience in the US Southeast and Northeast regions. U.S. Dept. of Commerce., NOAA Technical Memorandum NMFS-F/SPO-129, 64 p; 2013.
