# Supplementary figures and images for: Social-ecological vulnerability of fishing communities to climate change: A U.S. West Coast case study
Source: PLoS One. 2022 Aug 17;17(8):e0272120. doi: 10.1371/journal.pone.0272120 (PMC9385011; doi:10.1371/journal.pone.0272120)

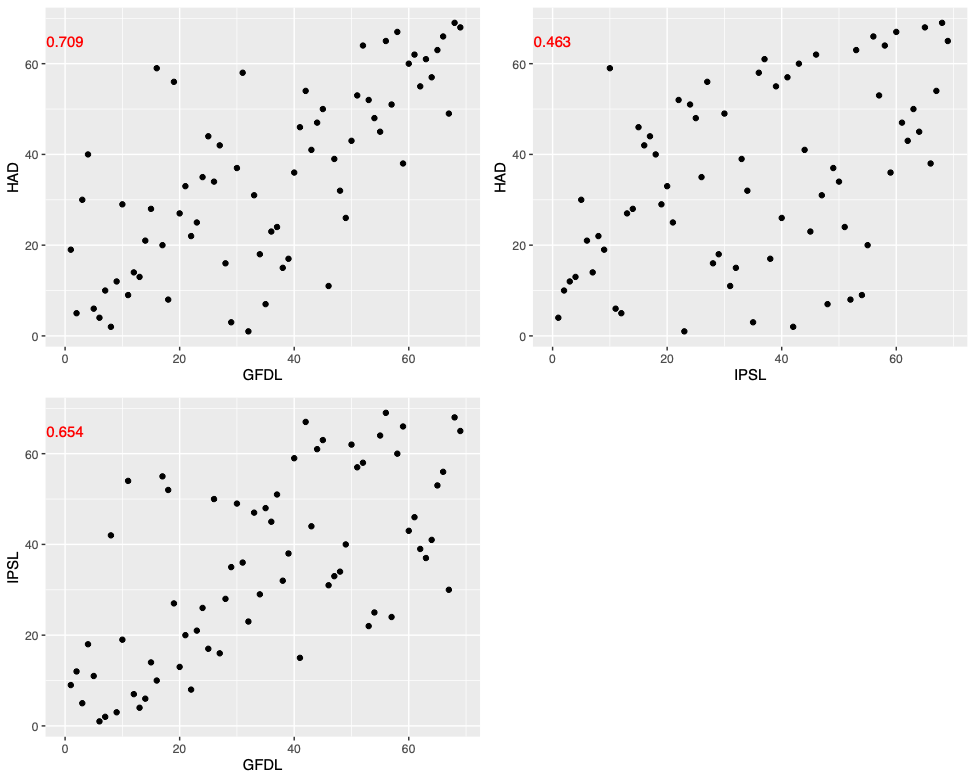

Supplement: S1 Fig — Correlations between ranked estimates of species ecological risk to climate change derived from three different downscaled climate projection models—Geophysical Fluid Dynamics Laboratory Earth System’s Model GFDL-ESM2M ([68,69]; referred to as “GFDL”), the Met Office Hadley Centre Earth Systems Model HadGEM2-ES ([70]; “HAD”), and the Institut Pierre Simon Laplace Model IPSL-CM5A-MR ([71]; “IPSL”). Correlation coefficient is given in red. (TIF) [file pone.0272120.s002.tif]

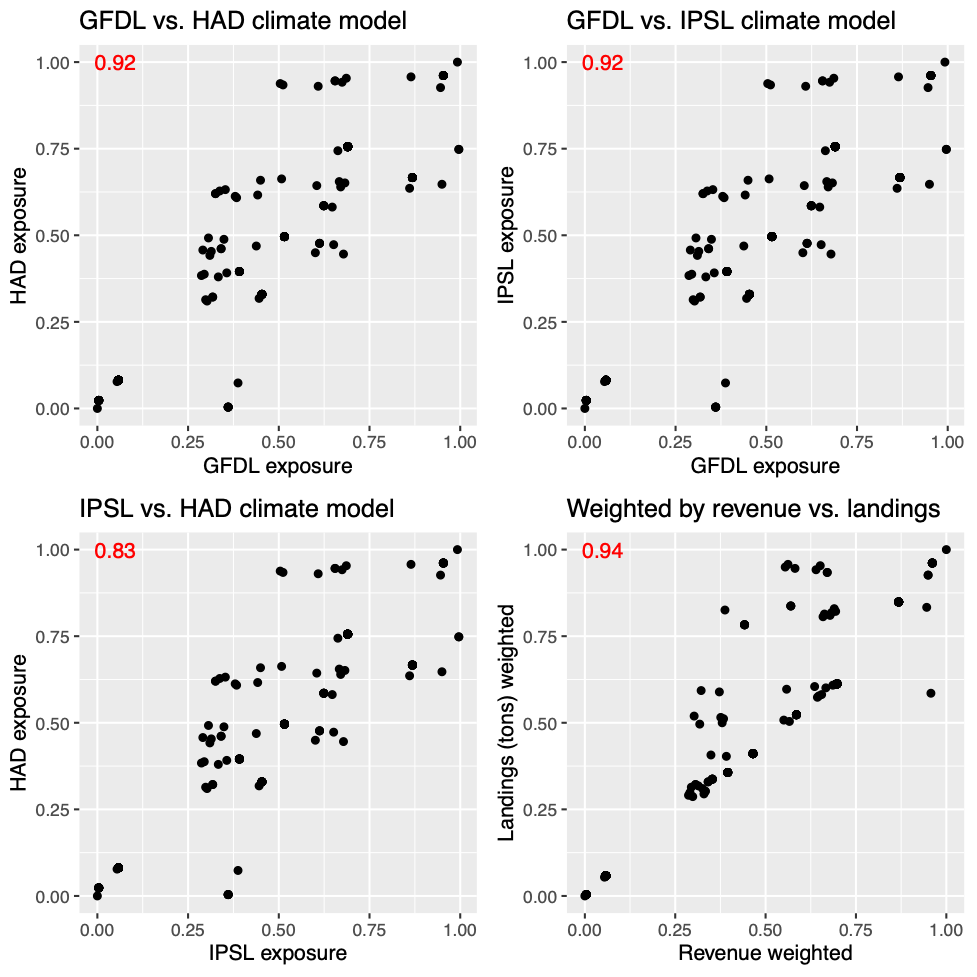

Supplement: S2 Fig — Correlations between ranked estimates of community exposure to climate change derived from three estimates of species risk from three different downscaled climate projection models—Geophysical Fluid Dynamics Laboratory Earth System’s Model GFDL-ESM2M ([68,69]; referred to as “GFDL”), the Met Office Hadley Centre Earth Systems Model HadGEM2-ES ([70]; “HAD”), and the Institut Pierre Simon Laplace Model IPSL-CM5A-MR ([71]; “IPSL”). And correlation between ranked community exposure estimates when species risk is weighted by percent landings by species versus percent revenue. Correlation coefficient is given in red. (TIF) [file pone.0272120.s003.tif]

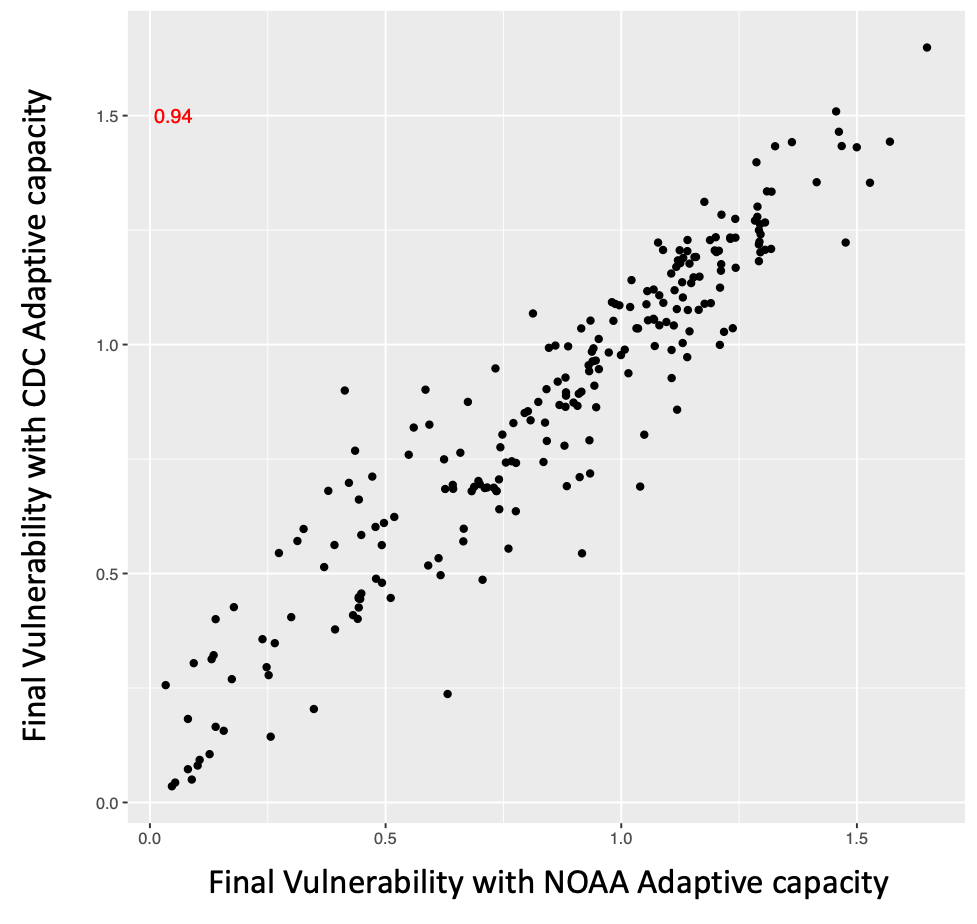

Supplement: S3 Fig — Correlation between two ranked estimates of adaptive capacity via social indicators–the CDC index [55] and the NOAA index from the Integrated Ecosystem Assessment for the California Current [52] for U.S. West coast communities (correlation coefficient in red). (TIF) [file pone.0272120.s004.tif]

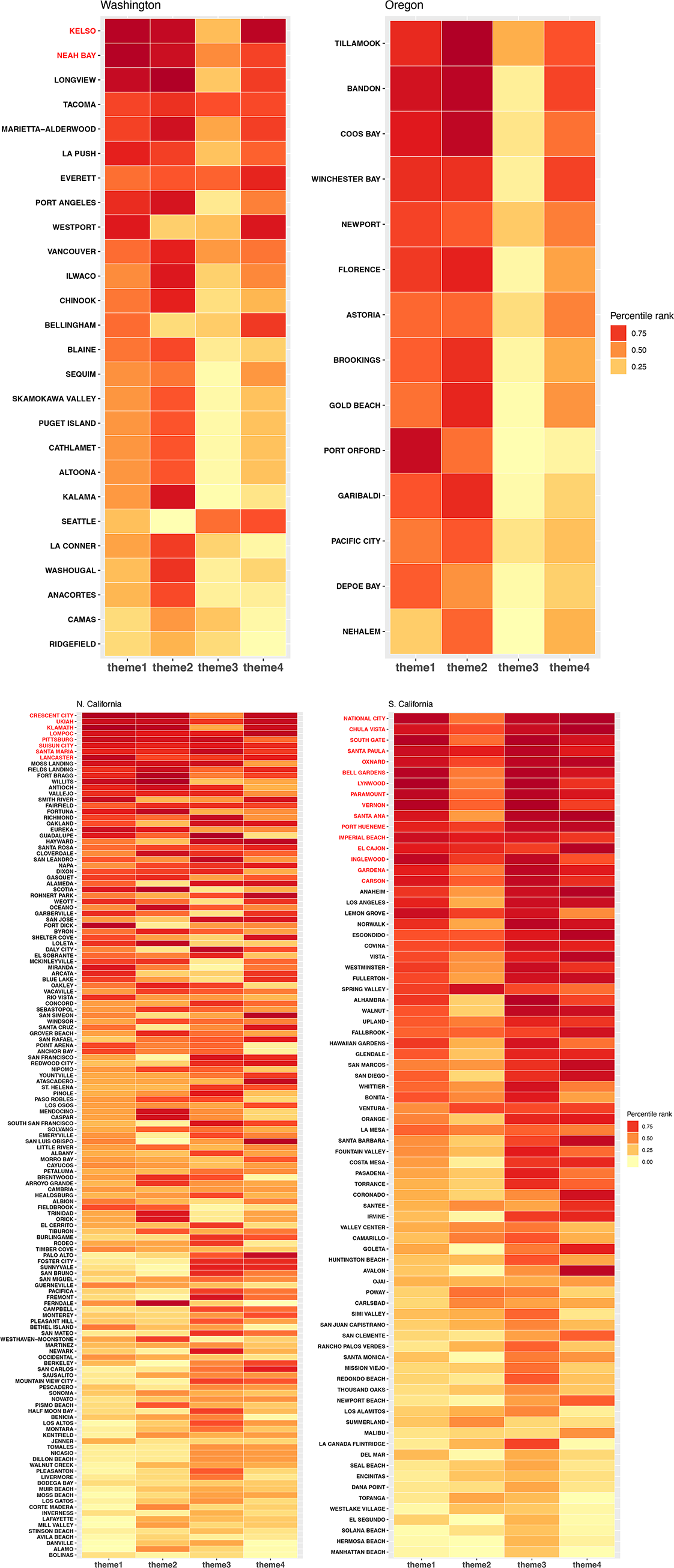

Supplement: S4 Fig — A. Social indicator ranked scores for communities in Washington and Oregon. Percent rank scores for each social indicator theme from the CDC that make up adaptive capacity for each fishing community in Washington and Oregon, ordered from least adaptive (top) to most adaptive community (bottom). Top 10 percent of least adaptive communities are labeled in red. Theme 1 is socioeconomic indicators, Theme 2 is made up of household composition/disability indicators, Theme 3 consists of minority status/language indicators and Theme 4 is community housing and transportation indicators. Percent ranks are not rescaled by state so still comparable across state. For every state, all communities with low adaptability rank high for theme 1, but Southern California is the only location where least adaptable rank high for theme 3, and these communities have lowest adaptability overall. B. Social indicator ranked scores for communities in Northern and Southern California. Percent rank scores for each social indicator theme from the CDC that make up adaptive capacity for each fishing community in Northern and Southern California, ordered from least adaptive (top) to most adaptive community (bottom). Top 10 percent of least adaptive communities are labeled in red. Theme 1 is socioeconomic indicators, Theme 2 is made up of household composition/disability indicators, Theme 3 consists of minority status/language indicators and Theme 4 is community housing and transportation indicators. Percent ranks are not rescaled by state so still comparable across state. For every state, all communities with low adaptability rank high for theme 1, but Southern California is the only location where least adaptable rank high for theme 3, and these communities have lowest adaptability overall. (TIF) [file pone.0272120.s005.tif]
