# Supplementary material for: Social-ecological vulnerability of fishing communities to climate change: A U.S. West Coast case study
Source: PLoS One. 2022 Aug 17;17(8):e0272120. doi: 10.1371/journal.pone.0272120 (PMC9385011; doi:10.1371/journal.pone.0272120)
Supplement: S1 Table — Species (common name, scientific name, and pacFIN code) in the top 90% of landings for fishing communities on the US West Coast, for ports where <20% of catch is confidential. For catch that was labeled as an “unspecified” group (see PacFIN code column, https://pacfin.psmfc.org/pacfin_pub/data_rpts_pub/code_lists/sp_tree.txt), species that make up all unspecified species groups are listed and numbers indicate which are grouped together. Primary habitat (benthic [or demersal] vs. pelagic) determines which climate variables (for example surface temperature versus bottom temperature) are used when calculating ecological risk for each species (see supplemental information). (DOCX) [file pone.0272120.s006.docx]

| **PacFIN code** | **Common name** | **Scientific name** | **ITIS Serial #** | **Notes** | **Benthic vs. pelagic** (Most from [1], unless noted) |
| --- | --- | --- | --- | --- | --- |
| ALBC | Albacore | Thunnus alalunga | 172419 |  | Pelagic |
| ARTH | Arrowtooth flounder | Atheresthes stomias | 172862 |  | Benthic [2] |
| BLCK | Black Rockfish | Sebastes melanops | 166727 |  | Benthic [3] |
| BLGL | Blackgill rockfish | Sebastes melanostomus | 166728 |  | Benthic [4] |
| BRWN | Brown rockfish | Sebastes auriculatus | 166708 |  | Benthic [4] |
| BTNA | Bluefin tuna | Thunnus orientalis | 172422 | Thunnus thynnus | Pelagic |
| BYEL | Black-and-Yellow Rockfish | Sebastes chrysomelas | 166773 |  | Benthic [5] |
| CBZN | Cabezon | Scorpaenichthys marmoratus | 692068 |  | Benthic [5] |
| CHLB | California Halibut | Paralichthys californicus | 172743 |  | Benthic |
| CHNK | Chinook salmon | Oncorhynchus tshawytscha | 161980 |  | Pelagic |
| CHUM | Chum salmon | Oncorhynchus keta | 161976 |  | Pelagic |
| CKLE | Basket cockle | Clinocardium nuttallii | 80873 |  | Benthic [6] |
| CLPR | Chilipepper rockfish | Sebastes goodei | 166722 |  | Benthic [7] |
| CMCK | Chub Mackerel | Scomber japonicus | 172412 |  | Pelagic |
| COHO | Coho salmon | Oncorhynchus kisutch | 161977 |  | Pelagic |
| DCRB | Dungeness crab | Cancer magister | 98675 | Metacarcinus magister | Benthic [8] |
| DOVR | Dover sole | Microstomus pacificus | 172887 |  | Benthic [2] |
| EGLS | English sole | Parophrys vetulus | 172921 |  | Benthic |
| ETNA | Bigeye tuna | Thunnus obesus | 172428 |  | Pelagic |
| GCLM | Gaper clam | Tresus capax | 80955 |  | Benthic [9] |
| GDUK | Geoduck | Panopea abrupta | 81779 |  | Benthic [10] |
| GPHR | Gopher rockfish | Sebastes carnatus | 166767 |  | Benthic [5] |
| KLPG | Kelp greenling | Hexagrammos decagrammus | 167110 |  | Benthic [5] |
| LCOD | Lingcod | Ophiodon elongatus | 167116 |  | Benthic |
| LOBS | California spiny lobster | Panulirus interruptus | 97650 |  | Benthic [11] |
| LSPN | Longspine thornyhead | Sebastolobus altivelis | 166784 |  | Benthic [4] |
| MAKO | Shortfin mako | Isurus oxyrinchus | 159924 |  | Pelagic |
| MSQD | Market squid | Loligo opalescens | 82371 | Doryteuthis opalescens | Pelagic [12] |
| NANC | Northern anchovy | Engraulis mordax | 161828 |  | Pelagic [13] |
| PCOD | Pacific cod | Gadus macrocephalus | 164711 |  | Benthic |
| PDAB | Pacific sanddab | Citharichthys sordidus | 172716 |  | Benthic [7] |
| PHRG | Pacific herring | Clupea pallasii | 551209 | Clupea pallasii pallasii | Pelagic [14] |
| PINK | Pink salmon | Oncorhynchus gorbuscha | 161975 |  | Pelagic |
| PSDN | Pacific sardine | Sardinops sagax | 161729 |  | Pelagic |
| PSHP | Pacific pink shrimp | Pandalus jordani | 96970 |  | Benthic [15] |
| PTRL | Petrale sole | Eopsetta jordani | 172868 |  | Benthic [7] |
| PWHT | Hake (Pacific whiting) | Merluccius productus | 164792 |  | Pelagic (see S1 Appendix) |
| REX | Rex sole | Glyptocephalus zachirus | 172978 |  | Benthic [2] |
| RPRW | Ridgeback prawn | Sicyonia ingentis | 96038 |  | Benthic [16] |
| RURC | Red sea urchin | Strongylocentrotus franciscanus | 157971 | Mesocentrotus franciscanus | Benthic [17] |
| SABL | Sablefish | Anoplopoma fimbria | 167123 |  | Benthic |
| SHPD | California sheephead | Semicossyphus pulcher | 170744 |  | Benthic [18] |
| SOCK | Sockeye salmon | Oncorhynchus nerka | 161979 |  | Pelagic |
| SPRW | Spotted prawn | Pandalus platyceros | 96979 | Used Pacific pink shrimp risk value due to missing spatial distribution | Benthic [19] |
| SSPN | Shortspine thornyhead | Sebastolobus alascanus | 166783 |  | Benthic [20] |
| STLH | Steelhead | Oncorhynchus mykiss | 161989 |  | Pelagic |
| SWRD | Swordfish | Xiphias gladius | 172482 |  | Pelagic |
| TSRK | Common thresher shark | Alopias vulpinus | 159916 |  | Pelagic |
| VRML | Vermilion rockfish | Sebastes miniatus | 166729 |  | Benthic [4] |
| WBAS | White seabass | Atractoscion nobilis | 169387 |  | Benthic [5] |
| YTRK | Yellowtail rockfish | Sebastes flavidus | 166720 |  | Benthic [7] |
| Unspecified | | | | |  |
| BSRM (1) | Ghost shrimp | Neotrypaea californiensis | 552849 |  | Benthic [21] |
| BSRM (2) | Bay shrimp | Crangon franciscorum | 97114 | If beam trawl is used | Benthic [22] |
| RCRB | Yellow rock crab | Cancer anthonyi | 98680 |  | Benthic [23] |
| RCRB | Brown rock crab | Cancer antennarius | 98673 |  | Benthic [23] |
| RCRB | Red rock crab | Cancer productus | 98672 |  | Benthic [24] |
| SMLT (1) | Surf smelt | Hypomesus pretiosus | 162030 | Eureka, CA | Pelagic [25] |
| SMLT (1) | Night smelt | Spirinchus starksi | 162048 | Eureka, CA | Pelagic [13] |
| SMLT (2) | Jacksmelt | Atherinopsis californiensis | 166012 | Santa Cruz, CA | Pelagic [26] |
| UHAG | Pacific hagfish | Eptatretus stoutii | 622244 | WA and CA | Benthic [13] |
| USCU (1,2) | Giant red sea cucumber | Apostichopus californicus | 1078919 | Hand take North of Point Conception; other take | Benthic [8] |
| USCU (2) | Warty sea cucumber | Apostichopus parvimensis | 1078867 | Hand take South of Point Conception | Benthic [27] |
| USKT | Longnose skate | Raja rhina | 160851 | WA | Benthic [28] |
| USKT | Big Skate | Raja binoculata | 160848 | WA | Benthic [7] |
| SRFP (1,2) | Striped seaperch | Embiotoca lateralis | 169744 | San Francisco ports + South of Pt. Arguello to south SLO County | Benthic [5] |
| SRFP (1,2) | Black surfperch | Embiotoca jacksoni | 169745 | San Francisco ports + South of Pt. Arguello to south SLO County | Benthic [5] |
| SRFP (2,3) | Barred surfperch | Amphistichus argenteus | 169758 | South of Pt. Arguello to south SLO County + South of San Fran. | Benthic [13] |
| SRFP (2) | Rainbow surfperch | Hypsurus caryi | 169761 | South of Pt. Arguello to south SLO County | Benthic [13] |
| SRFP (2) | Rubberlip surfperch | Rhacochilus toxotes | 169755 | South of Pt. Arguello to south SLO County | Benthic [13] |

References

1. Riede K. Global register of migratory species: from global to regional scales: final report of the R&D-Projekt 808 05 081. Federal Agency for Nature Conservation; 2004.

2. Russian Academy of Sciences. Catalog of vertebrates of Kamchatka and adjacent waters. Kamchatsky Pechatny Dvor, Petropavlovsk-Kamchatsky, Russia; 2000.

3. Hart JL. Paciﬁc ﬁshes of Canada. Bull Fish Res Board Can. 1973;180: 740.

4. Kramer DE, O’Connell VM. Guide to Northeast Pacific rockfishes. Genera Sebastes and Sebastolobus. Alaska Sea Grant, Mar Advis Bull No 25. 1995.

5. Eschmeyer WN, Herald ES, Hammann H. A field guide to Pacific coast fishes of North America. Boston, MA, USA: Houghton Mifflin Company; 1983.

6. Hiebert TC. Clinocardium nuttallii. 3rd Editio. In: Hiebert TC, Butler BA, Shanks AL, editors. Oregon Estuarine Invertebrates: Rudy’s Illustrated Guide to Common Species. 3rd Editio. Charleston, OR: University of Oregon Libraries and Oregon Institute of Marine Biology; 2015.

7. Allen MJ, Smith GB. Atlas and zoogeography of common fishes in the Bering Sea and northeastern Pacific. NOAA Tech Rep. 1988;NMFS 66: 151.

8. Gotshall DW. Guide to marine invertebrates: Alaska to Baja California (2d ed.) (revised). Sea Challengers. 2005; 117.

9. Harbo RM. Shells and shellfish of the Pacific Northwest, a field guide. Harbour Publishing, Canada; 1997.

10. Goodwin CL, Pease B. Species Profiles. Life Histories and Environmental Requirements of Coastal Fishes and Invertebrates (Pacific Northwest)-- Pacific Geoduck Clam. US Fish Wildl Serv Biol Rep. 1989;82: U.S. Army Corps of Engineers, TR EL82-4. 14p.

11. Neilson DJ. Assessment of the California spiny lobster (Panulirus interruptus). Calif Dep Fish Game. 2011; 138.

12. Roper CFE, Sweeney MJ, Nauen C. 1984 FAO Species Catalogue. Vol. 3. Cephalopods of the world. An annotated and illustrated catalogue of species of interest to fisheries. FAO Fish Synop. 1984;125: 277.

13. Love MS, Mecklenburg CW, Mecklenburg TA, Thorsteinson LK. Resource inventory of marine and estuarine fishes of the West Coast and Alaska: A checklist of North Pacific and Arctic Ocean species from Baja California to the Alaska-Yukon border. Seattle, WA: US Department of the Interior, US Geological Survey, Biological Resources Division; 2005.

14. Coad BW, Reist JD. Annotated list of the Arctic marine fishes of Canada. Can MS Rep Fish Aquat Sci. 2004;2674: iv+112p.

15. Dahlstrom WA. Synopsis of biological data on the ocean shrimp, Pandalus jordani, Rathbun, 1902. FAO Fisheries Synopsis No99 FAO Fisheries Report. 57(4):1377-1416. In: Mistakidis MN, editor. Proceedings of the World Scientific Conference on the Biology and Culture of Shrimps and Prawns, Mexico City, 12-21 June, 1967. 1970. pp. 1377–1416.

16. Sunada JS. Spot Prawn (Pandalus Platyceros) and Ridgeback prawn (Sicyonia ingentis) fisheries in the Santa Barbara Channel. CalCOFI Rep. 1984;25: 100–104.

17. Lambert P, Austin WC. Brittle stars, sea urchins and feather stars of British Columbia, Southeast Alaska and Puget Sound. Royal British Columbia Museum, Canada; 2007.

18. Gomon MF. Labridae. Viejas, doncellasas, señoritas. In: Fischer W, Krupp F, Schneider W, Sommer C, Carpenter KE, Niem V, editors. Guia FAO para Identification de Especies para lo Fines de la Pesca Pacifico Centro-Oriental 3 Vols. FAO Rome; 1995. pp. 1201–1225.

19. Komai T. A revision of the genus Pandalus (Crustacea: Decapoda: Caridea: Pandalidae). J Nat Hist. 1999;33: 1265–1372.

20. Fedorov V V, Chereshnev LA, Nazarkin M V, Shestakov A V, Volobuev V V. Catalog of Marine and Freshwater Fishes of the Northern Part of the Sea of Okhotsk. Vladivostok: Dal’nauka. 2003; 204 p.

21. OBIS. Neotrypaea californiensis (Dana, 1854). In: OBIS Search Interface. [Internet]. 2015. Available: http://www.iobis.org/mapper/

22. Carpenter KE, Niem VH. FAO species identification guide for fishery purposes. The living marine resources of the Western Central Pacific. Vol. 2. Cephalopods, crustaceans, holothurians and sharks. FAO Rome; 1998. pp. 687–1396.

23. Hines AH. Fecundity and reproductive output in nine species of Cancer crabs (Crustacea, Brachyura, Cancridae). Can J Fish Aquat Sci. 1991;48: 267–275.

24. Jensen GC. Pacific coast crabs and shrimps. Monterey, CA: Sea Challengers; 1995.

25. Froese R, Pauly D, editors. FishBase. World Wide Web electronic publication. (06/2021). 2021. Available: www.fishbase.org

26. De la Cruz Agüero J, Arellano Martínez M, Cota Gómez VM, De la Cruz-Agüero G. Catalogo de los peces marinos de Baja California Sur. La Paz, Mexico: IPN-CICIMAR; 1997.

27. Purcell SW, Samyn Y, Conand C. Commercially important sea cucumbers of the world. Rome, Italy: FAO; 2012.

28. Ormseth O, Matta B, Hoff J. Bering Sea and Aleutian Island skates [online]. 2008.
