# Supplementary material for: Social-ecological vulnerability of fishing communities to climate change: A U.S. West Coast case study
Source: PLoS One. 2022 Aug 17;17(8):e0272120. doi: 10.1371/journal.pone.0272120 (PMC9385011; doi:10.1371/journal.pone.0272120)
Supplement: S2 Table — Ecological exposure, sensitivity, and risk (Euclidean distance between exposure and sensitivity) of species to climate change, averaged across exposure and sensitivity to temperature, pH, oxygen, and chlorophyll, using three different climate models (GFDL, HAD, and IPSL), and average exposure, sensitivity, and risk across the three models for all species (in the top 90% of landings for communities and species that were not removed due to missing data or other reason, see supplemental information). Ordered from most to least ecologically at risk (Risk rank). (DOCX) [file pone.0272120.s007.docx]

| Species Name | Scientific Name | GFDL Exp | GFDL Sens | GFDL Risk | IPSL Exp | IPSL Sens | IPSL Risk | HAD Exp | HAD Sens | HAD Risk | avg exp | avg sens | Avg. Risk | Risk rank |
| --- | --- | --- | --- | --- | --- | --- | --- | --- | --- | --- | --- | --- | --- | --- |
| chum salmon | Oncorhynchus keta | 0.500 | 0.589 | 0.505 | 0.422 | 0.576 | 0.430 | 0.448 | 0.600 | 0.432 | 0.46 | 0.59 | 0.456 | 1 |
| night smelt | Spirinchus starksi | 0.538 | 0.593 | 0.532 | 0.419 | 0.561 | 0.414 | 0.413 | 0.595 | 0.418 | 0.46 | 0.58 | 0.455 | 2 |
| Pacific hake | Merluccius productus | 0.551 | 0.396 | 0.415 | 0.705 | 0.371 | 0.460 | 0.640 | 0.385 | 0.384 | 0.63 | 0.38 | 0.420 | 3 |
| surf smelt | Hypomesus pretiosus | 0.495 | 0.561 | 0.479 | 0.394 | 0.528 | 0.375 | 0.392 | 0.567 | 0.385 | 0.43 | 0.55 | 0.413 | 4 |
| chinook salmon | Oncorhynchus tshawytscha | 0.511 | 0.540 | 0.475 | 0.412 | 0.513 | 0.366 | 0.409 | 0.548 | 0.371 | 0.44 | 0.53 | 0.404 | 5 |
| sockeye salmon | Oncorhynchus nerka | 0.390 | 0.557 | 0.418 | 0.380 | 0.548 | 0.392 | 0.413 | 0.567 | 0.390 | 0.39 | 0.56 | 0.400 | 6 |
| sablefish | Anoplopoma fimbria | 0.191 | 0.593 | 0.404 | 0.361 | 0.551 | 0.390 | 0.308 | 0.564 | 0.373 | 0.29 | 0.57 | 0.389 | 7 |
| pink salmon | Oncorhynchus gorbuscha | 0.490 | 0.525 | 0.449 | 0.387 | 0.497 | 0.343 | 0.387 | 0.529 | 0.348 | 0.42 | 0.52 | 0.380 | 8 |
| jacksmelt | Atherinopsis californiensis | 0.527 | 0.497 | 0.456 | 0.392 | 0.463 | 0.313 | 0.512 | 0.471 | 0.346 | 0.48 | 0.48 | 0.371 | 9 |
| pacific herring | Clupea pallasii pallasii | 0.499 | 0.507 | 0.443 | 0.387 | 0.486 | 0.333 | 0.407 | 0.510 | 0.334 | 0.43 | 0.50 | 0.370 | 10 |
| kelp greenling | Hexagrammos decagrammus | 0.643 | 0.319 | 0.470 | 0.601 | 0.285 | 0.331 | 0.587 | 0.311 | 0.304 | 0.61 | 0.31 | 0.368 | 11 |
| bluefin tuna | Thunnus orientalis | 0.624 | 0.279 | 0.442 | 0.695 | 0.272 | 0.417 | 0.510 | 0.269 | 0.216 | 0.61 | 0.27 | 0.358 | 12 |
| brown rock crab | Cancer antennarius | 0.622 | 0.324 | 0.451 | 0.591 | 0.295 | 0.326 | 0.571 | 0.329 | 0.297 | 0.59 | 0.32 | 0.358 | 13 |
| common thresher shark | Alopias vulpinus | 0.524 | 0.318 | 0.357 | 0.696 | 0.298 | 0.424 | 0.576 | 0.307 | 0.292 | 0.60 | 0.31 | 0.358 | 14 |
| albacore | Thunnus alalunga | 0.597 | 0.274 | 0.415 | 0.690 | 0.252 | 0.407 | 0.536 | 0.265 | 0.240 | 0.61 | 0.26 | 0.354 | 15 |
| california spiny lobster | Panulirus interruptus | 0.455 | 0.485 | 0.396 | 0.523 | 0.468 | 0.379 | 0.368 | 0.454 | 0.270 | 0.45 | 0.47 | 0.349 | 16 |
| shortfin mako | Isurus oxyrinchus | 0.545 | 0.290 | 0.367 | 0.681 | 0.270 | 0.403 | 0.559 | 0.282 | 0.267 | 0.59 | 0.28 | 0.346 | 17 |
| red sea urchin | Mesocentrotus franciscanus | 0.679 | 0.189 | 0.487 | 0.581 | 0.167 | 0.289 | 0.568 | 0.191 | 0.260 | 0.61 | 0.18 | 0.346 | 18 |
| bigeye tuna | Thunnus obesus | 0.575 | 0.272 | 0.392 | 0.686 | 0.249 | 0.403 | 0.535 | 0.263 | 0.238 | 0.60 | 0.26 | 0.344 | 19 |
| swordfish | Xiphias gladius | 0.549 | 0.266 | 0.365 | 0.677 | 0.247 | 0.393 | 0.553 | 0.259 | 0.254 | 0.59 | 0.26 | 0.337 | 20 |
| black-and-yellow | Sebastes chrysomelas | 0.500 | 0.354 | 0.350 | 0.648 | 0.328 | 0.391 | 0.479 | 0.319 | 0.214 | 0.54 | 0.33 | 0.318 | 21 |
| longspine thornyhead | Sebastolobus altivelis | 0.264 | 0.513 | 0.332 | 0.359 | 0.468 | 0.308 | 0.374 | 0.491 | 0.308 | 0.33 | 0.49 | 0.316 | 22 |
| chub mackerel | Scomber japonicus | 0.530 | 0.432 | 0.416 | 0.362 | 0.407 | 0.250 | 0.446 | 0.421 | 0.268 | 0.45 | 0.42 | 0.312 | 23 |
| gopher rockfish | Sebastes carnatus | 0.521 | 0.337 | 0.361 | 0.599 | 0.309 | 0.338 | 0.505 | 0.304 | 0.227 | 0.54 | 0.32 | 0.309 | 24 |
| northern anchovy | Engraulis mordax | 0.520 | 0.426 | 0.405 | 0.371 | 0.401 | 0.247 | 0.458 | 0.415 | 0.270 | 0.45 | 0.41 | 0.307 | 25 |
| bay shrimp | Crangon franciscorum | 0.574 | 0.257 | 0.389 | 0.616 | 0.230 | 0.331 | 0.495 | 0.266 | 0.201 | 0.56 | 0.25 | 0.307 | 26 |
| pacific sardine | Sardinops sagax | 0.518 | 0.430 | 0.405 | 0.352 | 0.410 | 0.250 | 0.431 | 0.419 | 0.259 | 0.43 | 0.42 | 0.305 | 27 |
| california sheephead | Semicossyphus pulcher | 0.408 | 0.437 | 0.329 | 0.546 | 0.423 | 0.361 | 0.362 | 0.398 | 0.214 | 0.44 | 0.42 | 0.301 | 28 |
| ridgeback prawn | Sicyonia ingentis | 0.455 | 0.407 | 0.342 | 0.579 | 0.392 | 0.365 | 0.372 | 0.377 | 0.197 | 0.47 | 0.39 | 0.301 | 29 |
| california halibut | Paralichthys californicus | 0.548 | 0.320 | 0.379 | 0.560 | 0.290 | 0.295 | 0.503 | 0.300 | 0.224 | 0.54 | 0.30 | 0.299 | 30 |
| white seabass | Atractoscion nobilis | 0.559 | 0.309 | 0.387 | 0.572 | 0.280 | 0.303 | 0.494 | 0.281 | 0.207 | 0.54 | 0.29 | 0.299 | 31 |
| olive rockfish | Sebastes serranoides | 0.477 | 0.358 | 0.332 | 0.600 | 0.332 | 0.350 | 0.463 | 0.328 | 0.207 | 0.51 | 0.34 | 0.296 | 32 |
| warty sea cucumber | Parastichopus parvimensis | 0.464 | 0.345 | 0.314 | 0.621 | 0.318 | 0.362 | 0.480 | 0.312 | 0.210 | 0.52 | 0.33 | 0.295 | 33 |
| gaper clam | Tresus capax | 0.517 | 0.260 | 0.333 | 0.642 | 0.239 | 0.358 | 0.482 | 0.244 | 0.181 | 0.55 | 0.25 | 0.291 | 34 |
| yellow rock crab | Cancer anthonyi | 0.509 | 0.292 | 0.334 | 0.616 | 0.268 | 0.340 | 0.491 | 0.263 | 0.196 | 0.54 | 0.27 | 0.290 | 35 |
| pacific cod | Gadus macrocephalus | 0.369 | 0.419 | 0.290 | 0.455 | 0.377 | 0.266 | 0.515 | 0.408 | 0.300 | 0.45 | 0.40 | 0.286 | 36 |
| rainbow surfperch | Hypsurus caryi | 0.419 | 0.365 | 0.287 | 0.622 | 0.341 | 0.373 | 0.441 | 0.330 | 0.193 | 0.49 | 0.35 | 0.284 | 37 |
| blackgill rockfish | Sebastes melanostomus | 0.533 | 0.345 | 0.375 | 0.542 | 0.315 | 0.290 | 0.440 | 0.320 | 0.185 | 0.51 | 0.33 | 0.284 | 38 |
| giant red sea cucumber | Apostichopus californicus | 0.527 | 0.270 | 0.345 | 0.596 | 0.247 | 0.315 | 0.487 | 0.251 | 0.189 | 0.54 | 0.26 | 0.283 | 39 |
| black surfperch | Embiotoca jacksoni | 0.431 | 0.349 | 0.288 | 0.617 | 0.333 | 0.365 | 0.433 | 0.323 | 0.181 | 0.49 | 0.33 | 0.278 | 40 |
| rubberlip surfperch | Rhacochilus toxotes | 0.414 | 0.358 | 0.279 | 0.621 | 0.342 | 0.373 | 0.424 | 0.332 | 0.182 | 0.49 | 0.34 | 0.278 | 40 |
| brown rockfish | Sebastes auriculatus | 0.522 | 0.280 | 0.343 | 0.571 | 0.253 | 0.292 | 0.483 | 0.256 | 0.187 | 0.53 | 0.26 | 0.274 | 42 |
| coho salmon | Oncorhynchus kisutch | 0.455 | 0.421 | 0.350 | 0.321 | 0.392 | 0.227 | 0.405 | 0.412 | 0.241 | 0.39 | 0.41 | 0.273 | 43 |
| arrowtooth flounder | Atheresthes stomias | 0.437 | 0.334 | 0.285 | 0.468 | 0.298 | 0.220 | 0.587 | 0.325 | 0.309 | 0.50 | 0.32 | 0.271 | 44 |
| geoduck | Panopea abrupta | 0.518 | 0.253 | 0.333 | 0.571 | 0.224 | 0.285 | 0.494 | 0.229 | 0.190 | 0.53 | 0.24 | 0.269 | 45 |
| ghost shrimp | Neotrypaea californiensis | 0.509 | 0.264 | 0.326 | 0.568 | 0.234 | 0.284 | 0.487 | 0.247 | 0.187 | 0.52 | 0.25 | 0.266 | 46 |
| striped seaperch | Embiotoca lateralis | 0.511 | 0.256 | 0.326 | 0.589 | 0.235 | 0.305 | 0.462 | 0.241 | 0.162 | 0.52 | 0.24 | 0.264 | 47 |
| market squid | Doryteuthis opalescens | 0.407 | 0.417 | 0.314 | 0.297 | 0.397 | 0.231 | 0.365 | 0.423 | 0.238 | 0.36 | 0.41 | 0.261 | 48 |
| steelhead | Oncorhynchus mykiss | 0.418 | 0.415 | 0.320 | 0.292 | 0.395 | 0.228 | 0.350 | 0.421 | 0.234 | 0.35 | 0.41 | 0.261 | 49 |
| chilipepper rockfish | Sebastes goodei | 0.478 | 0.306 | 0.310 | 0.527 | 0.275 | 0.259 | 0.498 | 0.283 | 0.211 | 0.50 | 0.29 | 0.260 | 50 |
| barred surfperch | Amphistichus argenteus | 0.395 | 0.374 | 0.275 | 0.571 | 0.348 | 0.333 | 0.359 | 0.330 | 0.148 | 0.44 | 0.35 | 0.252 | 51 |
| vermillion | Sebastes miniatus | 0.482 | 0.290 | 0.308 | 0.519 | 0.262 | 0.247 | 0.485 | 0.282 | 0.199 | 0.50 | 0.28 | 0.251 | 52 |
| cabezon | Scorpaenichthys marmoratus | 0.471 | 0.278 | 0.294 | 0.522 | 0.250 | 0.245 | 0.508 | 0.253 | 0.209 | 0.50 | 0.26 | 0.249 | 53 |
| lingcod | Ophiodon elongatus | 0.473 | 0.269 | 0.293 | 0.503 | 0.237 | 0.222 | 0.501 | 0.251 | 0.202 | 0.49 | 0.25 | 0.239 | 54 |
| black rockfish | Sebastes melanops | 0.448 | 0.291 | 0.276 | 0.508 | 0.260 | 0.236 | 0.497 | 0.268 | 0.204 | 0.48 | 0.27 | 0.238 | 55 |
| pacific sanddab | Citharichthys sordidus | 0.458 | 0.288 | 0.284 | 0.497 | 0.260 | 0.226 | 0.493 | 0.274 | 0.203 | 0.48 | 0.27 | 0.238 | 56 |
| red rock crab | Cancer productus | 0.523 | 0.197 | 0.332 | 0.542 | 0.170 | 0.250 | 0.437 | 0.194 | 0.129 | 0.50 | 0.19 | 0.237 | 57 |
| shortspine thornyhead | Sebastolobus alascanus | 0.328 | 0.402 | 0.253 | 0.401 | 0.372 | 0.232 | 0.411 | 0.391 | 0.224 | 0.38 | 0.39 | 0.237 | 58 |
| dungeness crab | Metacarcinus magister | 0.485 | 0.225 | 0.296 | 0.501 | 0.200 | 0.211 | 0.500 | 0.227 | 0.195 | 0.50 | 0.22 | 0.234 | 59 |
| pacific pink shrimp | Pandalus jordani | 0.465 | 0.251 | 0.281 | 0.485 | 0.221 | 0.200 | 0.497 | 0.233 | 0.193 | 0.48 | 0.23 | 0.225 | 60 |
| basket cockle | Clinocardium nuttallii | 0.469 | 0.226 | 0.280 | 0.490 | 0.196 | 0.200 | 0.492 | 0.218 | 0.186 | 0.48 | 0.21 | 0.222 | 61 |
| rex sole | Glyptocephalus zachirus | 0.393 | 0.364 | 0.267 | 0.444 | 0.328 | 0.222 | 0.399 | 0.342 | 0.176 | 0.41 | 0.34 | 0.222 | 62 |
| yellowtail rockfish | Sebastes flavidus | 0.464 | 0.256 | 0.280 | 0.481 | 0.223 | 0.197 | 0.486 | 0.245 | 0.186 | 0.48 | 0.24 | 0.221 | 63 |
| dover sole | Microstomus pacificus | 0.363 | 0.372 | 0.250 | 0.395 | 0.338 | 0.200 | 0.408 | 0.371 | 0.206 | 0.39 | 0.36 | 0.219 | 64 |
| big skate | Beringraja binoculata | 0.459 | 0.255 | 0.275 | 0.479 | 0.222 | 0.195 | 0.486 | 0.244 | 0.185 | 0.47 | 0.24 | 0.219 | 65 |
| english sole | Parophrys vetulus | 0.453 | 0.259 | 0.271 | 0.475 | 0.227 | 0.193 | 0.483 | 0.248 | 0.184 | 0.47 | 0.24 | 0.216 | 66 |
| pacific hagfish | Eptatretus stoutii | 0.409 | 0.297 | 0.242 | 0.490 | 0.267 | 0.222 | 0.459 | 0.281 | 0.175 | 0.45 | 0.28 | 0.213 | 67 |
| longnose skate | Beringraja rhina | 0.410 | 0.277 | 0.235 | 0.484 | 0.257 | 0.212 | 0.483 | 0.266 | 0.190 | 0.46 | 0.27 | 0.213 | 68 |
| petrale sole | Eopsetta jordani | 0.453 | 0.248 | 0.268 | 0.472 | 0.225 | 0.189 | 0.467 | 0.239 | 0.166 | 0.46 | 0.24 | 0.208 | 69 |
