# Supplementary material for: Social-ecological vulnerability of fishing communities to climate change: A U.S. West Coast case study
Source: PLoS One. 2022 Aug 17;17(8):e0272120. doi: 10.1371/journal.pone.0272120 (PMC9385011; doi:10.1371/journal.pone.0272120)
Supplement: S3 Table — Ecological exposure, sensitivity, and risk of species to climate change, averaged across exposure and sensitivity to temperature, pH, oxygen, and chlorophyll, using three different climate models (GFDL, HAD, and IPSL) for a subset of species in the California Current including species caught by Indigenous populations. Average risk is the average across the three models. (DOCX) [file pone.0272120.s008.docx]

| Scientific Name | Common Name | GFDL Exp | GFDL Sens | GFDL Risk | IPSL Exp | IPSL Sens | IPSL Risk | HAD Exp | HAD Sens | HAD Risk | Average Risk |
| --- | --- | --- | --- | --- | --- | --- | --- | --- | --- | --- | --- |
| Thunnus alalunga | albacore | 0.5972 | 0.2743 | 0.4135 | 0.6904 | 0.2524 | 0.4054 | 0.5361 | 0.2648 | 0.2378 | 0.3522 |
| Sebastes melanops | black rockfish | 0.4477 | 0.2955 | 0.2754 | 0.5084 | 0.2688 | 0.2349 | 0.4966 | 0.2732 | 0.2033 | 0.2379 |
| Sebastes melanostomus | blackgill rockfish | 0.5331 | 0.3482 | 0.3744 | 0.5419 | 0.3218 | 0.2886 | 0.4404 | 0.3244 | 0.1837 | 0.2823 |
| Sebastes auriculatus | brown rockfish | 0.5218 | 0.2846 | 0.3424 | 0.5712 | 0.2620 | 0.2917 | 0.4833 | 0.2615 | 0.1867 | 0.2736 |
| Thunnus orientalis | bluefin tuna | 0.6240 | 0.2786 | 0.4407 | 0.6953 | 0.2715 | 0.4142 | 0.5101 | 0.2688 | 0.2144 | 0.3564 |
| Sebastes chrysomelas | black-and-yellow rockfish | 0.5002 | 0.3583 | 0.3493 | 0.6484 | 0.3355 | 0.3899 | 0.4792 | 0.3239 | 0.2131 | 0.3174 |
| Scorpaenichthys marmoratus | cabezon | 0.4714 | 0.2825 | 0.2934 | 0.5224 | 0.2594 | 0.2446 | 0.5080 | 0.2584 | 0.2091 | 0.2490 |
| Paralichthys californicus | california halibut | 0.5475 | 0.3239 | 0.3788 | 0.5596 | 0.2989 | 0.2939 | 0.5033 | 0.3044 | 0.2228 | 0.2985 |
| Oncorhynchus tshawytscha | chinook salmon | 0.5109 | 0.5437 | 0.4730 | 0.4116 | 0.5195 | 0.3623 | 0.4085 | 0.5514 | 0.3684 | 0.4012 |
| Oncorhynchus keta | chum salmon | 0.5004 | 0.5925 | 0.5035 | 0.4215 | 0.5831 | 0.4258 | 0.4482 | 0.6035 | 0.4300 | 0.4531 |
| Clinocardium nuttallii | basket cockle | 0.4686 | 0.2312 | 0.2796 | 0.4900 | 0.2063 | 0.2001 | 0.4923 | 0.2241 | 0.1862 | 0.2220 |
| Sebastes goodei | chilipepper rockfish | 0.4782 | 0.3106 | 0.3092 | 0.5266 | 0.2838 | 0.2576 | 0.4982 | 0.2877 | 0.2106 | 0.2592 |
| Scomber japonicus | chub mackerel | 0.5298 | 0.4343 | 0.4145 | 0.3624 | 0.4114 | 0.2443 | 0.4456 | 0.4237 | 0.2652 | 0.3080 |
| Oncorhynchus kisutch | coho salmon | 0.4547 | 0.4230 | 0.3483 | 0.3205 | 0.3974 | 0.2217 | 0.4046 | 0.4145 | 0.2380 | 0.2693 |
| Metacarcinus magister | dungeness crab | 0.4851 | 0.2307 | 0.2959 | 0.5006 | 0.2098 | 0.2111 | 0.5002 | 0.2322 | 0.1953 | 0.2341 |
| Microstomus pacificus | dover sole | 0.3627 | 0.3755 | 0.2488 | 0.3949 | 0.3449 | 0.1966 | 0.4082 | 0.3750 | 0.2043 | 0.2166 |
| Parophrys vetulus | english sole | 0.4531 | 0.2638 | 0.2706 | 0.4751 | 0.2360 | 0.1923 | 0.4828 | 0.2532 | 0.1835 | 0.2155 |
| Thunnus obesus | bigeye tuna | 0.5745 | 0.2718 | 0.3908 | 0.6862 | 0.2500 | 0.4009 | 0.5350 | 0.2625 | 0.2362 | 0.3426 |
| Panopea abrupta | geoduck | 0.5179 | 0.2580 | 0.3325 | 0.5708 | 0.2335 | 0.2844 | 0.4941 | 0.2346 | 0.1898 | 0.2689 |
| Sebastes carnatus | gopher rockfish | 0.5205 | 0.3415 | 0.3603 | 0.5989 | 0.3168 | 0.3372 | 0.5052 | 0.3085 | 0.2264 | 0.3080 |
| Sebastes rastrelliger^1^ | grass rockfish | 0.5221 | 0.3542 | 0.3670 | 0.6414 | 0.3279 | 0.3805 | 0.5023 | 0.3155 | 0.2275 | 0.3250 |
| Hexagrammos decagrammus | kelp greenling | 0.6428 | 0.3236 | 0.4694 | 0.6008 | 0.2946 | 0.3303 | 0.5867 | 0.3165 | 0.3031 | 0.3676 |
| Ophiodon elongatus | lingcod | 0.4732 | 0.2734 | 0.2925 | 0.5030 | 0.2459 | 0.2219 | 0.5007 | 0.2563 | 0.2015 | 0.2387 |
| Panulirus interruptus | california spiny lobster | 0.4553 | 0.4892 | 0.3952 | 0.5227 | 0.4761 | 0.3773 | 0.3684 | 0.4591 | 0.2690 | 0.3472 |
| Sebastolobus altivelis | longspine thornyhead | 0.2644 | 0.5154 | 0.3286 | 0.3591 | 0.4729 | 0.3029 | 0.3742 | 0.4942 | 0.3045 | 0.3120 |
| Isurus oxyrinchus | shortfin mako | 0.5445 | 0.2904 | 0.3658 | 0.6814 | 0.2711 | 0.4005 | 0.5590 | 0.2821 | 0.2650 | 0.3438 |
| Doryteuthis opalescens | market squid | 0.4072 | 0.4199 | 0.3118 | 0.2970 | 0.4029 | 0.2255 | 0.3651 | 0.4252 | 0.2353 | 0.2575 |
| Gadus macrocephalus | pacific cod | 0.3690 | 0.4225 | 0.2886 | 0.4548 | 0.3844 | 0.2633 | 0.5150 | 0.4114 | 0.2980 | 0.2833 |
| Citharichthys sordidus | pacific sanddab | 0.4580 | 0.2924 | 0.2839 | 0.4974 | 0.2686 | 0.2247 | 0.4929 | 0.2788 | 0.2021 | 0.2369 |
| Clupea pallasii pallasii | pacific herring | 0.4990 | 0.5107 | 0.4407 | 0.3865 | 0.4919 | 0.3284 | 0.4069 | 0.5131 | 0.3313 | 0.3668 |
| Oncorhynchus gorbuscha | pink salmon | 0.4901 | 0.5283 | 0.4476 | 0.3873 | 0.5031 | 0.3393 | 0.3869 | 0.5329 | 0.3451 | 0.3773 |
| Sardinops sagax | pacific sardine | 0.5175 | 0.4324 | 0.4034 | 0.3523 | 0.4146 | 0.2447 | 0.4310 | 0.4211 | 0.2557 | 0.3013 |
| Pandalus jordani | pacific pink shrimp | 0.4650 | 0.2557 | 0.2803 | 0.4847 | 0.2300 | 0.1998 | 0.4968 | 0.2385 | 0.1932 | 0.2244 |
| Eopsetta jordani | petrale sole | 0.4528 | 0.2530 | 0.2678 | 0.4722 | 0.2342 | 0.1889 | 0.4670 | 0.2445 | 0.1659 | 0.2075 |
| Merluccius productus | hake | 0.5514 | 0.3960 | 0.4123 | 0.7045 | 0.3712 | 0.4558 | 0.6395 | 0.3846 | 0.3808 | 0.4163 |
| Sicyonia ingentis | ridgeback prawn | 0.4552 | 0.4113 | 0.3412 | 0.5786 | 0.4007 | 0.3633 | 0.3716 | 0.3815 | 0.1952 | 0.2999 |
| Mesocentrotus franciscanus | red sea urchin | 0.6788 | 0.1950 | 0.4874 | 0.5808 | 0.1775 | 0.2889 | 0.5682 | 0.1969 | 0.2601 | 0.3455 |
| Anoplopoma fimbria | sablefish | 0.1914 | 0.5941 | 0.3991 | 0.3612 | 0.5537 | 0.3825 | 0.3081 | 0.5655 | 0.3686 | 0.3834 |
| Semicossyphus pulcher | california sheephead | 0.4077 | 0.4411 | 0.3277 | 0.5457 | 0.4319 | 0.3593 | 0.3620 | 0.4032 | 0.2132 | 0.3001 |
| Oncorhynchus nerka | sockeye salmon | 0.3903 | 0.5597 | 0.4154 | 0.3803 | 0.5544 | 0.3871 | 0.4131 | 0.5706 | 0.3881 | 0.3969 |
| Sebastolobus alascanus | shortspine thornyhead | 0.3279 | 0.4057 | 0.2511 | 0.4013 | 0.3785 | 0.2289 | 0.4106 | 0.3941 | 0.2223 | 0.2341 |
| Oncorhynchus mykiss | steelhead | 0.4183 | 0.4182 | 0.3183 | 0.2919 | 0.4005 | 0.2230 | 0.3501 | 0.4240 | 0.2310 | 0.2574 |
| Xiphias gladius | swordfish | 0.5487 | 0.2657 | 0.3642 | 0.6767 | 0.2473 | 0.3911 | 0.5528 | 0.2585 | 0.2523 | 0.3359 |
| Alopias vulpinus | common thresher shark | 0.5243 | 0.3175 | 0.3547 | 0.6955 | 0.2983 | 0.4213 | 0.5759 | 0.3067 | 0.2894 | 0.3552 |
| Sebastes miniatus | vermillion | 0.4819 | 0.2950 | 0.3073 | 0.5194 | 0.2710 | 0.2459 | 0.4853 | 0.2865 | 0.1985 | 0.2506 |
| Atractoscion nobilis | white seabass | 0.5593 | 0.3134 | 0.3865 | 0.5723 | 0.2889 | 0.3017 | 0.4944 | 0.2858 | 0.2064 | 0.2982 |
| Sebastes flavidus | yellowtail rockfish | 0.4636 | 0.2609 | 0.2801 | 0.4808 | 0.2327 | 0.1968 | 0.4859 | 0.2499 | 0.1855 | 0.2208 |
| Neotrypaea californiensis | ghost shrimp | 0.5085 | 0.2689 | 0.3257 | 0.5676 | 0.2435 | 0.2834 | 0.4869 | 0.2525 | 0.1872 | 0.2654 |
| Crangon franciscorum | bay shrimp | 0.5740 | 0.2623 | 0.3885 | 0.6163 | 0.2411 | 0.3305 | 0.4948 | 0.2718 | 0.2011 | 0.3067 |
| Cancer anthonyi | yellow rock crab | 0.5092 | 0.2964 | 0.3336 | 0.6160 | 0.2766 | 0.3389 | 0.4906 | 0.2682 | 0.1959 | 0.2895 |
| Cancer antennarius | brown rock crab | 0.6219 | 0.3289 | 0.4509 | 0.5910 | 0.3049 | 0.3250 | 0.5714 | 0.3337 | 0.2967 | 0.3575 |
| Cancer productus | red rock crab | 0.5232 | 0.2021 | 0.3320 | 0.5417 | 0.1801 | 0.2498 | 0.4373 | 0.1994 | 0.1292 | 0.2370 |
| Hypomesus pretiosus | surf smelt | 0.4946 | 0.5639 | 0.4775 | 0.3937 | 0.5350 | 0.3717 | 0.3923 | 0.5703 | 0.3828 | 0.4107 |
| Spirinchus starksi | night smelt | 0.5383 | 0.5961 | 0.5303 | 0.4187 | 0.5682 | 0.4107 | 0.4133 | 0.5988 | 0.4155 | 0.4522 |
| Atherinopsis californiensis | jacksmelt | 0.5272 | 0.4987 | 0.4528 | 0.3918 | 0.4668 | 0.3061 | 0.5122 | 0.4724 | 0.3428 | 0.3672 |
| Eptatretus stoutii | pacific hagfish | 0.4085 | 0.3011 | 0.2417 | 0.4901 | 0.2753 | 0.2209 | 0.4585 | 0.2860 | 0.1747 | 0.2124 |
| Apostichopus californicus | giant red sea cucumber | 0.5269 | 0.2747 | 0.3449 | 0.5961 | 0.2559 | 0.3141 | 0.4871 | 0.2556 | 0.1884 | 0.2825 |
| Parastichopus parvimensis | warty sea cucumber | 0.4644 | 0.3493 | 0.3136 | 0.6207 | 0.3267 | 0.3611 | 0.4797 | 0.3165 | 0.2091 | 0.2946 |
| Beringraja rhina | longnose skate | 0.4096 | 0.2814 | 0.2347 | 0.4838 | 0.2658 | 0.2112 | 0.4830 | 0.2707 | 0.1899 | 0.2119 |
| Beringraja binoculata | big skate | 0.4585 | 0.2599 | 0.2749 | 0.4791 | 0.2318 | 0.1949 | 0.4859 | 0.2490 | 0.1852 | 0.2183 |
| Embiotoca lateralis | striped seaperch | 0.5106 | 0.2606 | 0.3260 | 0.5890 | 0.2444 | 0.3045 | 0.4623 | 0.2466 | 0.1620 | 0.2641 |
| Embiotoca jacksoni | black surfperch | 0.4310 | 0.3530 | 0.2870 | 0.6171 | 0.3405 | 0.3637 | 0.4327 | 0.3273 | 0.1803 | 0.2770 |
| Amphistichus argenteus | barred surfperch | 0.3953 | 0.3774 | 0.2736 | 0.5712 | 0.3557 | 0.3313 | 0.3591 | 0.3342 | 0.1464 | 0.2505 |
| Hypsurus caryi | rainbow surfperch | 0.4185 | 0.3692 | 0.2863 | 0.6219 | 0.3487 | 0.3717 | 0.4414 | 0.3348 | 0.1918 | 0.2833 |
| Rhacochilus toxotes | rubberlip surfperch | 0.4140 | 0.3623 | 0.2785 | 0.6207 | 0.3501 | 0.3713 | 0.4240 | 0.3361 | 0.1811 | 0.2770 |
| Atheresthes stomias | arrowtooth flounder | 0.4373 | 0.3380 | 0.2844 | 0.4680 | 0.3064 | 0.2182 | 0.5872 | 0.3292 | 0.3088 | 0.2705 |
| Sebastes mystinus^2^ | blue rockfish | 0.4507 | 0.3154 | 0.2859 | 0.5036 | 0.2902 | 0.2398 | 0.4921 | 0.3003 | 0.2110 | 0.2456 |
| Tresus capax | gaper clam | 0.5171 | 0.2646 | 0.3331 | 0.6421 | 0.2484 | 0.3573 | 0.4815 | 0.2491 | 0.1810 | 0.2905 |
| Engraulis mordax | northern anchovy | 0.5202 | 0.4287 | 0.4034 | 0.3710 | 0.4060 | 0.2418 | 0.4582 | 0.4173 | 0.2667 | 0.3039 |
| Sebastes serranoides^3^ | olive rockfish | 0.4771 | 0.3624 | 0.3311 | 0.5998 | 0.3402 | 0.3482 | 0.4633 | 0.3321 | 0.2058 | 0.2951 |
| Glyptocephalus zachirus | rex sole | 0.3930 | 0.3679 | 0.2656 | 0.4439 | 0.3355 | 0.2192 | 0.3989 | 0.3464 | 0.1748 | 0.2199 |
| Mytilus trossulus^4^ | pacific blue mussels | 0.4915 | 0.2764 | 0.3110 | 0.6255 | 0.2537 | 0.3422 | 0.4731 | 0.2577 | 0.1758 | 0.2763 |
| Mytilus californianus^5^ | california mussels | 0.5101 | 0.2568 | 0.3246 | 0.5914 | 0.2410 | 0.3061 | 0.4864 | 0.2437 | 0.1844 | 0.2717 |
| Siliqua patula^5^ | pacific razor clams | 0.5492 | 0.2602 | 0.3638 | 0.5829 | 0.2390 | 0.2974 | 0.4767 | 0.2615 | 0.1805 | 0.2806 |
| Pollicipes polymerus^6^ | gooseneck (leaf) barnacle | 0.4981 | 0.2792 | 0.3181 | 0.6325 | 0.2635 | 0.3513 | 0.4843 | 0.2566 | 0.1860 | 0.2851 |
| Enteroctopus dofleini^7^ | north pacific giant octopus | 0.3611 | 0.3146 | 0.2077 | 0.4567 | 0.2884 | 0.1986 | 0.4702 | 0.3043 | 0.1944 | 0.2002 |
| Katharina tunicata^8^ | black katy chiton | 0.5089 | 0.2510 | 0.3224 | 0.5357 | 0.2322 | 0.2499 | 0.4334 | 0.2452 | 0.1343 | 0.2355 |
| Strongylocentrotus purpuratus^9^ | purple urchin | 0.5262 | 0.2947 | 0.3494 | 0.5603 | 0.2686 | 0.2834 | 0.5064 | 0.2774 | 0.2140 | 0.2823 |
| Saxidomus gigantea^10^ | washington butterclam | 0.5978 | 0.2463 | 0.4097 | 0.5971 | 0.2200 | 0.3082 | 0.5125 | 0.2490 | 0.2110 | 0.3096 |
| Leukoma staminea^5^ | pacific littleneck clam | 0.4921 | 0.2705 | 0.3100 | 0.6320 | 0.2499 | 0.3477 | 0.4867 | 0.2511 | 0.1866 | 0.2815 |

^1^Benthic/reef-associated [1]

^2^ Benthic/reef-associated [2]

^3^ Benthic/reef-associated [3]

^4^Benthic [4]

^5^ Benthic [5]

^6^Assumed benthic

^7^ Benthic [6]

^8^ Benthic [7]

^9^Benthic [8]

^10^ Benthic; **SAUP Database** 2006 SAUP Database. www.seaaroundus.org.

1. Moser HG. Scorpaenidae: scorpionfishes and rockfishes. In: Moser HG, editor. The early stages of fishes in the California Current region. CalCOFI Atlas No. 33.; 1996. pp. 733–795.

2. Hart JL. Paciﬁc ﬁshes of Canada. Bull Fish Res Board Can. 1973;180: 740.

3. Eschmeyer WN, Herald ES, Hammann H. A field guide to Pacific coast fishes of North America. Boston, MA, USA: Houghton Mifflin Company; 1983.

4. Palomares MLD, Pauly D, editors. SeaLifeBase. World Wide Web electronic publication. (08/2021). 2021. Available: www.sealifebase.org

5. Harbo RM. Shells and shellfish of the Pacific Northwest, a field guide. Harbour Publishing, Canada; 1997.

6. Jereb P, Roper CFE, Norman MD, Finn JK. Cephalopods of the world. An Annotated and Illustrated catalogue of Cephalopod species known to date. Vol. 3. Octopods and vampire squids. FAO Species Cat Fish Purp. 2014;3: 370p.

7. Dickson R. Chitons of British Columbia. In: Royal British Columbia Museum [Internet]. 2002. Available: http://www.royalbcmuseum.bc.ca/nh_papers/chitonkey/intro.htm

8. Lambert P, Austin WC. Brittle stars, sea urchins and feather stars of British Columbia, Southeast Alaska and Puget Sound. Royal British Columbia Museum, Canada; 2007.
