# Supplementary material for: Social-ecological vulnerability of fishing communities to climate change: A U.S. West Coast case study
Source: PLoS One. 2022 Aug 17;17(8):e0272120. doi: 10.1371/journal.pone.0272120 (PMC9385011; doi:10.1371/journal.pone.0272120)
Supplement: S4 Table — Average exposure and sensitivity (across the three climate models, HAD, IPSL, GFDL) to each climate variable (temperature, pH, chlorophyll, and oxygen) for each species in the risk assessment. Species are order from most at risk (overall) to least. (DOCX) [file pone.0272120.s009.docx]

| Species name | Avg. Temp exp | Avg. Temp sens | Avg Chl exp | Avg Chl sens | Avg Oxy exp | Avg Oxy sens | Avg pH exp | Avg pH sens |
| --- | --- | --- | --- | --- | --- | --- | --- | --- |
| Chum salmon | 0.9187 | 1.0000 | 0.3831 | 0.2408 | 0.2244 | 0.7547 | 0.3006 | 0.3575 |
| Night smelt | 0.8907 | 0.8702 | 0.3077 | 0.2722 | 0.2367 | 0.8196 | 0.3920 | 0.3697 |
| Hake | 0.8458 | 0.1497 | 0.5358 | 0.1480 | 0.1708 | 0.2580 | 0.9748 | 0.9791 |
| Surf smelt | 0.7634 | 0.7400 | 0.3455 | 0.2722 | 0.2051 | 0.7907 | 0.3936 | 0.4046 |
| Chinook salmon | 0.7557 | 0.7914 | 0.3544 | 0.2534 | 0.3080 | 0.7087 | 0.3565 | 0.3806 |
| sockeye salmon | 0.5903 | 0.8386 | 0.6552 | 0.2499 | 0.2695 | 0.7246 | 0.0631 | 0.4156 |
| sablefish | 0.1175 | 0.3427 | 0.5257 | 0.1960 | 0.4401 | 1.0000 | 0.0644 | 0.7377 |
| Pink salmon | 0.6382 | 0.6646 | 0.3694 | 0.2627 | 0.3040 | 0.7248 | 0.3742 | 0.4160 |
| Jacksmelt | 0.5018 | 0.0525 | 0.7415 | 0.3397 | 0.1080 | 0.8250 | 0.5570 | 0.6904 |
| Pacific herring | 0.5749 | 0.5531 | 0.4520 | 0.2753 | 0.2688 | 0.7478 | 0.4276 | 0.4277 |
| Kelp greenling | 0.8097 | 0.5743 | 0.3356 | 0.2751 | 0.4917 | 0.2354 | 0.8033 | 0.1355 |
| Bluefin tuna | 0.8356 | 0.0533 | 0.3097 | 0.0137 | 0.2939 | 0.0248 | 1.0000 | 1.0000 |
| Brown rock crab | 0.8349 | 0.6183 | 0.3049 | 0.2751 | 0.4596 | 0.2347 | 0.7796 | 0.1360 |
| Common thresher shark | 0.8333 | 0.0876 | 0.3950 | 0.0653 | 0.1892 | 0.0971 | 0.9768 | 0.9791 |
| Albacore | 0.8035 | 0.0463 | 0.3814 | 0.0137 | 0.2610 | 0.0152 | 0.9856 | 0.9791 |
| California spiny lobster | 0.3069 | 0.1282 | 0.6496 | 1.0000 | 0.5828 | 0.5078 | 0.2558 | 0.2403 |
| shortfin mako | 0.8102 | 0.0703 | 0.4090 | 0.0276 | 0.1850 | 0.0470 | 0.9758 | 0.9791 |
| Red sea urchin | 0.5767 | 0.3334 | 0.3333 | 0.2138 | 0.8390 | 0.1821 | 0.6880 | 0.0000 |
| Bigeye tuna | 0.7961 | 0.0486 | 0.3862 | 0.0069 | 0.2308 | 0.0103 | 0.9813 | 0.9791 |
| swordfish | 0.7819 | 0.0486 | 0.4261 | 0.0000 | 0.1888 | 0.0000 | 0.9742 | 0.9791 |
| Black-and-Yellow | 0.3573 | 0.2203 | 0.7212 | 0.3501 | 0.5408 | 0.4938 | 0.5511 | 0.2710 |
| Longspine thornyhead | 0.0792 | 0.2308 | 0.7097 | 0.2866 | 0.5400 | 0.9015 | 0.0013 | 0.5438 |
| Chub Mackerel | 0.3282 | 0.0298 | 0.6691 | 0.3067 | 0.1845 | 0.7719 | 0.6019 | 0.5711 |
| gopher rockfish | 0.3705 | 0.1844 | 0.7513 | 0.3364 | 0.5642 | 0.5052 | 0.4802 | 0.2403 |
| Northern anchovy | 0.3282 | 0.0040 | 0.6732 | 0.3067 | 0.1845 | 0.7745 | 0.6131 | 0.5711 |
| Bay shrimp | 0.8330 | 0.6009 | 0.2047 | 0.2788 | 0.4642 | 0.1158 | 0.7449 | 0.0083 |
| Pacific sardine | 0.3114 | 0.0344 | 0.6630 | 0.2967 | 0.2015 | 0.7757 | 0.5586 | 0.5711 |
| California sheephead | 0.2892 | 0.1568 | 0.6850 | 0.8374 | 0.5634 | 0.4730 | 0.2164 | 0.2106 |
| Ridgeback prawn | 0.3428 | 0.1375 | 0.6258 | 0.6705 | 0.5769 | 0.5204 | 0.3283 | 0.2403 |
| California Halibut | 0.3834 | 0.2093 | 0.7241 | 0.2999 | 0.6806 | 0.4832 | 0.3591 | 0.2206 |
| white seabass | 0.4183 | 0.2093 | 0.6958 | 0.2999 | 0.6646 | 0.4407 | 0.3893 | 0.2106 |
| Warty sea cucumber | 0.3218 | 0.2216 | 0.7212 | 0.3501 | 0.5515 | 0.4886 | 0.4918 | 0.2403 |
| Gaper clam | 0.4438 | 0.2850 | 0.6298 | 0.3299 | 0.5638 | 0.2574 | 0.5502 | 0.1173 |
| Yellow rock crab | 0.4149 | 0.2124 | 0.6784 | 0.3229 | 0.5949 | 0.3800 | 0.4662 | 0.1817 |
| Pacific cod | 0.3208 | 0.3476 | 0.4689 | 0.2289 | 0.4774 | 0.6485 | 0.5179 | 0.3806 |
| Rainbow surfperch | 0.2894 | 0.2112 | 0.6471 | 0.3816 | 0.5361 | 0.5180 | 0.5033 | 0.2710 |
| blackgill rockfish | 0.3064 | 0.0441 | 0.7535 | 0.2963 | 0.6570 | 0.6302 | 0.3036 | 0.3352 |
| Giant red sea cucumber | 0.4197 | 0.2476 | 0.6224 | 0.3130 | 0.6310 | 0.3176 | 0.4738 | 0.1444 |
| Rubberlip surfperch | 0.2951 | 0.1984 | 0.6463 | 0.4037 | 0.5373 | 0.5133 | 0.4663 | 0.2606 |
| Black surfperch | 0.3271 | 0.1844 | 0.6760 | 0.3965 | 0.5351 | 0.4975 | 0.4361 | 0.2606 |
| Brown rockfish | 0.4070 | 0.2748 | 0.6315 | 0.3098 | 0.6626 | 0.3405 | 0.4006 | 0.1263 |
| Coho salmon | 0.2596 | 0.0000 | 0.6376 | 0.2999 | 0.1457 | 0.8145 | 0.5302 | 0.5173 |
| Arrowtooth flounder | 0.4253 | 0.3707 | 0.4116 | 0.2260 | 0.5093 | 0.4573 | 0.6437 | 0.2206 |
| geoduck | 0.4543 | 0.2516 | 0.6052 | 0.2969 | 0.6305 | 0.2930 | 0.4205 | 0.0997 |
| Ghost shrimp | 0.4175 | 0.2675 | 0.6184 | 0.3067 | 0.6493 | 0.3102 | 0.3987 | 0.1086 |
| Striped seaperch | 0.4880 | 0.3370 | 0.5365 | 0.3299 | 0.5171 | 0.2171 | 0.5410 | 0.0909 |
| Market squid | 0.1564 | 0.0286 | 0.5885 | 0.3005 | 0.1584 | 0.8159 | 0.5224 | 0.5041 |
| steelhead | 0.1782 | 0.0217 | 0.5610 | 0.3005 | 0.1449 | 0.8156 | 0.5297 | 0.5041 |
| Chilipepper rockfish | 0.3770 | 0.1246 | 0.7093 | 0.3067 | 0.6335 | 0.5105 | 0.2841 | 0.2106 |
| Barred surfperch | 0.2936 | 0.1750 | 0.5593 | 0.4816 | 0.5531 | 0.4933 | 0.3615 | 0.2507 |
| vermillion | 0.3951 | 0.1793 | 0.6732 | 0.3098 | 0.6426 | 0.4499 | 0.2712 | 0.1728 |
| Cabezon | 0.3912 | 0.2241 | 0.6663 | 0.2999 | 0.6488 | 0.3906 | 0.2961 | 0.1263 |
| lingcod | 0.5091 | 0.1774 | 0.4520 | 0.2781 | 0.6083 | 0.3997 | 0.3999 | 0.1536 |
| Black Rockfish | 0.3904 | 0.1443 | 0.6474 | 0.3067 | 0.6166 | 0.4595 | 0.2825 | 0.1817 |
| pacific sanddab | 0.3642 | 0.0979 | 0.7212 | 0.3098 | 0.6162 | 0.4963 | 0.2294 | 0.1914 |
| Red rock crab | 0.5993 | 0.2588 | 0.2914 | 0.2846 | 0.5229 | 0.1707 | 0.5894 | 0.0322 |
| shortspine thornyhead | 0.1897 | 0.1351 | 0.7252 | 0.2896 | 0.5565 | 0.7471 | 0.0483 | 0.3806 |
| Dungeness crab | 0.5540 | 0.2648 | 0.3614 | 0.2722 | 0.6353 | 0.2829 | 0.4304 | 0.0487 |
| Pacific pink shrimp | 0.4482 | 0.1326 | 0.4878 | 0.2907 | 0.6260 | 0.3899 | 0.3667 | 0.1263 |
| basket cockle | 0.4689 | 0.2662 | 0.4047 | 0.2846 | 0.6615 | 0.2701 | 0.3994 | 0.0322 |
| Rex sole | 0.3946 | 0.1531 | 0.4154 | 0.2686 | 0.5255 | 0.6228 | 0.3121 | 0.3352 |
| yellowtail rockfish | 0.4353 | 0.1326 | 0.5092 | 0.2975 | 0.6162 | 0.3997 | 0.3464 | 0.1355 |
| Dover sole | 0.2777 | 0.1247 | 0.5816 | 0.2907 | 0.5278 | 0.6675 | 0.1674 | 0.3582 |
| Big Skate | 0.4353 | 0.1289 | 0.5092 | 0.2975 | 0.6064 | 0.3997 | 0.3471 | 0.1355 |
| English sole | 0.4299 | 0.1375 | 0.4934 | 0.2975 | 0.6163 | 0.4075 | 0.3418 | 0.1355 |
| Pacific hagfish | 0.3522 | 0.0734 | 0.6845 | 0.3031 | 0.5839 | 0.5292 | 0.1888 | 0.2206 |
| Longnose skate | 0.3755 | 0.0651 | 0.6711 | 0.3035 | 0.5706 | 0.4973 | 0.2181 | 0.2008 |
| Petrale sole | 0.4138 | 0.0991 | 0.4953 | 0.2943 | 0.6109 | 0.4121 | 0.3360 | 0.1444 |
