# Supplementary material for: Social-ecological vulnerability of fishing communities to climate change: A U.S. West Coast case study
Source: PLoS One. 2022 Aug 17;17(8):e0272120. doi: 10.1371/journal.pone.0272120 (PMC9385011; doi:10.1371/journal.pone.0272120)
Supplement: S5 Table — Fishing communities included in the analysis, pacFIN port group/name (https://pacfin.psmfc.org/pacfin_pub/data_rpts_pub/code_lists/pc_tree.txt) that each community is part of, axes of community vulnerability, and overall community vulnerability scores and rankings for each community. Community exposure is the average ecological risk (across climate models) weighted by percent revenue of species for each community. Adaptive capacity, based on social indicators, is calculated so that smaller values (closer to 0) equal greater adaptive capacity and greater values (close to 1) equal lower adaptive capacity. Risk is calculated as the Euclidean distance between exposure and sensitivity and vulnerability is the Euclidean distance between exposure, sensitivity, and adaptive capacity. Rank values are from most vulnerable, most at risk, or least adaptive, to least vulnerable, least at risk, or most adaptive, respectively. (DOCX) [file pone.0272120.s010.docx]

| Community | Port Code | Port Name | Sensitivity | Exposure | Adaptive capacity | Risk score | Vuln. | Adapt. rank | Risk rank | Vuln rank |
| --- | --- | --- | --- | --- | --- | --- | --- | --- | --- | --- |
| NEAH BAY, WA | NEA | Neah Bay | 0.957 | 0.957 | 0.915 | 1.354 | 1.634 | 23 | 2 | 1 |
| LONGVIEW, WA | OCR | Other Columbia River ports | 0.725 | 0.961 | 0.895 | 1.204 | 1.500 | 28 | 11 | 2 |
| NATIONAL CITY, CA | OSD | Other San Diego County ports | 0.636 | 0.868 | 1.000 | 1.076 | 1.469 | 1 | 27 | 3 |
| POINT ARENA, CA | ARE | Point Arena | 0.969 | 0.950 | 0.550 | 1.357 | 1.464 | 116 | 1 | 4 |
| IMPERIAL BEACH, CA | OSD | Other San Diego County ports | 0.686 | 0.868 | 0.938 | 1.107 | 1.451 | 16 | 19 | 5 |
| OXNARD, CA | OXN | Oxnard | 0.779 | 0.690 | 0.985 | 1.041 | 1.433 | 5 | 34 | 6 |
| FORT BRAGG, CA | BRG | Fort Bragg | 0.903 | 0.686 | 0.868 | 1.134 | 1.428 | 35 | 14 | 7 |
| EVERETT, WA | EVR | Everett | 0.725 | 1.000 | 0.713 | 1.235 | 1.426 | 75 | 8 | 8 |
| SANTA BARBARA, CA | SB | Santa Barbara | 0.810 | 0.946 | 0.651 | 1.245 | 1.405 | 91 | 6 | 9 |
| ALBION, CA | ALB | Albion | 0.930 | 0.950 | 0.411 | 1.329 | 1.391 | 152 | 3 | 10 |
| MOSS LANDING, CA | MOS | Moss landing | 1.000 | 0.376 | 0.888 | 1.068 | 1.389 | 30 | 28 | 11 |
| LA PUSH, WA | LAP | La Push | 0.977 | 0.643 | 0.721 | 1.170 | 1.374 | 72 | 13 | 12 |
| WESTPORT, WA | WPT | Westport | 0.992 | 0.651 | 0.674 | 1.187 | 1.365 | 85 | 12 | 13 |
| CATHLAMET, WA | OCR | Other Columbia River ports | 0.895 | 0.961 | 0.310 | 1.314 | 1.350 | 176 | 4 | 14 |
| KELSO, WA | OCR | Other Columbia River ports | 0.000 | 0.961 | 0.926 | 0.961 | 1.335 | 20 | 50 | 15 |
| CHULA VISTA, CA | OSD | Other San Diego County ports | 0.000 | 0.868 | 0.996 | 0.868 | 1.321 | 2 | 78 | 16 |
| CRESCENT CITY, CA | CRS | Crescent City | 0.892 | 0.054 | 0.973 | 0.893 | 1.321 | 8 | 74 | 17 |
| PORT HUENEME, CA | HNM | Port Hueneme | 0.845 | 0.341 | 0.954 | 0.911 | 1.319 | 13 | 67 | 18 |
| PUGET ISLAND, WA | OCR | Other Columbia River ports | 0.826 | 0.961 | 0.310 | 1.267 | 1.305 | 176 | 5 | 19 |
| LOS ANGELES, CA | OLA | Other LA and Orange County ports | 0.636 | 0.698 | 0.892 | 0.944 | 1.298 | 29 | 57 | 20 |
| FIELDS LANDING, CA | FLN | Fields Landing | 0.950 | 0.000 | 0.872 | 0.950 | 1.289 | 34 | 55 | 21 |
| EL CAJON, CA | OSD | Other San Diego County ports | 0.000 | 0.868 | 0.938 | 0.868 | 1.278 | 16 | 78 | 22 |
| BANDON, OR | BDN | Bandon | 0.864 | 0.550 | 0.752 | 1.025 | 1.271 | 65 | 41 | 23 |
| ILWACO, WA | LWC | Ilwaco/Chinook | 0.981 | 0.570 | 0.566 | 1.134 | 1.268 | 113 | 14 | 24 |
| KLAMATH, CA | ODN | Other Del Norte County ports | 0.830 | 0.058 | 0.950 | 0.832 | 1.262 | 14 | 98 | 25 |
| WASHOUGAL, WA | OCR | Other Columbia River ports | 0.779 | 0.961 | 0.202 | 1.237 | 1.254 | 207 | 7 | 26 |
| NEWPORT, OR | NEW | Newport | 0.934 | 0.562 | 0.597 | 1.090 | 1.243 | 104 | 25 | 27 |
| LEMON GROVE, CA | OSD | Other San Diego County ports | 0.000 | 0.868 | 0.884 | 0.868 | 1.239 | 31 | 78 | 28 |
| RICHMOND, CA | RCH | Richmond | 0.686 | 0.647 | 0.802 | 0.943 | 1.238 | 52 | 59 | 29 |
| TACOMA, WA | TAC | Tacoma | 0.686 | 0.640 | 0.806 | 0.938 | 1.237 | 51 | 61 | 30 |
| CHINOOK, WA | LWC | Ilwaco/Chinook | 0.973 | 0.570 | 0.504 | 1.127 | 1.235 | 129 | 17 | 31 |
| ESCONDIDO, CA | OSD | Other San Diego County ports | 0.000 | 0.868 | 0.876 | 0.868 | 1.233 | 33 | 78 | 32 |
| SAN SIMEON, CA | OSL | Other San Luis Obispo County ports | 0.911 | 0.585 | 0.589 | 1.083 | 1.233 | 107 | 26 | 33 |
| CAMAS, WA | OCR | Other Columbia River ports | 0.756 | 0.961 | 0.101 | 1.223 | 1.227 | 233 | 9 | 34 |
| RIDGEFIELD, WA | OCR | Other Columbia River ports | 0.756 | 0.961 | 0.050 | 1.223 | 1.224 | 246 | 9 | 35 |
| VISTA, CA | OSD | Other San Diego County ports | 0.000 | 0.868 | 0.861 | 0.868 | 1.222 | 37 | 78 | 36 |
| SAN DIEGO, CA | SD | San Diego | 0.686 | 0.682 | 0.740 | 0.968 | 1.218 | 68 | 49 | 37 |
| SOUTH GATE, CA | OLA | Other LA and Orange County ports | 0.000 | 0.698 | 0.992 | 0.698 | 1.213 | 3 | 109 | 38 |
| WINCHESTER BAY, OR | WIN | Winchester | 0.985 | 0.318 | 0.632 | 1.035 | 1.212 | 96 | 37 | 39 |
| SPRING VALLEY, CA | OSD | Other San Diego County ports | 0.000 | 0.868 | 0.845 | 0.868 | 1.212 | 41 | 78 | 40 |
| TILLAMOOK, OR | TLL | Tillamook/Garibaldi | 0.857 | 0.306 | 0.795 | 0.910 | 1.208 | 53 | 68 | 41 |
| LOMPOC, CA | OBV | Other Santa Barbara and Ventura County ports | 0.636 | 0.395 | 0.946 | 0.749 | 1.206 | 15 | 104 | 42 |
| ASTORIA, OR | AST | Astoria | 0.923 | 0.581 | 0.516 | 1.090 | 1.206 | 126 | 24 | 42 |
| BELL GARDENS, CA | OLA | Other LA and Orange County ports | 0.000 | 0.698 | 0.981 | 0.698 | 1.204 | 6 | 109 | 44 |
| VALLEY CENTER, CA | OSD | Other San Diego County ports | 0.725 | 0.868 | 0.407 | 1.131 | 1.202 | 154 | 16 | 45 |
| LYNWOOD, CA | OLA | Other LA and Orange County ports | 0.000 | 0.698 | 0.977 | 0.698 | 1.200 | 7 | 109 | 46 |
| EUREKA, CA | ERK | Eureka | 0.841 | 0.326 | 0.787 | 0.902 | 1.197 | 56 | 70 | 47 |
| PARAMOUNT, CA | OLA | Other LA and Orange County ports | 0.000 | 0.698 | 0.965 | 0.698 | 1.191 | 9 | 109 | 48 |
| GOLD BEACH, OR | GLD | Gold Beach | 0.880 | 0.659 | 0.457 | 1.099 | 1.191 | 141 | 21 | 49 |
| FALLBROOK, CA | OSD | Other San Diego County ports | 0.000 | 0.868 | 0.814 | 0.868 | 1.190 | 48 | 78 | 50 |
| VERNON, CA | OLA | Other LA and Orange County ports | 0.000 | 0.698 | 0.961 | 0.698 | 1.188 | 11 | 109 | 51 |
| COOS BAY, OR | COS | Charleston (Coos Bay) | 0.888 | 0.330 | 0.717 | 0.947 | 1.188 | 74 | 56 | 51 |
| MORRO BAY, CA | MRO | Morro Bay | 0.884 | 0.655 | 0.446 | 1.100 | 1.187 | 144 | 20 | 53 |
| SANTA ANA, CA | OLA | Other LA and Orange County ports | 0.000 | 0.698 | 0.957 | 0.698 | 1.185 | 12 | 109 | 54 |
| PORT ORFORD, OR | ORF | Port Orford | 0.946 | 0.558 | 0.419 | 1.098 | 1.175 | 150 | 22 | 55 |
| FLORENCE, OR | FLR | Florence | 0.779 | 0.663 | 0.570 | 1.023 | 1.171 | 112 | 42 | 56 |
| VANCOUVER, WA | OCR | Other Columbia River ports | 0.000 | 0.961 | 0.663 | 0.961 | 1.168 | 88 | 50 | 57 |
| BELLINGHAM, WA | BLL | Bellingham | 0.810 | 0.671 | 0.500 | 1.052 | 1.164 | 130 | 30 | 58 |
| INGLEWOOD, CA | OLA | Other LA and Orange County ports | 0.000 | 0.698 | 0.930 | 0.698 | 1.163 | 19 | 109 | 59 |
| GARDENA, CA | OLA | Other LA and Orange County ports | 0.000 | 0.698 | 0.923 | 0.698 | 1.157 | 21 | 109 | 60 |
| LITTLE RIVER, CA | OMD | Other Mendocino County ports | 0.965 | 0.442 | 0.454 | 1.062 | 1.154 | 142 | 29 | 61 |
| SAN MARCOS, CA | OSD | Other San Diego County ports | 0.000 | 0.868 | 0.760 | 0.868 | 1.154 | 63 | 78 | 62 |
| CARSON, CA | OLA | Other LA and Orange County ports | 0.000 | 0.698 | 0.919 | 0.698 | 1.154 | 22 | 109 | 63 |
| WILLITS, CA | OMD | Other Mendocino County ports | 0.636 | 0.442 | 0.849 | 0.774 | 1.149 | 40 | 103 | 64 |
| SHELTER COVE, CA | OHB | Other Humboldt County ports | 0.938 | 0.004 | 0.655 | 0.938 | 1.144 | 90 | 60 | 65 |
| AVALON, CA | OLA | Other LA and Orange County ports | 0.845 | 0.698 | 0.326 | 1.096 | 1.143 | 175 | 23 | 66 |
| SANTA CRUZ, CA | CRZ | Santa Cruz | 0.806 | 0.566 | 0.578 | 0.985 | 1.142 | 110 | 47 | 67 |
| LANCASTER, CA | OLA | Other LA and Orange County ports | 0.000 | 0.698 | 0.903 | 0.698 | 1.141 | 25 | 109 | 68 |
| ANAHEIM, CA | OLA | Other LA and Orange County ports | 0.000 | 0.698 | 0.899 | 0.698 | 1.138 | 27 | 109 | 69 |
| PORT ANGELES, WA | PAG | Port Angeles | 0.822 | 0.384 | 0.682 | 0.907 | 1.135 | 83 | 69 | 70 |
| SAN BUENAVENTURA (VENTURA), CA | VEN | Ventura | 0.818 | 0.341 | 0.702 | 0.886 | 1.130 | 78 | 76 | 71 |
| TOMALES, CA | TML | Tomales | 0.926 | 0.636 | 0.109 | 1.124 | 1.129 | 229 | 18 | 72 |
| NORWALK, CA | OLA | Other LA and Orange County ports | 0.000 | 0.698 | 0.880 | 0.698 | 1.123 | 32 | 109 | 73 |
| BONITA, CA | OSD | Other San Diego County ports | 0.000 | 0.868 | 0.709 | 0.868 | 1.121 | 76 | 78 | 74 |
| COVINA, CA | OLA | Other LA and Orange County ports | 0.000 | 0.698 | 0.864 | 0.698 | 1.111 | 36 | 109 | 75 |
| WESTMINSTER, CA | OLA | Other LA and Orange County ports | 0.000 | 0.698 | 0.857 | 0.698 | 1.105 | 38 | 109 | 76 |
| FULLERTON, CA | OLA | Other LA and Orange County ports | 0.000 | 0.698 | 0.853 | 0.698 | 1.102 | 39 | 109 | 77 |
| LA MESA, CA | OSD | Other San Diego County ports | 0.000 | 0.868 | 0.659 | 0.868 | 1.090 | 89 | 78 | 78 |
| ALHAMBRA, CA | OLA | Other LA and Orange County ports | 0.000 | 0.698 | 0.833 | 0.698 | 1.087 | 44 | 109 | 79 |
| CAYUCOS, CA | OSL | Other San Luis Obispo County ports | 0.798 | 0.585 | 0.442 | 0.990 | 1.084 | 145 | 46 | 80 |
| VALLEJO, CA | OSF | Other San Francisco Bay and San Mateo County ports | 0.686 | 0.058 | 0.837 | 0.689 | 1.084 | 43 | 148 | 81 |
| WALNUT, CA | OLA | Other LA and Orange County ports | 0.000 | 0.698 | 0.830 | 0.698 | 1.084 | 45 | 109 | 82 |
| GARIBALDI, OR | TLL | Tillamook/Garibaldi | 0.942 | 0.306 | 0.419 | 0.990 | 1.075 | 150 | 44 | 83 |
| UPLAND, CA | OLA | Other LA and Orange County ports | 0.000 | 0.698 | 0.814 | 0.698 | 1.072 | 48 | 109 | 84 |
| TRINIDAD, CA | TRN | Trinidad | 0.988 | 0.058 | 0.392 | 0.990 | 1.065 | 157 | 45 | 85 |
| SANTA PAULA, CA | OBV | Other Santa Barbara and Ventura County ports | 0.000 | 0.395 | 0.988 | 0.395 | 1.065 | 4 | 189 | 86 |
| UKIAH, CA | OMD | Other Mendocino County ports | 0.000 | 0.442 | 0.965 | 0.442 | 1.062 | 9 | 185 | 87 |
| LA CONNER, WA | LAC | La Conner | 0.961 | 0.388 | 0.221 | 1.036 | 1.060 | 202 | 36 | 88 |
| NEWPORT BEACH, CA | NWB | Newport Beach | 0.779 | 0.694 | 0.155 | 1.043 | 1.055 | 219 | 33 | 89 |
| BODEGA BAY, CA | BDG | Bodega Bay | 0.996 | 0.333 | 0.085 | 1.050 | 1.054 | 237 | 32 | 90 |
| DANA POINT, CA | DNA | Dana Point | 0.802 | 0.678 | 0.062 | 1.051 | 1.053 | 243 | 31 | 91 |
| BERKELEY, CA | BKL | Berkeley | 0.771 | 0.667 | 0.244 | 1.020 | 1.048 | 196 | 43 | 92 |
| HAWAIIAN GARDENS, CA | OLA | Other LA and Orange County ports | 0.000 | 0.698 | 0.775 | 0.698 | 1.043 | 59 | 109 | 93 |
| BROOKINGS, OR | BRK | Brookings | 0.876 | 0.298 | 0.481 | 0.925 | 1.043 | 135 | 63 | 94 |
| REDONDO BEACH, CA | OLA | Other LA and Orange County ports | 0.756 | 0.698 | 0.167 | 1.029 | 1.042 | 216 | 39 | 95 |
| HERMOSA BEACH, CA | OLA | Other LA and Orange County ports | 0.771 | 0.698 | 0.004 | 1.040 | 1.040 | 258 | 35 | 96 |
| GLENDALE, CA | OLA | Other LA and Orange County ports | 0.000 | 0.698 | 0.767 | 0.698 | 1.037 | 61 | 109 | 97 |
| MARIETTA-ALDERWOOD, WA | BLL | Bellingham | 0.000 | 0.671 | 0.791 | 0.671 | 1.037 | 55 | 152 | 98 |
| PACIFIC CITY, OR | PCC | Pacific City | 0.919 | 0.314 | 0.361 | 0.971 | 1.036 | 166 | 48 | 99 |
| ALAMEDA, CA | ALM | Alameda | 0.725 | 0.058 | 0.733 | 0.727 | 1.032 | 70 | 106 | 100 |
| DILLON BEACH, CA | OSM | Other Sonoma and Marine County Outer Coast ports | 0.915 | 0.465 | 0.109 | 1.026 | 1.032 | 229 | 40 | 101 |
| AVILA BEACH, CA | AVL | Avila | 0.954 | 0.392 | 0.023 | 1.031 | 1.031 | 253 | 38 | 102 |
| SAN RAFAEL, CA | OSM | Other Sonoma and Marine County Outer Coast ports | 0.725 | 0.465 | 0.558 | 0.861 | 1.026 | 115 | 97 | 103 |
| CORONADO, CA | OSD | Other San Diego County ports | 0.000 | 0.868 | 0.523 | 0.868 | 1.014 | 124 | 78 | 104 |
| SANTEE, CA | OSD | Other San Diego County ports | 0.000 | 0.868 | 0.519 | 0.868 | 1.012 | 125 | 78 | 105 |
| ALTOONA, WA | OCR | Other Columbia River ports | 0.000 | 0.961 | 0.310 | 0.961 | 1.010 | 176 | 50 | 106 |
| KALAMA, WA | OCR | Other Columbia River ports | 0.000 | 0.961 | 0.295 | 0.961 | 1.005 | 182 | 50 | 107 |
| WHITTIER, CA | OLA | Other LA and Orange County ports | 0.000 | 0.698 | 0.721 | 0.698 | 1.003 | 72 | 109 | 108 |
| LOS OSOS, CA | OSL | Other San Luis Obispo County ports | 0.636 | 0.585 | 0.492 | 0.864 | 0.994 | 132 | 95 | 109 |
| SEQUIM, WA | SEQ | Sequim | 0.837 | 0.380 | 0.376 | 0.919 | 0.993 | 162 | 64 | 110 |
| BLAINE, WA | BLN | Blaine | 0.861 | 0.302 | 0.384 | 0.912 | 0.990 | 160 | 66 | 111 |
| SAN LEANDRO, CA | OSF | Other San Francisco Bay and San Mateo County ports | 0.636 | 0.058 | 0.756 | 0.638 | 0.989 | 64 | 153 | 112 |
| SANTA MARIA, CA | OBV | Other Santa Barbara and Ventura County ports | 0.000 | 0.395 | 0.903 | 0.395 | 0.986 | 25 | 189 | 113 |
| SAN LUIS OBISPO, CA | OSL | Other San Luis Obispo County ports | 0.636 | 0.585 | 0.461 | 0.864 | 0.980 | 140 | 95 | 114 |
| DEPOE BAY, OR | DPO | Depoe Bay | 0.872 | 0.322 | 0.306 | 0.930 | 0.979 | 180 | 62 | 115 |
| ORANGE, CA | OLA | Other LA and Orange County ports | 0.000 | 0.698 | 0.678 | 0.698 | 0.973 | 84 | 109 | 116 |
| HALF MOON BAY, CA | PRN | Princeton/Half Moon Bay | 0.907 | 0.295 | 0.178 | 0.954 | 0.970 | 213 | 54 | 117 |
| SANTA MONICA, CA | OLA | Other LA and Orange County ports | 0.636 | 0.698 | 0.182 | 0.944 | 0.961 | 212 | 57 | 118 |
| ARCATA, CA | OHB | Other Humboldt County ports | 0.725 | 0.004 | 0.620 | 0.725 | 0.954 | 99 | 108 | 119 |
| SAN FRANCISCO, CA | SF | San Francisco | 0.686 | 0.372 | 0.543 | 0.781 | 0.951 | 118 | 102 | 120 |
| FOUNTAIN VALLEY, CA | OLA | Other LA and Orange County ports | 0.000 | 0.698 | 0.636 | 0.698 | 0.944 | 95 | 109 | 121 |
| MONTEREY, CA | MNT | Monterey | 0.830 | 0.349 | 0.279 | 0.900 | 0.942 | 187 | 73 | 122 |
| PITTSBURG, CA | OSF | Other San Francisco Bay and San Mateo County ports | 0.000 | 0.058 | 0.934 | 0.058 | 0.936 | 18 | 204 | 123 |
| ANACORTES, WA | ANA | Anacortes | 0.853 | 0.291 | 0.174 | 0.901 | 0.918 | 214 | 72 | 124 |
| BOLINAS, CA | BOL | Bolinas | 0.868 | 0.287 | 0.008 | 0.914 | 0.914 | 257 | 65 | 125 |
| SEATTLE, WA | SEA | Seattle | 0.686 | 0.554 | 0.236 | 0.882 | 0.913 | 198 | 77 | 126 |
| SUISUN CITY, CA | OSF | Other San Francisco Bay and San Mateo County ports | 0.000 | 0.058 | 0.911 | 0.058 | 0.913 | 24 | 204 | 127 |
| GOLETA, CA | OBV | Other Santa Barbara and Ventura County ports | 0.725 | 0.395 | 0.388 | 0.826 | 0.912 | 159 | 99 | 128 |
| NEHALEM, OR | NHL | Nehalem | 0.899 | 0.058 | 0.132 | 0.901 | 0.911 | 225 | 71 | 129 |
| COSTA MESA, CA | OLA | Other LA and Orange County ports | 0.000 | 0.698 | 0.581 | 0.698 | 0.908 | 108 | 109 | 130 |
| POWAY, CA | OSD | Other San Diego County ports | 0.000 | 0.868 | 0.256 | 0.868 | 0.905 | 192 | 78 | 131 |
| OCEANO, CA | OSL | Other San Luis Obispo County ports | 0.000 | 0.585 | 0.690 | 0.585 | 0.905 | 79 | 157 | 132 |
| PASADENA, CA | OLA | Other LA and Orange County ports | 0.000 | 0.698 | 0.574 | 0.698 | 0.903 | 111 | 109 | 133 |
| PETALUMA, CA | OSM | Other Sonoma and Marine County Outer Coast ports | 0.636 | 0.465 | 0.438 | 0.788 | 0.901 | 146 | 101 | 134 |
| CARLSBAD, CA | OSD | Other San Diego County ports | 0.000 | 0.868 | 0.240 | 0.868 | 0.901 | 197 | 78 | 135 |
| SANTA ROSA, CA | OSM | Other Sonoma and Marine County Outer Coast ports | 0.000 | 0.465 | 0.771 | 0.465 | 0.901 | 60 | 167 | 136 |
| KENTFIELD, CA | OSM | Other Sonoma and Marine County Outer Coast ports | 0.756 | 0.465 | 0.136 | 0.888 | 0.898 | 224 | 75 | 137 |
| CLOVERDALE, CA | OSM | Other Sonoma and Marine County Outer Coast ports | 0.000 | 0.465 | 0.764 | 0.465 | 0.894 | 62 | 167 | 138 |
| TORRANCE, CA | OLA | Other LA and Orange County ports | 0.000 | 0.698 | 0.539 | 0.698 | 0.882 | 120 | 109 | 139 |
| GUADALUPE, CA | OBV | Other Santa Barbara and Ventura County ports | 0.000 | 0.395 | 0.783 | 0.395 | 0.877 | 57 | 189 | 140 |
| DEL MAR, CA | OSD | Other San Diego County ports | 0.000 | 0.868 | 0.097 | 0.868 | 0.874 | 234 | 78 | 141 |
| ENCINITAS, CA | OSD | Other San Diego County ports | 0.000 | 0.868 | 0.066 | 0.868 | 0.871 | 242 | 78 | 142 |
| SOLANA BEACH, CA | OSD | Other San Diego County ports | 0.000 | 0.868 | 0.012 | 0.868 | 0.868 | 256 | 78 | 143 |
| ROHNERT PARK, CA | OSM | Other Sonoma and Marine County Outer Coast ports | 0.000 | 0.465 | 0.705 | 0.465 | 0.845 | 77 | 167 | 144 |
| ANTIOCH, CA | OSF | Other San Francisco Bay and San Mateo County ports | 0.000 | 0.058 | 0.841 | 0.058 | 0.843 | 42 | 204 | 145 |
| IRVINE, CA | OLA | Other LA and Orange County ports | 0.000 | 0.698 | 0.469 | 0.698 | 0.841 | 138 | 109 | 146 |
| SOUTH SAN FRANCISCO, CA | OSF | Other San Francisco Bay and San Mateo County ports | 0.686 | 0.058 | 0.477 | 0.689 | 0.838 | 136 | 148 | 147 |
| EMERYVILLE, CA | OSF | Other San Francisco Bay and San Mateo County ports | 0.686 | 0.058 | 0.465 | 0.689 | 0.831 | 139 | 148 | 148 |
| SMITH RIVER, CA | ODN | Other Del Norte County ports | 0.000 | 0.058 | 0.826 | 0.058 | 0.828 | 46 | 204 | 149 |
| SAUSALITO, CA | SLT | Sausalito | 0.795 | 0.058 | 0.213 | 0.797 | 0.825 | 203 | 100 | 150 |
| FAIRFIELD, CA | OSF | Other San Francisco Bay and San Mateo County ports | 0.000 | 0.058 | 0.822 | 0.058 | 0.824 | 47 | 204 | 151 |
| DIXON, CA | OCA | Other or unknown California ports | 0.000 | 0.353 | 0.744 | 0.353 | 0.824 | 67 | 198 | 152 |
| PINOLE, CA | OSF | Other San Francisco Bay and San Mateo County ports | 0.636 | 0.058 | 0.508 | 0.638 | 0.816 | 128 | 153 | 153 |
| RODEO, CA | OSF | Other San Francisco Bay and San Mateo County ports | 0.725 | 0.058 | 0.364 | 0.727 | 0.813 | 165 | 106 | 154 |
| GROVER BEACH, CA | OSL | Other San Luis Obispo County ports | 0.000 | 0.585 | 0.562 | 0.585 | 0.811 | 114 | 157 | 155 |
| FORTUNA, CA | OHB | Other Humboldt County ports | 0.000 | 0.004 | 0.810 | 0.004 | 0.810 | 50 | 250 | 156 |
| OAKLAND, CA | OAK | Oakland | 0.000 | 0.058 | 0.795 | 0.058 | 0.797 | 53 | 204 | 157 |
| NIPOMO, CA | OSL | Other San Luis Obispo County ports | 0.000 | 0.585 | 0.535 | 0.585 | 0.793 | 121 | 157 | 158 |
| ATASCADERO, CA | OSL | Other San Luis Obispo County ports | 0.000 | 0.585 | 0.527 | 0.585 | 0.788 | 123 | 157 | 159 |
| HUNTINGTON BEACH, CA | OLA | Other LA and Orange County ports | 0.000 | 0.698 | 0.357 | 0.698 | 0.784 | 167 | 109 | 160 |
| HAYWARD, CA | OSF | Other San Francisco Bay and San Mateo County ports | 0.000 | 0.058 | 0.779 | 0.058 | 0.781 | 58 | 204 | 161 |
| EL PASO DE ROBLES (PASO ROBLES), CA | OSL | Other San Luis Obispo County ports | 0.000 | 0.585 | 0.496 | 0.585 | 0.767 | 131 | 157 | 162 |
| SUMMERLAND, CA | OBV | Other Santa Barbara and Ventura County ports | 0.636 | 0.395 | 0.140 | 0.749 | 0.762 | 223 | 104 | 163 |
| BYRON, CA | OCA | Other or unknown California ports | 0.000 | 0.353 | 0.667 | 0.353 | 0.754 | 87 | 198 | 164 |
| SEBASTOPOL, CA | OSM | Other Sonoma and Marine County Outer Coast ports | 0.000 | 0.465 | 0.593 | 0.465 | 0.754 | 106 | 167 | 165 |
| NAPA, CA | OSF | Other San Francisco Bay and San Mateo County ports | 0.000 | 0.058 | 0.748 | 0.058 | 0.750 | 66 | 204 | 166 |
| FERNDALE, CA | OHB | Other Humboldt County ports | 0.686 | 0.004 | 0.291 | 0.686 | 0.745 | 184 | 151 | 167 |
| WINDSOR, CA | OSM | Other Sonoma and Marine County Outer Coast ports | 0.000 | 0.465 | 0.581 | 0.465 | 0.745 | 108 | 167 | 168 |
| GASQUET, CA | ODN | Other Del Norte County ports | 0.000 | 0.058 | 0.736 | 0.058 | 0.739 | 69 | 204 | 169 |
| SAN JUAN CAPISTRANO, CA | OLA | Other LA and Orange County ports | 0.000 | 0.698 | 0.225 | 0.698 | 0.733 | 201 | 109 | 170 |
| SCOTIA, CA | OHB | Other Humboldt County ports | 0.000 | 0.004 | 0.729 | 0.004 | 0.729 | 71 | 250 | 171 |
| ARROYO GRANDE, CA | OSL | Other San Luis Obispo County ports | 0.000 | 0.585 | 0.430 | 0.585 | 0.726 | 147 | 157 | 172 |
| SAN CLEMENTE, CA | OLA | Other LA and Orange County ports | 0.000 | 0.698 | 0.198 | 0.698 | 0.725 | 208 | 109 | 173 |
| CAMBRIA, CA | OSL | Other San Luis Obispo County ports | 0.000 | 0.585 | 0.426 | 0.585 | 0.724 | 149 | 157 | 174 |
| RANCHO PALOS VERDES, CA | OLA | Other LA and Orange County ports | 0.000 | 0.698 | 0.186 | 0.698 | 0.722 | 211 | 109 | 175 |
| MISSION VIEJO, CA | OLA | Other LA and Orange County ports | 0.000 | 0.698 | 0.171 | 0.698 | 0.718 | 215 | 109 | 176 |
| LOS ALAMITOS, CA | OLA | Other LA and Orange County ports | 0.000 | 0.698 | 0.147 | 0.698 | 0.713 | 221 | 109 | 177 |
| MALIBU, CA | OLA | Other LA and Orange County ports | 0.000 | 0.698 | 0.124 | 0.698 | 0.709 | 227 | 109 | 178 |
| LA CANADA FLINTRIDGE, CA | OLA | Other LA and Orange County ports | 0.000 | 0.698 | 0.120 | 0.698 | 0.708 | 228 | 109 | 179 |
| PACIFICA, CA | OSF | Other San Francisco Bay and San Mateo County ports | 0.636 | 0.058 | 0.302 | 0.638 | 0.706 | 181 | 153 | 180 |
| ANCHOR BAY, CA | OMD | Other Mendocino County ports | 0.000 | 0.442 | 0.550 | 0.442 | 0.706 | 116 | 185 | 181 |
| SEAL BEACH, CA | OLA | Other LA and Orange County ports | 0.000 | 0.698 | 0.078 | 0.698 | 0.702 | 238 | 109 | 182 |
| TOPANGA, CA | OLA | Other LA and Orange County ports | 0.000 | 0.698 | 0.035 | 0.698 | 0.699 | 250 | 109 | 183 |
| EL SEGUNDO, CA | OLA | Other LA and Orange County ports | 0.000 | 0.698 | 0.027 | 0.698 | 0.698 | 252 | 109 | 184 |
| MANHATTAN BEACH, CA | OLA | Other LA and Orange County ports | 0.000 | 0.698 | 0.000 | 0.698 | 0.698 | 259 | 109 | 185 |
| GARBERVILLE, CA | OHB | Other Humboldt County ports | 0.000 | 0.004 | 0.690 | 0.004 | 0.690 | 79 | 250 | 186 |
| WEOTT, CA | OHB | Other Humboldt County ports | 0.000 | 0.004 | 0.690 | 0.004 | 0.690 | 79 | 250 | 186 |
| SAN JOSE, CA | OSF | Other San Francisco Bay and San Mateo County ports | 0.000 | 0.058 | 0.686 | 0.058 | 0.689 | 82 | 204 | 188 |
| MARTINEZ, CA | OSF | Other San Francisco Bay and San Mateo County ports | 0.636 | 0.058 | 0.256 | 0.638 | 0.688 | 192 | 153 | 189 |
| SAN MIGUEL (SAN LUIS OBISPO COUNTY), CA | OSL | Other San Luis Obispo County ports | 0.000 | 0.585 | 0.333 | 0.585 | 0.674 | 173 | 157 | 190 |
| FORT DICK, CA | ODN | Other Del Norte County ports | 0.000 | 0.058 | 0.671 | 0.058 | 0.673 | 86 | 204 | 191 |
| CASPAR, CA | OMD | Other Mendocino County ports | 0.000 | 0.442 | 0.485 | 0.442 | 0.656 | 133 | 185 | 192 |
| MENDOCINO, CA | OMD | Other Mendocino County ports | 0.000 | 0.442 | 0.485 | 0.442 | 0.656 | 133 | 185 | 192 |
| SKAMOKAWA VALLEY, WA | LWC | Ilwaco/Chinook | 0.000 | 0.570 | 0.310 | 0.570 | 0.649 | 176 | 166 | 194 |
| LOLETA, CA | OHB | Other Humboldt County ports | 0.000 | 0.004 | 0.647 | 0.004 | 0.647 | 92 | 250 | 195 |
| DALY CITY, CA | OSF | Other San Francisco Bay and San Mateo County ports | 0.000 | 0.058 | 0.643 | 0.058 | 0.646 | 93 | 204 | 196 |
| EL SOBRANTE, CA | OSF | Other San Francisco Bay and San Mateo County ports | 0.000 | 0.058 | 0.640 | 0.058 | 0.642 | 94 | 204 | 197 |
| MCKINLEYVILLE, CA | OHB | Other Humboldt County ports | 0.000 | 0.004 | 0.628 | 0.004 | 0.628 | 97 | 250 | 198 |
| MIRANDA, CA | OHB | Other Humboldt County ports | 0.000 | 0.004 | 0.624 | 0.004 | 0.624 | 98 | 250 | 199 |
| ST. HELENA, CA | OCA | Other or unknown California ports | 0.000 | 0.353 | 0.512 | 0.353 | 0.621 | 127 | 198 | 200 |
| HEALDSBURG, CA | OSM | Other Sonoma and Marine County Outer Coast ports | 0.000 | 0.465 | 0.411 | 0.465 | 0.621 | 152 | 167 | 201 |
| SOLVANG, CA | OBV | Other Santa Barbara and Ventura County ports | 0.000 | 0.395 | 0.473 | 0.395 | 0.616 | 137 | 189 | 202 |
| BLUE LAKE, CA | OHB | Other Humboldt County ports | 0.000 | 0.004 | 0.616 | 0.004 | 0.616 | 100 | 250 | 203 |
| PISMO BEACH, CA | OSL | Other San Luis Obispo County ports | 0.000 | 0.585 | 0.190 | 0.585 | 0.615 | 210 | 157 | 204 |
| OAKLEY, CA | OSF | Other San Francisco Bay and San Mateo County ports | 0.000 | 0.058 | 0.612 | 0.058 | 0.615 | 101 | 204 | 205 |
| VACAVILLE, CA | OSF | Other San Francisco Bay and San Mateo County ports | 0.000 | 0.058 | 0.609 | 0.058 | 0.611 | 102 | 204 | 206 |
| RIO VISTA, CA | OSF | Other San Francisco Bay and San Mateo County ports | 0.000 | 0.058 | 0.605 | 0.058 | 0.607 | 103 | 204 | 207 |
| CONCORD, CA | OSF | Other San Francisco Bay and San Mateo County ports | 0.000 | 0.058 | 0.597 | 0.058 | 0.600 | 104 | 204 | 208 |
| TIBURON, CA | OSM | Other Sonoma and Marine County Outer Coast ports | 0.000 | 0.465 | 0.372 | 0.465 | 0.596 | 163 | 167 | 209 |
| GUERNEVILLE, CA | OSM | Other Sonoma and Marine County Outer Coast ports | 0.000 | 0.465 | 0.330 | 0.465 | 0.570 | 174 | 167 | 210 |
| CAMARILLO, CA | OBV | Other Santa Barbara and Ventura County ports | 0.000 | 0.395 | 0.399 | 0.395 | 0.562 | 155 | 189 | 211 |
| REDWOOD CITY, CA | OSF | Other San Francisco Bay and San Mateo County ports | 0.000 | 0.058 | 0.543 | 0.058 | 0.546 | 118 | 204 | 212 |
| YOUNTVILLE, CA | OSF | Other San Francisco Bay and San Mateo County ports | 0.000 | 0.058 | 0.531 | 0.058 | 0.534 | 122 | 204 | 213 |
| FIELDBROOK, CA | OCA | Other or unknown California ports | 0.000 | 0.353 | 0.399 | 0.353 | 0.533 | 155 | 198 | 214 |
| OCCIDENTAL, CA | OSM | Other Sonoma and Marine County Outer Coast ports | 0.000 | 0.465 | 0.248 | 0.465 | 0.527 | 195 | 167 | 215 |
| SONOMA, CA | OSM | Other Sonoma and Marine County Outer Coast ports | 0.000 | 0.465 | 0.205 | 0.465 | 0.509 | 206 | 167 | 216 |
| NOVATO, CA | OSM | Other Sonoma and Marine County Outer Coast ports | 0.000 | 0.465 | 0.194 | 0.465 | 0.504 | 209 | 167 | 217 |
| OJAI, CA | OBV | Other Santa Barbara and Ventura County ports | 0.000 | 0.395 | 0.287 | 0.395 | 0.488 | 185 | 189 | 218 |
| TIMBER COVE, CA | BDG | Bodega Bay | 0.000 | 0.333 | 0.353 | 0.333 | 0.485 | 168 | 203 | 219 |
| JENNER, CA | OSM | Other Sonoma and Marine County Outer Coast ports | 0.000 | 0.465 | 0.128 | 0.465 | 0.482 | 226 | 167 | 220 |
| NICASIO, CA | OSM | Other Sonoma and Marine County Outer Coast ports | 0.000 | 0.465 | 0.109 | 0.465 | 0.478 | 229 | 167 | 221 |
| MUIR BEACH, CA | OSM | Other Sonoma and Marine County Outer Coast ports | 0.000 | 0.465 | 0.078 | 0.465 | 0.472 | 238 | 167 | 222 |
| CORTE MADERA, CA | OSM | Other Sonoma and Marine County Outer Coast ports | 0.000 | 0.465 | 0.058 | 0.465 | 0.469 | 244 | 167 | 223 |
| INVERNESS, CA | OSM | Other Sonoma and Marine County Outer Coast ports | 0.000 | 0.465 | 0.054 | 0.465 | 0.468 | 245 | 167 | 224 |
| MILL VALLEY, CA | OSM | Other Sonoma and Marine County Outer Coast ports | 0.000 | 0.465 | 0.043 | 0.465 | 0.467 | 248 | 167 | 225 |
| STINSON BEACH, CA | OSM | Other Sonoma and Marine County Outer Coast ports | 0.000 | 0.465 | 0.039 | 0.465 | 0.467 | 249 | 167 | 226 |
| SIMI VALLEY, CA | OBV | Other Santa Barbara and Ventura County ports | 0.000 | 0.395 | 0.229 | 0.395 | 0.457 | 199 | 189 | 227 |
| ALBANY, CA | OSF | Other San Francisco Bay and San Mateo County ports | 0.000 | 0.058 | 0.450 | 0.058 | 0.453 | 143 | 204 | 228 |
| BETHEL ISLAND, CA | OCA | Other or unknown California ports | 0.000 | 0.353 | 0.271 | 0.353 | 0.445 | 189 | 198 | 229 |
| BRENTWOOD, CA | OSF | Other San Francisco Bay and San Mateo County ports | 0.000 | 0.058 | 0.430 | 0.058 | 0.434 | 147 | 204 | 230 |
| THOUSAND OAKS, CA | OBV | Other Santa Barbara and Ventura County ports | 0.000 | 0.395 | 0.159 | 0.395 | 0.426 | 218 | 189 | 231 |
| WESTLAKE VILLAGE, CA | OBV | Other Santa Barbara and Ventura County ports | 0.000 | 0.395 | 0.031 | 0.395 | 0.397 | 251 | 189 | 232 |
| ORICK, CA | OHB | Other Humboldt County ports | 0.000 | 0.004 | 0.392 | 0.004 | 0.392 | 157 | 250 | 233 |
| EL CERRITO, CA | OSF | Other San Francisco Bay and San Mateo County ports | 0.000 | 0.058 | 0.380 | 0.058 | 0.384 | 161 | 204 | 234 |
| BURLINGAME, CA | OSF | Other San Francisco Bay and San Mateo County ports | 0.000 | 0.058 | 0.368 | 0.058 | 0.373 | 164 | 204 | 235 |
| PALO ALTO, CA | OSF | Other San Francisco Bay and San Mateo County ports | 0.000 | 0.058 | 0.349 | 0.058 | 0.354 | 169 | 204 | 236 |
| FOSTER CITY, CA | OSF | Other San Francisco Bay and San Mateo County ports | 0.000 | 0.058 | 0.345 | 0.058 | 0.350 | 170 | 204 | 237 |
| SUNNYVALE, CA | OSF | Other San Francisco Bay and San Mateo County ports | 0.000 | 0.058 | 0.341 | 0.058 | 0.346 | 171 | 204 | 238 |
| SAN BRUNO, CA | OSF | Other San Francisco Bay and San Mateo County ports | 0.000 | 0.058 | 0.337 | 0.058 | 0.342 | 172 | 204 | 239 |
| FREMONT, CA | OSF | Other San Francisco Bay and San Mateo County ports | 0.000 | 0.058 | 0.295 | 0.058 | 0.300 | 182 | 204 | 240 |
| CAMPBELL, CA | OSF | Other San Francisco Bay and San Mateo County ports | 0.000 | 0.058 | 0.283 | 0.058 | 0.289 | 186 | 204 | 241 |
| PLEASANT HILL, CA | OSF | Other San Francisco Bay and San Mateo County ports | 0.000 | 0.058 | 0.275 | 0.058 | 0.281 | 188 | 204 | 242 |
| SAN MATEO, CA | OSF | Other San Francisco Bay and San Mateo County ports | 0.000 | 0.058 | 0.267 | 0.058 | 0.274 | 190 | 204 | 243 |
| WESTHAVEN-MOONSTONE, CA | OHB | Other Humboldt County ports | 0.000 | 0.004 | 0.264 | 0.004 | 0.264 | 191 | 250 | 244 |
| NEWARK, CA | OSF | Other San Francisco Bay and San Mateo County ports | 0.000 | 0.058 | 0.252 | 0.058 | 0.259 | 194 | 204 | 245 |
| SAN CARLOS, CA | OSF | Other San Francisco Bay and San Mateo County ports | 0.000 | 0.058 | 0.229 | 0.058 | 0.236 | 199 | 204 | 246 |
| MOUNTAIN VIEW CITY, CA | OSF | Other San Francisco Bay and San Mateo County ports | 0.000 | 0.058 | 0.213 | 0.058 | 0.221 | 203 | 204 | 247 |
| PESCADERO, CA | OSF | Other San Francisco Bay and San Mateo County ports | 0.000 | 0.058 | 0.209 | 0.058 | 0.217 | 205 | 204 | 248 |
| BENICIA, CA | OSF | Other San Francisco Bay and San Mateo County ports | 0.000 | 0.058 | 0.163 | 0.058 | 0.173 | 217 | 204 | 249 |
| LOS ALTOS, CA | OSF | Other San Francisco Bay and San Mateo County ports | 0.000 | 0.058 | 0.151 | 0.058 | 0.162 | 220 | 204 | 250 |
| MONTARA, CA | OSF | Other San Francisco Bay and San Mateo County ports | 0.000 | 0.058 | 0.143 | 0.058 | 0.155 | 222 | 204 | 251 |
| WALNUT CREEK, CA | OSF | Other San Francisco Bay and San Mateo County ports | 0.000 | 0.058 | 0.105 | 0.058 | 0.120 | 232 | 204 | 252 |
| PLEASANTON, CA | OSF | Other San Francisco Bay and San Mateo County ports | 0.000 | 0.058 | 0.093 | 0.058 | 0.110 | 235 | 204 | 253 |
| LIVERMORE, CA | OSF | Other San Francisco Bay and San Mateo County ports | 0.000 | 0.058 | 0.089 | 0.058 | 0.106 | 236 | 204 | 254 |
| MOSS BEACH, CA | OSF | Other San Francisco Bay and San Mateo County ports | 0.000 | 0.058 | 0.074 | 0.058 | 0.094 | 240 | 204 | 255 |
| LOS GATOS, CA | OSF | Other San Francisco Bay and San Mateo County ports | 0.000 | 0.058 | 0.070 | 0.058 | 0.091 | 241 | 204 | 256 |
| LAFAYETTE, CA | OSF | Other San Francisco Bay and San Mateo County ports | 0.000 | 0.058 | 0.047 | 0.058 | 0.075 | 247 | 204 | 257 |
| DANVILLE, CA | OSF | Other San Francisco Bay and San Mateo County ports | 0.000 | 0.058 | 0.019 | 0.058 | 0.061 | 254 | 204 | 258 |
| ALAMO, CA | OSF | Other San Francisco Bay and San Mateo County ports | 0.000 | 0.058 | 0.016 | 0.058 | 0.060 | 255 | 204 | 259 |
