# Supplementary material for: Social-ecological vulnerability of fishing communities to climate change: A U.S. West Coast case study
Source: PLoS One. 2022 Aug 17;17(8):e0272120. doi: 10.1371/journal.pone.0272120 (PMC9385011; doi:10.1371/journal.pone.0272120)
Supplement: S6 Table — For each community, social indicators, percentile ranked, that make up adaptive capacity within four themes: 1) socioeconomic—persons below poverty, unemployed civilians, per capita income, persons with no high school diploma; 2) household composition/disability—persons age 65+, persons age 17 and less, noninstitutionalized population with a disability, number of single parent households; 3) minority status/language—persons of minority, persons older than 5 that don’t speak English well; and 4) housing/transportation—housing structures with 10+ units, estimates of mobile homes, households with more people than rooms, households with no vehicles, and persons in institutionalized group quarters. Communities are listed from lowest adaptive capacity to highest. (DOCX) [file pone.0272120.s011.docx]

| Community | Multi-unit home | Group quarters | Crowding | No vehicle | Mobile home | Minority | Limited English | Disability | Age 17- | Age 65+ | Single parent | Unemploy | No high. diploma | Poverty | Income | Adapt. cap. |
| --- | --- | --- | --- | --- | --- | --- | --- | --- | --- | --- | --- | --- | --- | --- | --- | --- |
| NATIONAL CITY, CA | 0.79 | 0.92 | 0.95 | 0.87 | 0.57 | 0.95 | 0.94 | 0.54 | 0.59 | 0.12 | 0.90 | 0.93 | 0.95 | 0.92 | 0.96 | 1.00 |
| CHULA VISTA, CA | 0.83 | 0.81 | 0.91 | 0.64 | 0.67 | 0.92 | 0.89 | 0.43 | 0.81 | 0.21 | 0.86 | 0.92 | 0.92 | 0.64 | 0.83 | 1.00 |
| SOUTH GATE, CA | 0.62 | 0.73 | 0.98 | 0.87 | 0.34 | 0.99 | 0.99 | 0.18 | 0.98 | 0.02 | 0.99 | 0.89 | 0.98 | 0.94 | 0.99 | 0.99 |
| SANTA PAULA, CA | 0.33 | 0.56 | 0.89 | 0.75 | 0.84 | 0.93 | 0.97 | 0.55 | 0.98 | 0.19 | 0.84 | 0.83 | 0.97 | 0.78 | 0.94 | 0.99 |
| OXNARD, CA | 0.69 | 0.66 | 0.97 | 0.37 | 0.65 | 0.91 | 0.97 | 0.39 | 0.97 | 0.03 | 0.95 | 0.69 | 0.96 | 0.79 | 0.91 | 0.98 |
| BELL GARDENS, CA | 0.54 | 0.58 | 0.99 | 0.84 | 0.58 | 0.98 | 0.99 | 0.12 | 0.99 | 0.01 | 0.98 | 0.81 | 0.99 | 0.97 | 1.00 | 0.98 |
| LYNWOOD, CA | 0.60 | 0.63 | 0.99 | 0.90 | 0.29 | 1.00 | 0.98 | 0.12 | 0.99 | 0.00 | 1.00 | 0.95 | 1.00 | 0.96 | 1.00 | 0.98 |
| CRESCENT CITY, CA | 0.31 | 0.97 | 0.49 | 0.95 | 0.97 | 0.56 | 0.33 | 0.93 | 0.83 | 0.41 | 0.86 | 0.95 | 0.91 | 0.99 | 0.96 | 0.97 |
| PARAMOUNT, CA | 0.86 | 0.10 | 0.98 | 0.67 | 0.73 | 0.98 | 0.95 | 0.21 | 0.97 | 0.00 | 0.98 | 0.82 | 0.98 | 0.87 | 0.98 | 0.97 |
| UKIAH, CA | 0.55 | 0.88 | 0.66 | 0.72 | 0.66 | 0.61 | 0.83 | 0.83 | 0.82 | 0.49 | 0.68 | 0.91 | 0.79 | 0.92 | 0.78 | 0.97 |
| VERNON, CA | 0.88 | 0.42 | 1.00 | 0.97 | 0.10 | 1.00 | 1.00 | 0.20 | 0.97 | 0.01 | 1.00 | 0.86 | 1.00 | 0.98 | 0.97 | 0.96 |
| SANTA ANA, CA | 0.92 | 0.62 | 1.00 | 0.70 | 0.59 | 0.95 | 0.98 | 0.09 | 0.93 | 0.02 | 0.91 | 0.49 | 0.98 | 0.85 | 0.95 | 0.96 |
| PORT HUENEME, CA | 0.66 | 0.84 | 0.97 | 0.18 | 0.35 | 0.88 | 0.95 | 0.48 | 0.90 | 0.04 | 0.95 | 0.66 | 0.95 | 0.71 | 0.88 | 0.95 |
| KLAMATH, CA | 0.07 | 0.96 | 0.69 | 0.91 | 1.00 | 0.66 | 0.04 | 1.00 | 0.61 | 0.57 | 0.78 | 0.98 | 0.87 | 1.00 | 0.98 | 0.95 |
| LOMPOC, CA | 0.41 | 0.95 | 0.88 | 0.71 | 0.57 | 0.78 | 0.81 | 0.64 | 0.90 | 0.09 | 0.91 | 0.88 | 0.86 | 0.67 | 0.84 | 0.95 |
| EL CAJON, CA | 0.93 | 0.63 | 0.82 | 0.76 | 0.68 | 0.56 | 0.82 | 0.59 | 0.86 | 0.22 | 0.83 | 0.89 | 0.76 | 0.83 | 0.75 | 0.94 |
| IMPERIAL BEACH, CA | 0.76 | 0.34 | 0.90 | 0.53 | 0.50 | 0.85 | 0.84 | 0.60 | 0.79 | 0.26 | 0.92 | 0.92 | 0.87 | 0.83 | 0.79 | 0.94 |
| PITTSBURG, CA | 0.62 | 0.25 | 0.88 | 0.83 | 0.44 | 0.94 | 0.87 | 0.70 | 0.93 | 0.03 | 0.97 | 0.95 | 0.91 | 0.79 | 0.88 | 0.93 |
| INGLEWOOD, CA | 0.84 | 0.41 | 0.92 | 0.93 | 0.09 | 0.98 | 0.90 | 0.57 | 0.73 | 0.11 | 0.97 | 0.91 | 0.95 | 0.85 | 0.89 | 0.93 |
| KELSO, WA | 0.59 | 0.78 | 0.49 | 0.99 | 0.93 | 0.24 | 0.30 | 0.98 | 0.71 | 0.51 | 0.68 | 0.97 | 0.86 | 0.98 | 0.93 | 0.93 |
| GARDENA, CA | 0.96 | 0.38 | 0.93 | 0.79 | 0.38 | 0.97 | 0.91 | 0.43 | 0.64 | 0.31 | 0.79 | 0.74 | 0.88 | 0.78 | 0.83 | 0.92 |
| CARSON, CA | 0.50 | 0.32 | 0.93 | 0.48 | 0.79 | 0.96 | 0.88 | 0.47 | 0.62 | 0.33 | 0.82 | 0.81 | 0.94 | 0.66 | 0.88 | 0.92 |
| NEAH BAY, WA | 0.00 | 0.56 | 0.84 | 0.95 | 0.98 | 0.97 | 0.00 | 0.72 | 1.00 | 0.03 | 0.99 | 1.00 | 0.84 | 0.95 | 0.98 | 0.91 |
| SUISUN CITY, CA | 0.53 | 0.75 | 0.83 | 0.63 | 0.41 | 0.84 | 0.78 | 0.63 | 0.88 | 0.02 | 0.96 | 0.84 | 0.84 | 0.66 | 0.90 | 0.91 |
| LANCASTER, CA | 0.48 | 0.84 | 0.50 | 0.81 | 0.74 | 0.79 | 0.59 | 0.35 | 0.95 | 0.05 | 0.94 | 0.83 | 0.86 | 0.95 | 0.93 | 0.90 |
| SANTA MARIA, CA | 0.51 | 0.34 | 0.97 | 0.46 | 0.60 | 0.85 | 0.97 | 0.36 | 0.98 | 0.19 | 0.93 | 0.56 | 0.97 | 0.74 | 0.85 | 0.90 |
| ANAHEIM, CA | 0.89 | 0.74 | 0.95 | 0.51 | 0.47 | 0.87 | 0.93 | 0.19 | 0.89 | 0.07 | 0.83 | 0.61 | 0.92 | 0.72 | 0.71 | 0.90 |
| LONGVIEW, WA | 0.57 | 0.64 | 0.30 | 0.95 | 0.85 | 0.21 | 0.26 | 0.99 | 0.79 | 0.61 | 0.90 | 0.86 | 0.82 | 0.96 | 0.92 | 0.90 |
| LOS ANGELES, CA | 0.99 | 0.67 | 0.96 | 0.97 | 0.16 | 0.86 | 0.95 | 0.38 | 0.53 | 0.12 | 0.86 | 0.78 | 0.94 | 0.90 | 0.56 | 0.89 |
| MOSS LANDING, CA | 0.00 | 0.07 | 0.86 | 0.28 | 0.94 | 0.82 | 0.96 | 0.44 | 0.96 | 0.40 | 0.91 | 0.94 | 0.97 | 0.42 | 0.94 | 0.89 |
| LEMON GROVE, CA | 0.59 | 0.35 | 0.84 | 0.72 | 0.26 | 0.88 | 0.83 | 0.52 | 0.89 | 0.08 | 0.88 | 0.90 | 0.90 | 0.74 | 0.91 | 0.88 |
| NORWALK, CA | 0.73 | 0.79 | 0.96 | 0.57 | 0.23 | 0.96 | 0.93 | 0.31 | 0.75 | 0.12 | 0.72 | 0.59 | 0.93 | 0.62 | 0.90 | 0.88 |
| ESCONDIDO, CA | 0.83 | 0.55 | 0.89 | 0.64 | 0.72 | 0.77 | 0.87 | 0.41 | 0.83 | 0.19 | 0.81 | 0.38 | 0.89 | 0.68 | 0.70 | 0.88 |
| FIELDS LANDING, CA | 0.10 | 0.83 | 0.69 | 0.09 | 0.92 | 0.43 | 0.66 | 0.75 | 0.76 | 0.50 | 0.79 | 0.84 | 0.63 | 0.68 | 0.87 | 0.87 |
| FORT BRAGG, CA | 0.16 | 0.80 | 0.62 | 0.74 | 0.75 | 0.38 | 0.59 | 0.90 | 0.45 | 0.77 | 0.93 | 0.67 | 0.71 | 0.86 | 0.84 | 0.87 |
| COVINA, CA | 0.77 | 0.46 | 0.83 | 0.50 | 0.56 | 0.92 | 0.85 | 0.47 | 0.80 | 0.22 | 0.81 | 0.71 | 0.85 | 0.38 | 0.81 | 0.86 |
| VISTA, CA | 0.74 | 0.58 | 0.93 | 0.33 | 0.71 | 0.76 | 0.84 | 0.17 | 0.84 | 0.24 | 0.79 | 0.42 | 0.88 | 0.67 | 0.78 | 0.86 |
| WESTMINSTER, CA | 0.72 | 0.28 | 0.88 | 0.61 | 0.64 | 0.86 | 0.96 | 0.54 | 0.52 | 0.37 | 0.61 | 0.55 | 0.88 | 0.74 | 0.74 | 0.86 |
| FULLERTON, CA | 0.86 | 0.64 | 0.91 | 0.56 | 0.38 | 0.84 | 0.90 | 0.28 | 0.74 | 0.20 | 0.71 | 0.70 | 0.83 | 0.62 | 0.63 | 0.85 |
| WILLITS, CA | 0.25 | 0.28 | 0.45 | 0.94 | 0.81 | 0.32 | 0.16 | 0.91 | 0.80 | 0.73 | 0.97 | 1.00 | 0.64 | 0.94 | 0.93 | 0.85 |
| SPRING VALLEY, CA | 0.67 | 0.29 | 0.52 | 0.72 | 0.67 | 0.77 | 0.54 | 0.72 | 0.85 | 0.34 | 0.85 | 0.82 | 0.71 | 0.63 | 0.71 | 0.84 |
| ANTIOCH, CA | 0.52 | 0.24 | 0.58 | 0.66 | 0.37 | 0.83 | 0.71 | 0.77 | 0.90 | 0.13 | 0.94 | 0.91 | 0.75 | 0.71 | 0.72 | 0.84 |
| VALLEJO, CA | 0.52 | 0.39 | 0.69 | 0.77 | 0.39 | 0.89 | 0.66 | 0.62 | 0.69 | 0.28 | 0.92 | 0.90 | 0.70 | 0.73 | 0.68 | 0.84 |
| ALHAMBRA, CA | 0.81 | 0.60 | 0.91 | 0.88 | 0.05 | 0.97 | 0.98 | 0.23 | 0.30 | 0.53 | 0.57 | 0.58 | 0.91 | 0.77 | 0.70 | 0.83 |
| WALNUT, CA | 0.57 | 0.97 | 0.58 | 0.17 | 0.59 | 0.95 | 0.91 | 0.36 | 0.33 | 0.46 | 0.48 | 0.79 | 0.64 | 0.57 | 0.66 | 0.83 |
| SMITH RIVER, CA | 0.06 | 0.99 | 0.59 | 0.68 | 0.99 | 0.64 | 0.19 | 0.85 | 0.24 | 0.48 | 0.27 | 0.93 | 0.93 | 0.80 | 0.87 | 0.83 |
| FAIRFIELD, CA | 0.53 | 0.78 | 0.72 | 0.43 | 0.28 | 0.81 | 0.69 | 0.59 | 0.88 | 0.10 | 0.90 | 0.76 | 0.77 | 0.41 | 0.63 | 0.82 |
| FALLBROOK, CA | 0.52 | 0.90 | 0.78 | 0.22 | 0.52 | 0.66 | 0.67 | 0.40 | 0.66 | 0.53 | 0.66 | 0.46 | 0.81 | 0.57 | 0.69 | 0.81 |
| UPLAND, CA | 0.58 | 0.86 | 0.73 | 0.38 | 0.29 | 0.81 | 0.77 | 0.42 | 0.77 | 0.23 | 0.75 | 0.62 | 0.74 | 0.63 | 0.62 | 0.81 |
| FORTUNA, CA | 0.21 | 0.33 | 0.65 | 0.66 | 0.82 | 0.29 | 0.34 | 0.84 | 0.86 | 0.55 | 0.71 | 0.74 | 0.72 | 0.83 | 0.91 | 0.81 |
| TACOMA, WA | 0.88 | 0.83 | 0.36 | 0.89 | 0.25 | 0.62 | 0.63 | 0.78 | 0.66 | 0.17 | 0.83 | 0.65 | 0.66 | 0.82 | 0.66 | 0.81 |
| RICHMOND, CA | 0.64 | 0.30 | 0.83 | 0.84 | 0.19 | 0.90 | 0.93 | 0.52 | 0.58 | 0.23 | 0.87 | 0.68 | 0.90 | 0.66 | 0.56 | 0.80 |
| OAKLAND, CA | 0.94 | 0.78 | 0.81 | 0.99 | 0.10 | 0.82 | 0.88 | 0.53 | 0.34 | 0.21 | 0.70 | 0.68 | 0.83 | 0.82 | 0.34 | 0.79 |
| TILLAMOOK, OR | 0.40 | 0.75 | 0.36 | 0.90 | 0.71 | 0.20 | 0.42 | 0.92 | 0.50 | 0.72 | 0.84 | 0.50 | 0.66 | 0.93 | 0.86 | 0.79 |
| MARIETTA-ALDERWOOD, WA | 0.80 | 0.43 | 0.41 | 0.78 | 0.76 | 0.45 | 0.29 | 0.78 | 0.70 | 0.38 | 0.88 | 0.67 | 0.48 | 0.86 | 0.81 | 0.79 |
| EUREKA, CA | 0.26 | 0.85 | 0.36 | 0.94 | 0.54 | 0.36 | 0.43 | 0.86 | 0.34 | 0.48 | 0.89 | 0.88 | 0.67 | 0.93 | 0.86 | 0.79 |
| GUADALUPE, CA | 0.27 | 0.00 | 0.98 | 0.73 | 0.07 | 0.99 | 1.00 | 0.15 | 1.00 | 0.04 | 0.98 | 0.37 | 0.99 | 0.84 | 0.99 | 0.78 |
| HAYWARD, CA | 0.87 | 0.59 | 0.94 | 0.61 | 0.46 | 0.93 | 0.88 | 0.26 | 0.64 | 0.13 | 0.72 | 0.43 | 0.84 | 0.37 | 0.58 | 0.78 |
| HAWAIIAN GARDENS, CA | 0.78 | 0.05 | 0.90 | 0.49 | 0.43 | 0.84 | 0.85 | 0.24 | 0.65 | 0.11 | 0.82 | 0.55 | 0.90 | 0.69 | 0.80 | 0.78 |
| SANTA ROSA, CA | 0.60 | 0.70 | 0.71 | 0.69 | 0.55 | 0.57 | 0.70 | 0.64 | 0.49 | 0.57 | 0.69 | 0.53 | 0.75 | 0.56 | 0.51 | 0.77 |
| GLENDALE, CA | 0.97 | 0.82 | 0.72 | 0.93 | 0.07 | 0.64 | 0.94 | 0.66 | 0.32 | 0.47 | 0.33 | 0.74 | 0.73 | 0.67 | 0.47 | 0.77 |
| CLOVERDALE, CA | 0.43 | 0.45 | 0.81 | 0.86 | 0.53 | 0.57 | 0.91 | 0.83 | 0.82 | 0.58 | 0.56 | 0.09 | 0.85 | 0.43 | 0.68 | 0.76 |
| SAN MARCOS, CA | 0.83 | 0.41 | 0.75 | 0.34 | 0.80 | 0.71 | 0.80 | 0.25 | 0.88 | 0.27 | 0.62 | 0.34 | 0.77 | 0.55 | 0.53 | 0.76 |
| SAN LEANDRO, CA | 0.70 | 0.19 | 0.87 | 0.79 | 0.22 | 0.91 | 0.92 | 0.40 | 0.60 | 0.27 | 0.76 | 0.58 | 0.89 | 0.54 | 0.62 | 0.76 |
| BANDON, OR | 0.36 | 0.69 | 0.32 | 0.88 | 0.90 | 0.05 | 0.10 | 1.00 | 0.17 | 0.96 | 0.65 | 0.87 | 0.59 | 0.88 | 0.92 | 0.75 |
| NAPA, CA | 0.64 | 0.87 | 0.71 | 0.86 | 0.35 | 0.62 | 0.83 | 0.70 | 0.57 | 0.54 | 0.57 | 0.33 | 0.81 | 0.45 | 0.41 | 0.75 |
| DIXON, CA | 0.29 | 0.07 | 0.79 | 0.07 | 0.62 | 0.69 | 0.86 | 0.40 | 0.91 | 0.18 | 0.89 | 0.69 | 0.93 | 0.40 | 0.69 | 0.74 |
| SAN DIEGO, CA | 0.88 | 0.86 | 0.74 | 0.69 | 0.24 | 0.74 | 0.78 | 0.26 | 0.45 | 0.16 | 0.67 | 0.72 | 0.72 | 0.69 | 0.47 | 0.74 |
| GASQUET, CA | 0.08 | 0.33 | 0.86 | 0.91 | 0.98 | 0.50 | 0.28 | 0.84 | 0.68 | 0.73 | 0.24 | 0.75 | 0.69 | 0.80 | 0.64 | 0.74 |
| ALAMEDA, CA | 0.98 | 0.81 | 0.78 | 0.96 | 0.12 | 0.80 | 0.86 | 0.45 | 0.31 | 0.14 | 0.59 | 0.66 | 0.80 | 0.75 | 0.30 | 0.73 |
| SCOTIA, CA | 0.12 | 0.71 | 0.21 | 0.93 | 0.74 | 0.15 | 0.12 | 0.91 | 0.91 | 0.26 | 0.96 | 0.96 | 0.76 | 0.58 | 0.95 | 0.73 |
| WHITTIER, CA | 0.68 | 0.37 | 0.85 | 0.47 | 0.22 | 0.91 | 0.81 | 0.30 | 0.81 | 0.22 | 0.69 | 0.41 | 0.83 | 0.49 | 0.71 | 0.72 |
| LA PUSH, WA | 0.11 | 0.54 | 0.59 | 0.31 | 0.96 | 0.49 | 0.03 | 0.95 | 0.24 | 0.74 | 0.47 | 0.96 | 0.54 | 0.91 | 0.77 | 0.72 |
| COOS BAY, OR | 0.37 | 0.48 | 0.37 | 0.89 | 0.87 | 0.15 | 0.09 | 0.95 | 0.47 | 0.75 | 0.62 | 0.87 | 0.63 | 0.84 | 0.82 | 0.72 |
| EVERETT, WA | 0.91 | 0.76 | 0.41 | 0.85 | 0.50 | 0.48 | 0.64 | 0.76 | 0.63 | 0.09 | 0.75 | 0.53 | 0.60 | 0.61 | 0.60 | 0.71 |
| BONITA, CA | 0.64 | 0.33 | 0.76 | 0.19 | 0.14 | 0.90 | 0.73 | 0.42 | 0.59 | 0.44 | 0.66 | 0.86 | 0.76 | 0.34 | 0.67 | 0.71 |
| ROHNERT PARK, CA | 0.76 | 0.69 | 0.70 | 0.54 | 0.68 | 0.58 | 0.67 | 0.49 | 0.55 | 0.10 | 0.71 | 0.32 | 0.64 | 0.60 | 0.65 | 0.71 |
| SAN BUENAVENTURA (VENTURA), CA | 0.69 | 0.60 | 0.47 | 0.71 | 0.61 | 0.63 | 0.65 | 0.63 | 0.57 | 0.47 | 0.69 | 0.49 | 0.61 | 0.50 | 0.50 | 0.70 |
| GARBERVILLE, CA | 0.19 | 0.72 | 0.76 | 0.58 | 0.83 | 0.05 | 0.22 | 0.76 | 0.05 | 0.59 | 0.84 | 0.78 | 0.64 | 0.89 | 0.76 | 0.69 |
| OCEANO, CA | 0.37 | 0.25 | 0.57 | 0.26 | 0.84 | 0.54 | 0.60 | 0.74 | 0.62 | 0.68 | 0.78 | 0.42 | 0.62 | 0.45 | 0.60 | 0.69 |
| WEOTT, CA | 0.19 | 0.72 | 0.76 | 0.58 | 0.83 | 0.05 | 0.22 | 0.76 | 0.05 | 0.59 | 0.84 | 0.78 | 0.64 | 0.89 | 0.76 | 0.69 |
| SAN JOSE, CA | 0.80 | 0.61 | 0.87 | 0.59 | 0.38 | 0.87 | 0.89 | 0.17 | 0.76 | 0.15 | 0.57 | 0.44 | 0.81 | 0.37 | 0.34 | 0.69 |
| PORT ANGELES, WA | 0.56 | 0.61 | 0.22 | 0.91 | 0.69 | 0.12 | 0.11 | 0.89 | 0.31 | 0.70 | 0.74 | 0.85 | 0.50 | 0.91 | 0.83 | 0.68 |
| ORANGE, CA | 0.72 | 0.73 | 0.79 | 0.22 | 0.36 | 0.75 | 0.81 | 0.14 | 0.74 | 0.20 | 0.55 | 0.43 | 0.79 | 0.53 | 0.42 | 0.68 |
| WESTPORT, WA | 0.61 | 1.00 | 0.00 | 0.76 | 0.97 | 0.24 | 0.27 | 0.92 | 0.03 | 0.10 | 0.59 | 0.93 | 0.38 | 0.91 | 0.97 | 0.67 |
| FORT DICK, CA | 0.00 | 1.00 | 0.05 | 0.05 | 0.99 | 0.74 | 0.12 | 0.88 | 0.05 | 0.06 | 0.29 | 0.99 | 0.96 | 0.81 | 0.97 | 0.67 |
| BYRON, CA | 0.17 | 0.65 | 0.60 | 0.48 | 0.46 | 0.62 | 0.52 | 0.57 | 0.93 | 0.45 | 0.63 | 0.80 | 0.58 | 0.45 | 0.49 | 0.67 |
| VANCOUVER, WA | 0.71 | 0.55 | 0.41 | 0.70 | 0.45 | 0.35 | 0.46 | 0.72 | 0.81 | 0.29 | 0.74 | 0.61 | 0.53 | 0.59 | 0.67 | 0.66 |
| LA MESA, CA | 0.95 | 0.51 | 0.51 | 0.81 | 0.14 | 0.65 | 0.49 | 0.56 | 0.48 | 0.38 | 0.72 | 0.76 | 0.56 | 0.60 | 0.57 | 0.66 |
| SHELTER COVE, CA | 0.19 | 0.85 | 0.84 | 0.66 | 0.79 | 0.04 | 0.38 | 0.65 | 0.02 | 0.76 | 0.60 | 0.76 | 0.62 | 0.72 | 0.64 | 0.66 |
| SANTA BARBARA, CA | 0.84 | 0.90 | 0.74 | 0.80 | 0.33 | 0.57 | 0.76 | 0.35 | 0.16 | 0.63 | 0.52 | 0.45 | 0.68 | 0.60 | 0.29 | 0.65 |
| LOLETA, CA | 0.15 | 0.09 | 0.59 | 0.79 | 0.66 | 0.26 | 0.15 | 0.78 | 0.87 | 0.33 | 0.95 | 0.76 | 0.36 | 0.97 | 0.89 | 0.65 |
| DALY CITY, CA | 0.86 | 0.31 | 0.92 | 0.92 | 0.24 | 0.94 | 0.92 | 0.33 | 0.21 | 0.43 | 0.49 | 0.45 | 0.80 | 0.38 | 0.52 | 0.64 |
| EL SOBRANTE, CA | 0.35 | 0.15 | 0.59 | 0.30 | 0.53 | 0.83 | 0.72 | 0.58 | 0.53 | 0.36 | 0.64 | 0.71 | 0.79 | 0.34 | 0.57 | 0.64 |
| FOUNTAIN VALLEY, CA | 0.67 | 0.28 | 0.67 | 0.38 | 0.57 | 0.76 | 0.86 | 0.50 | 0.40 | 0.57 | 0.35 | 0.36 | 0.71 | 0.43 | 0.57 | 0.64 |
| WINCHESTER BAY, OR | 0.41 | 0.41 | 0.53 | 0.82 | 0.93 | 0.04 | 0.09 | 0.93 | 0.39 | 0.84 | 0.21 | 0.21 | 0.82 | 0.89 | 0.95 | 0.63 |
| MCKINLEYVILLE, CA | 0.17 | 0.77 | 0.42 | 0.60 | 0.82 | 0.19 | 0.02 | 0.79 | 0.38 | 0.50 | 0.78 | 0.87 | 0.29 | 0.90 | 0.72 | 0.63 |
| MIRANDA, CA | 0.19 | 0.43 | 0.65 | 0.43 | 0.85 | 0.06 | 0.06 | 0.84 | 0.17 | 0.18 | 0.93 | 0.79 | 0.67 | 0.95 | 0.85 | 0.62 |
| ARCATA, CA | 0.50 | 0.91 | 0.31 | 0.83 | 0.72 | 0.28 | 0.14 | 0.69 | 0.13 | 0.25 | 0.59 | 0.96 | 0.30 | 1.00 | 0.80 | 0.62 |
| BLUE LAKE, CA | 0.43 | 0.94 | 0.21 | 0.76 | 0.88 | 0.28 | 0.01 | 0.71 | 0.23 | 0.48 | 0.60 | 0.98 | 0.19 | 0.97 | 0.66 | 0.62 |
| OAKLEY, CA | 0.26 | 0.09 | 0.48 | 0.38 | 0.54 | 0.75 | 0.61 | 0.66 | 0.96 | 0.05 | 0.88 | 0.62 | 0.69 | 0.34 | 0.62 | 0.61 |
| VACAVILLE, CA | 0.41 | 0.88 | 0.33 | 0.27 | 0.47 | 0.67 | 0.48 | 0.55 | 0.75 | 0.31 | 0.77 | 0.52 | 0.60 | 0.22 | 0.52 | 0.61 |
| RIO VISTA, CA | 0.09 | 0.24 | 0.39 | 0.28 | 0.95 | 0.43 | 0.38 | 0.94 | 0.04 | 0.93 | 0.04 | 0.90 | 0.54 | 0.87 | 0.69 | 0.60 |
| CONCORD, CA | 0.85 | 0.36 | 0.68 | 0.68 | 0.31 | 0.70 | 0.79 | 0.55 | 0.46 | 0.35 | 0.53 | 0.51 | 0.59 | 0.36 | 0.38 | 0.60 |
| NEWPORT, OR | 0.45 | 0.74 | 0.09 | 0.80 | 0.79 | 0.18 | 0.24 | 0.88 | 0.10 | 0.84 | 0.40 | 0.66 | 0.46 | 0.81 | 0.79 | 0.60 |
| SEBASTOPOL, CA | 0.34 | 0.64 | 0.46 | 0.09 | 0.47 | 0.41 | 0.66 | 0.71 | 0.33 | 0.67 | 0.70 | 0.52 | 0.59 | 0.41 | 0.40 | 0.59 |
| SAN SIMEON, CA | 0.48 | 0.62 | 0.95 | 0.34 | 0.84 | 0.34 | 0.40 | 0.44 | 0.26 | 0.81 | 0.00 | 0.72 | 0.55 | 0.38 | 0.48 | 0.59 |
| COSTA MESA, CA | 0.97 | 0.52 | 0.82 | 0.31 | 0.30 | 0.67 | 0.78 | 0.13 | 0.46 | 0.07 | 0.58 | 0.29 | 0.73 | 0.52 | 0.37 | 0.58 |
| WINDSOR, CA | 0.27 | 0.59 | 0.55 | 0.41 | 0.70 | 0.53 | 0.54 | 0.69 | 0.47 | 0.60 | 0.55 | 0.41 | 0.74 | 0.18 | 0.39 | 0.58 |
| SANTA CRUZ, CA | 0.65 | 0.93 | 0.56 | 0.78 | 0.48 | 0.44 | 0.45 | 0.41 | 0.17 | 0.38 | 0.40 | 0.78 | 0.31 | 0.86 | 0.38 | 0.58 |
| PASADENA, CA | 0.87 | 0.57 | 0.57 | 0.82 | 0.11 | 0.79 | 0.82 | 0.34 | 0.41 | 0.53 | 0.41 | 0.30 | 0.60 | 0.58 | 0.26 | 0.57 |
| FLORENCE, OR | 0.33 | 0.27 | 0.25 | 0.65 | 0.94 | 0.02 | 0.11 | 0.99 | 0.08 | 0.98 | 0.37 | 0.76 | 0.39 | 0.88 | 0.78 | 0.57 |
| ILWACO, WA | 0.38 | 0.66 | 0.07 | 0.43 | 0.93 | 0.10 | 0.33 | 0.93 | 0.52 | 0.83 | 0.29 | 0.07 | 0.49 | 0.71 | 0.79 | 0.57 |
| GROVER BEACH, CA | 0.42 | 0.13 | 0.48 | 0.40 | 0.78 | 0.49 | 0.55 | 0.65 | 0.41 | 0.69 | 0.74 | 0.28 | 0.57 | 0.46 | 0.43 | 0.56 |
| SAN RAFAEL, CA | 0.82 | 0.80 | 0.79 | 0.73 | 0.19 | 0.43 | 0.69 | 0.16 | 0.72 | 0.66 | 0.62 | 0.12 | 0.56 | 0.40 | 0.10 | 0.56 |
| ANCHOR BAY, CA | 0.05 | 0.17 | 0.34 | 0.03 | 0.49 | 0.34 | 0.74 | 0.61 | 0.37 | 0.91 | 0.21 | 0.72 | 0.78 | 0.53 | 0.76 | 0.55 |
| POINT ARENA, CA | 0.05 | 0.17 | 0.34 | 0.03 | 0.49 | 0.34 | 0.74 | 0.61 | 0.37 | 0.91 | 0.21 | 0.72 | 0.78 | 0.53 | 0.76 | 0.55 |
| REDWOOD CITY, CA | 0.78 | 0.67 | 0.85 | 0.50 | 0.32 | 0.72 | 0.79 | 0.07 | 0.84 | 0.16 | 0.51 | 0.14 | 0.70 | 0.31 | 0.12 | 0.54 |
| SAN FRANCISCO, CA | 0.98 | 0.76 | 0.73 | 1.00 | 0.04 | 0.78 | 0.90 | 0.43 | 0.10 | 0.37 | 0.19 | 0.35 | 0.68 | 0.55 | 0.12 | 0.54 |
| TORRANCE, CA | 0.90 | 0.31 | 0.70 | 0.40 | 0.32 | 0.79 | 0.74 | 0.24 | 0.50 | 0.43 | 0.45 | 0.40 | 0.48 | 0.32 | 0.38 | 0.54 |
| NIPOMO, CA | 0.15 | 0.03 | 0.55 | 0.14 | 0.80 | 0.53 | 0.58 | 0.50 | 0.52 | 0.72 | 0.65 | 0.57 | 0.62 | 0.27 | 0.44 | 0.53 |
| YOUNTVILLE, CA | 0.24 | 0.96 | 0.15 | 0.86 | 1.00 | 0.38 | 0.65 | 0.83 | 0.03 | 0.99 | 0.04 | 0.30 | 0.67 | 0.44 | 0.24 | 0.53 |
| ATASCADERO, CA | 0.59 | 0.76 | 0.71 | 0.55 | 0.71 | 0.35 | 0.21 | 0.51 | 0.48 | 0.43 | 0.50 | 0.10 | 0.37 | 0.62 | 0.53 | 0.53 |
| CORONADO, CA | 0.85 | 0.97 | 0.44 | 0.39 | 0.45 | 0.50 | 0.40 | 0.34 | 0.27 | 0.56 | 0.47 | 0.64 | 0.38 | 0.33 | 0.29 | 0.52 |
| SANTEE, CA | 0.63 | 0.71 | 0.47 | 0.21 | 0.67 | 0.47 | 0.26 | 0.38 | 0.83 | 0.21 | 0.67 | 0.54 | 0.30 | 0.21 | 0.53 | 0.52 |
| ASTORIA, OR | 0.50 | 0.83 | 0.20 | 0.88 | 0.50 | 0.11 | 0.17 | 0.87 | 0.41 | 0.65 | 0.28 | 0.67 | 0.35 | 0.55 | 0.77 | 0.52 |
| ST. HELENA, CA | 0.35 | 0.92 | 0.38 | 0.23 | 0.59 | 0.55 | 0.84 | 0.53 | 0.19 | 0.79 | 0.28 | 0.16 | 0.72 | 0.35 | 0.18 | 0.51 |
| PINOLE, CA | 0.66 | 0.28 | 0.54 | 0.41 | 0.14 | 0.88 | 0.72 | 0.64 | 0.34 | 0.49 | 0.56 | 0.45 | 0.58 | 0.11 | 0.45 | 0.51 |
| CHINOOK, WA | 0.00 | 0.77 | 0.00 | 0.09 | 0.95 | 0.12 | 0.19 | 0.91 | 0.78 | 0.74 | 0.11 | 0.15 | 0.53 | 0.73 | 0.84 | 0.50 |
| BELLINGHAM, WA | 0.94 | 0.89 | 0.17 | 0.96 | 0.43 | 0.21 | 0.22 | 0.68 | 0.15 | 0.36 | 0.40 | 0.60 | 0.19 | 0.93 | 0.65 | 0.50 |
| EL PASO DE ROBLES (PASO ROBLES), CA | 0.23 | 0.14 | 0.50 | 0.12 | 0.53 | 0.52 | 0.70 | 0.47 | 0.65 | 0.52 | 0.64 | 0.09 | 0.74 | 0.50 | 0.54 | 0.50 |
| LOS OSOS, CA | 0.36 | 0.44 | 0.77 | 0.22 | 0.74 | 0.30 | 0.39 | 0.74 | 0.35 | 0.79 | 0.12 | 0.29 | 0.47 | 0.52 | 0.48 | 0.49 |
| CASPAR, CA | 0.00 | 0.37 | 0.32 | 0.00 | 0.78 | 0.27 | 0.62 | 0.73 | 0.30 | 0.89 | 0.76 | 0.00 | 0.43 | 0.70 | 0.59 | 0.48 |
| MENDOCINO, CA | 0.00 | 0.37 | 0.32 | 0.00 | 0.78 | 0.27 | 0.62 | 0.73 | 0.30 | 0.89 | 0.76 | 0.00 | 0.43 | 0.70 | 0.59 | 0.48 |
| BROOKINGS, OR | 0.36 | 0.66 | 0.02 | 0.31 | 0.70 | 0.09 | 0.04 | 0.86 | 0.14 | 0.93 | 0.48 | 0.83 | 0.31 | 0.49 | 0.81 | 0.48 |
| SOUTH SAN FRANCISCO, CA | 0.78 | 0.40 | 0.78 | 0.67 | 0.37 | 0.89 | 0.77 | 0.27 | 0.24 | 0.40 | 0.35 | 0.07 | 0.69 | 0.18 | 0.42 | 0.48 |
| SOLVANG, CA | 0.43 | 0.48 | 0.27 | 0.05 | 0.55 | 0.48 | 0.55 | 0.18 | 0.51 | 0.66 | 0.87 | 0.45 | 0.17 | 0.65 | 0.36 | 0.47 |
| IRVINE, CA | 0.90 | 0.81 | 0.60 | 0.47 | 0.27 | 0.73 | 0.68 | 0.03 | 0.53 | 0.29 | 0.38 | 0.41 | 0.12 | 0.59 | 0.26 | 0.47 |
| EMERYVILLE, CA | 1.00 | 0.36 | 0.67 | 0.98 | 0.03 | 0.76 | 0.50 | 0.60 | 0.09 | 0.07 | 0.52 | 0.60 | 0.33 | 0.78 | 0.26 | 0.47 |
| SAN LUIS OBISPO, CA | 0.74 | 0.98 | 0.45 | 0.75 | 0.76 | 0.41 | 0.37 | 0.26 | 0.09 | 0.29 | 0.16 | 0.22 | 0.27 | 0.99 | 0.58 | 0.46 |
| GOLD BEACH, OR | 0.33 | 0.37 | 0.18 | 0.84 | 0.98 | 0.02 | 0.03 | 0.90 | 0.48 | 0.83 | 0.21 | 0.55 | 0.54 | 0.48 | 0.67 | 0.46 |
| LITTLE RIVER, CA | 0.18 | 0.88 | 0.17 | 0.04 | 0.77 | 0.17 | 0.41 | 0.81 | 0.07 | 0.89 | 0.45 | 0.27 | 0.17 | 0.81 | 0.50 | 0.45 |
| ALBANY, CA | 0.79 | 0.31 | 0.65 | 0.81 | 0.08 | 0.72 | 0.61 | 0.15 | 0.63 | 0.32 | 0.63 | 0.62 | 0.28 | 0.47 | 0.28 | 0.45 |
| MORRO BAY, CA | 0.24 | 0.68 | 0.48 | 0.62 | 0.83 | 0.22 | 0.25 | 0.74 | 0.11 | 0.90 | 0.15 | 0.60 | 0.35 | 0.51 | 0.44 | 0.45 |
| CAYUCOS, CA | 0.32 | 0.22 | 0.64 | 0.52 | 0.73 | 0.18 | 0.33 | 0.81 | 0.12 | 0.86 | 0.18 | 0.65 | 0.42 | 0.56 | 0.47 | 0.44 |
| PETALUMA, CA | 0.47 | 0.51 | 0.43 | 0.35 | 0.58 | 0.41 | 0.64 | 0.29 | 0.59 | 0.46 | 0.47 | 0.25 | 0.57 | 0.39 | 0.40 | 0.44 |
| ARROYO GRANDE, CA | 0.31 | 0.20 | 0.40 | 0.29 | 0.86 | 0.40 | 0.43 | 0.67 | 0.43 | 0.78 | 0.53 | 0.23 | 0.44 | 0.35 | 0.41 | 0.43 |
| BRENTWOOD, CA | 0.25 | 0.10 | 0.34 | 0.26 | 0.25 | 0.71 | 0.50 | 0.52 | 0.94 | 0.24 | 0.77 | 0.69 | 0.45 | 0.21 | 0.48 | 0.43 |
| CAMBRIA, CA | 0.23 | 0.26 | 0.72 | 0.26 | 0.69 | 0.33 | 0.49 | 0.75 | 0.06 | 0.97 | 0.02 | 0.59 | 0.48 | 0.40 | 0.46 | 0.43 |
| GARIBALDI, OR | 0.15 | 0.20 | 0.10 | 0.43 | 0.95 | 0.02 | 0.05 | 0.95 | 0.16 | 0.85 | 0.42 | 0.71 | 0.40 | 0.65 | 0.82 | 0.42 |
| PORT ORFORD, OR | 0.09 | 0.12 | 0.00 | 0.97 | 0.81 | 0.00 | 0.00 | 0.98 | 0.02 | 0.93 | 0.21 | 0.97 | 0.46 | 0.98 | 0.86 | 0.42 |
| ALBION, CA | 0.34 | 0.94 | 0.06 | 0.12 | 0.76 | 0.10 | 0.18 | 0.87 | 0.01 | 0.87 | 0.06 | 0.85 | 0.05 | 0.88 | 0.43 | 0.41 |
| HEALDSBURG, CA | 0.18 | 0.68 | 0.55 | 0.08 | 0.28 | 0.46 | 0.79 | 0.67 | 0.23 | 0.78 | 0.32 | 0.36 | 0.61 | 0.25 | 0.23 | 0.41 |
| VALLEY CENTER, CA | 0.17 | 0.16 | 0.43 | 0.07 | 0.89 | 0.51 | 0.52 | 0.41 | 0.62 | 0.64 | 0.43 | 0.26 | 0.53 | 0.33 | 0.50 | 0.41 |
| CAMARILLO, CA | 0.56 | 0.52 | 0.28 | 0.46 | 0.33 | 0.61 | 0.57 | 0.56 | 0.67 | 0.64 | 0.31 | 0.39 | 0.43 | 0.17 | 0.35 | 0.40 |
| FIELDBROOK, CA | 0.11 | 0.02 | 0.28 | 0.16 | 0.88 | 0.12 | 0.02 | 0.66 | 0.61 | 0.47 | 0.53 | 0.80 | 0.30 | 0.84 | 0.74 | 0.40 |
| ORICK, CA | 0.00 | 0.17 | 0.80 | 0.57 | 0.89 | 0.16 | 0.04 | 0.81 | 0.17 | 0.80 | 0.79 | 0.24 | 0.13 | 0.76 | 0.55 | 0.39 |
| TRINIDAD, CA | 0.00 | 0.17 | 0.80 | 0.57 | 0.89 | 0.16 | 0.04 | 0.81 | 0.17 | 0.80 | 0.79 | 0.24 | 0.13 | 0.76 | 0.55 | 0.39 |
| GOLETA, CA | 0.68 | 0.93 | 0.57 | 0.39 | 0.40 | 0.58 | 0.44 | 0.13 | 0.25 | 0.39 | 0.20 | 0.63 | 0.44 | 0.21 | 0.45 | 0.39 |
| BLAINE, WA | 0.28 | 0.17 | 0.12 | 0.33 | 0.90 | 0.08 | 0.12 | 0.80 | 0.36 | 0.80 | 0.38 | 0.99 | 0.36 | 0.41 | 0.51 | 0.38 |
| EL CERRITO, CA | 0.46 | 0.16 | 0.51 | 0.52 | 0.23 | 0.74 | 0.68 | 0.29 | 0.51 | 0.42 | 0.57 | 0.53 | 0.47 | 0.36 | 0.32 | 0.38 |
| SEQUIM, WA | 0.45 | 0.56 | 0.07 | 0.83 | 0.87 | 0.03 | 0.09 | 0.96 | 0.02 | 0.99 | 0.17 | 0.63 | 0.15 | 0.64 | 0.61 | 0.38 |
| TIBURON, CA | 0.75 | 0.95 | 0.19 | 0.91 | 0.22 | 0.40 | 0.24 | 0.37 | 0.55 | 0.69 | 0.64 | 0.57 | 0.07 | 0.57 | 0.02 | 0.37 |
| BURLINGAME, CA | 0.95 | 0.53 | 0.76 | 0.53 | 0.02 | 0.71 | 0.76 | 0.09 | 0.78 | 0.34 | 0.28 | 0.34 | 0.42 | 0.14 | 0.09 | 0.37 |
| RODEO, CA | 0.34 | 0.08 | 0.66 | 0.31 | 0.06 | 0.86 | 0.55 | 0.48 | 0.54 | 0.25 | 0.67 | 0.50 | 0.52 | 0.26 | 0.39 | 0.36 |
| PACIFIC CITY, OR | 0.09 | 0.03 | 0.62 | 0.24 | 0.86 | 0.10 | 0.13 | 0.98 | 0.20 | 0.85 | 0.13 | 0.06 | 0.45 | 0.72 | 0.90 | 0.36 |
| HUNTINGTON BEACH, CA | 0.69 | 0.22 | 0.46 | 0.24 | 0.45 | 0.60 | 0.59 | 0.32 | 0.42 | 0.55 | 0.52 | 0.27 | 0.38 | 0.31 | 0.34 | 0.36 |
| TIMBER COVE, CA | 0.10 | 0.87 | 0.22 | 0.10 | 0.31 | 0.14 | 0.47 | 0.86 | 0.01 | 1.00 | 0.02 | 0.75 | 0.29 | 0.77 | 0.31 | 0.35 |
| PALO ALTO, CA | 0.92 | 0.91 | 0.54 | 0.77 | 0.42 | 0.64 | 0.53 | 0.07 | 0.69 | 0.51 | 0.29 | 0.13 | 0.16 | 0.26 | 0.03 | 0.35 |
| FOSTER CITY, CA | 0.96 | 0.43 | 0.68 | 0.26 | 0.51 | 0.80 | 0.74 | 0.05 | 0.69 | 0.32 | 0.18 | 0.21 | 0.27 | 0.10 | 0.13 | 0.34 |
| SUNNYVALE, CA | 0.95 | 0.19 | 0.81 | 0.54 | 0.64 | 0.83 | 0.75 | 0.02 | 0.78 | 0.05 | 0.14 | 0.26 | 0.33 | 0.12 | 0.16 | 0.34 |
| SAN BRUNO, CA | 0.82 | 0.42 | 0.75 | 0.53 | 0.12 | 0.81 | 0.71 | 0.19 | 0.38 | 0.40 | 0.26 | 0.16 | 0.57 | 0.07 | 0.32 | 0.34 |
| SAN MIGUEL (SAN LUIS OBISPO COUNTY), CA | 0.67 | 0.40 | 0.52 | 0.74 | 0.44 | 0.48 | 0.60 | 0.34 | 0.50 | 0.62 | 0.54 | 0.09 | 0.28 | 0.30 | 0.22 | 0.33 |
| GUERNEVILLE, CA | 0.07 | 0.35 | 0.44 | 0.45 | 0.48 | 0.22 | 0.31 | 0.82 | 0.08 | 0.82 | 0.24 | 0.81 | 0.34 | 0.64 | 0.36 | 0.33 |
| AVALON, CA | 0.77 | 0.99 | 0.94 | 1.00 | 0.15 | 0.65 | 0.22 | 0.00 | 0.19 | 0.08 | 0.73 | 0.00 | 0.39 | 0.43 | 0.43 | 0.33 |
| ALTOONA, WA | 0.12 | 0.49 | 0.13 | 0.01 | 0.90 | 0.06 | 0.06 | 0.96 | 0.21 | 0.94 | 0.09 | 0.47 | 0.50 | 0.27 | 0.74 | 0.31 |
| CATHLAMET, WA | 0.12 | 0.49 | 0.13 | 0.01 | 0.90 | 0.06 | 0.06 | 0.96 | 0.21 | 0.94 | 0.09 | 0.47 | 0.50 | 0.27 | 0.74 | 0.31 |
| PUGET ISLAND, WA | 0.12 | 0.49 | 0.13 | 0.01 | 0.90 | 0.06 | 0.06 | 0.96 | 0.21 | 0.94 | 0.09 | 0.47 | 0.50 | 0.27 | 0.74 | 0.31 |
| SKAMOKAWA VALLEY, WA | 0.12 | 0.49 | 0.13 | 0.01 | 0.90 | 0.06 | 0.06 | 0.96 | 0.21 | 0.94 | 0.09 | 0.47 | 0.50 | 0.27 | 0.74 | 0.31 |
| DEPOE BAY, OR | 0.28 | 0.12 | 0.15 | 0.55 | 0.88 | 0.03 | 0.06 | 0.94 | 0.00 | 0.98 | 0.04 | 0.98 | 0.40 | 0.47 | 0.61 | 0.31 |
| PACIFICA, CA | 0.70 | 0.49 | 0.74 | 0.36 | 0.21 | 0.78 | 0.62 | 0.23 | 0.37 | 0.45 | 0.31 | 0.23 | 0.41 | 0.07 | 0.33 | 0.30 |
| FREMONT, CA | 0.76 | 0.26 | 0.86 | 0.40 | 0.29 | 0.93 | 0.76 | 0.04 | 0.72 | 0.17 | 0.16 | 0.24 | 0.37 | 0.06 | 0.30 | 0.29 |
| KALAMA, WA | 0.12 | 0.00 | 0.18 | 0.16 | 0.96 | 0.01 | 0.10 | 0.90 | 0.47 | 0.62 | 0.65 | 0.94 | 0.19 | 0.31 | 0.49 | 0.29 |
| FERNDALE, CA | 0.07 | 0.00 | 0.07 | 0.66 | 0.42 | 0.26 | 0.16 | 0.85 | 0.56 | 0.76 | 0.79 | 0.33 | 0.24 | 0.79 | 0.64 | 0.29 |
| OJAI, CA | 0.24 | 0.71 | 0.31 | 0.47 | 0.62 | 0.26 | 0.40 | 0.71 | 0.20 | 0.83 | 0.07 | 0.34 | 0.41 | 0.48 | 0.33 | 0.29 |
| CAMPBELL, CA | 0.91 | 0.46 | 0.63 | 0.51 | 0.18 | 0.69 | 0.63 | 0.20 | 0.68 | 0.24 | 0.43 | 0.13 | 0.33 | 0.19 | 0.19 | 0.28 |
| MONTEREY, CA | 0.61 | 0.93 | 0.53 | 0.62 | 0.19 | 0.45 | 0.37 | 0.38 | 0.36 | 0.67 | 0.16 | 0.40 | 0.20 | 0.29 | 0.33 | 0.28 |
| PLEASANT HILL, CA | 0.73 | 0.29 | 0.40 | 0.62 | 0.41 | 0.59 | 0.58 | 0.37 | 0.42 | 0.45 | 0.36 | 0.50 | 0.26 | 0.20 | 0.25 | 0.28 |
| BETHEL ISLAND, CA | 0.00 | 0.00 | 0.06 | 0.78 | 0.97 | 0.46 | 0.33 | 0.88 | 0.49 | 0.30 | 0.07 | 0.64 | 0.78 | 0.23 | 0.59 | 0.27 |
| SAN MATEO, CA | 0.93 | 0.55 | 0.64 | 0.45 | 0.02 | 0.73 | 0.72 | 0.16 | 0.67 | 0.39 | 0.25 | 0.11 | 0.36 | 0.13 | 0.10 | 0.27 |
| WESTHAVEN-MOONSTONE, CA | 0.00 | 0.09 | 0.52 | 0.12 | 0.86 | 0.11 | 0.03 | 0.69 | 0.33 | 0.66 | 0.73 | 0.38 | 0.16 | 0.59 | 0.60 | 0.26 |
| MARTINEZ, CA | 0.63 | 0.53 | 0.29 | 0.49 | 0.09 | 0.55 | 0.32 | 0.57 | 0.43 | 0.36 | 0.55 | 0.52 | 0.26 | 0.30 | 0.31 | 0.26 |
| POWAY, CA | 0.49 | 0.36 | 0.24 | 0.23 | 0.31 | 0.52 | 0.48 | 0.36 | 0.77 | 0.65 | 0.44 | 0.47 | 0.24 | 0.09 | 0.28 | 0.26 |
| NEWARK, CA | 0.60 | 0.14 | 0.90 | 0.17 | 0.04 | 0.90 | 0.73 | 0.05 | 0.85 | 0.09 | 0.23 | 0.18 | 0.52 | 0.04 | 0.40 | 0.25 |
| OCCIDENTAL, CA | 0.08 | 0.62 | 0.34 | 0.13 | 0.26 | 0.16 | 0.33 | 0.76 | 0.09 | 0.86 | 0.17 | 0.64 | 0.41 | 0.69 | 0.27 | 0.25 |
| BERKELEY, CA | 0.91 | 0.91 | 0.42 | 0.98 | 0.07 | 0.63 | 0.31 | 0.22 | 0.14 | 0.35 | 0.25 | 0.39 | 0.08 | 0.76 | 0.20 | 0.24 |
| CARLSBAD, CA | 0.74 | 0.15 | 0.29 | 0.36 | 0.61 | 0.38 | 0.36 | 0.30 | 0.67 | 0.63 | 0.51 | 0.31 | 0.17 | 0.25 | 0.27 | 0.24 |
| SEATTLE, WA | 0.99 | 0.79 | 0.38 | 0.98 | 0.09 | 0.52 | 0.53 | 0.28 | 0.19 | 0.16 | 0.33 | 0.25 | 0.25 | 0.61 | 0.24 | 0.24 |
| SAN CARLOS, CA | 0.89 | 0.74 | 0.49 | 0.56 | 0.48 | 0.59 | 0.45 | 0.08 | 0.76 | 0.33 | 0.24 | 0.21 | 0.15 | 0.10 | 0.08 | 0.23 |
| SIMI VALLEY, CA | 0.45 | 0.22 | 0.28 | 0.20 | 0.27 | 0.59 | 0.53 | 0.39 | 0.71 | 0.28 | 0.49 | 0.43 | 0.40 | 0.17 | 0.37 | 0.23 |
| SAN JUAN CAPISTRANO, CA | 0.47 | 0.23 | 0.45 | 0.42 | 0.52 | 0.47 | 0.47 | 0.22 | 0.73 | 0.59 | 0.31 | 0.28 | 0.45 | 0.16 | 0.23 | 0.22 |
| LA CONNER, WA | 0.32 | 0.05 | 0.08 | 0.65 | 0.56 | 0.25 | 0.15 | 0.78 | 0.29 | 0.88 | 0.43 | 0.22 | 0.49 | 0.48 | 0.55 | 0.22 |
| MOUNTAIN VIEW CITY, CA | 0.98 | 0.14 | 0.64 | 0.64 | 0.51 | 0.72 | 0.64 | 0.04 | 0.72 | 0.06 | 0.39 | 0.21 | 0.20 | 0.16 | 0.09 | 0.21 |
| SAUSALITO, CA | 0.81 | 0.82 | 0.24 | 0.37 | 0.05 | 0.33 | 0.60 | 0.50 | 0.25 | 0.71 | 0.50 | 0.17 | 0.08 | 0.39 | 0.05 | 0.21 |
| PESCADERO, CA | 0.06 | 0.89 | 0.27 | 0.05 | 0.69 | 0.23 | 0.71 | 0.59 | 0.10 | 0.67 | 0.00 | 0.05 | 0.55 | 0.53 | 0.14 | 0.21 |
| SONOMA, CA | 0.28 | 0.52 | 0.16 | 0.69 | 0.64 | 0.29 | 0.35 | 0.68 | 0.16 | 0.86 | 0.34 | 0.17 | 0.50 | 0.36 | 0.19 | 0.21 |
| WASHOUGAL, WA | 0.29 | 0.10 | 0.31 | 0.43 | 0.75 | 0.06 | 0.12 | 0.62 | 0.92 | 0.30 | 0.61 | 0.19 | 0.34 | 0.29 | 0.54 | 0.20 |
| SAN CLEMENTE, CA | 0.51 | 0.86 | 0.40 | 0.29 | 0.43 | 0.36 | 0.41 | 0.17 | 0.39 | 0.60 | 0.26 | 0.38 | 0.23 | 0.16 | 0.25 | 0.20 |
| NOVATO, CA | 0.57 | 0.56 | 0.47 | 0.25 | 0.36 | 0.42 | 0.42 | 0.31 | 0.40 | 0.71 | 0.32 | 0.17 | 0.26 | 0.20 | 0.22 | 0.19 |
| PISMO BEACH, CA | 0.39 | 0.17 | 0.29 | 0.42 | 0.65 | 0.36 | 0.28 | 0.62 | 0.35 | 0.75 | 0.54 | 0.18 | 0.20 | 0.27 | 0.31 | 0.19 |
| RANCHO PALOS VERDES, CA | 0.71 | 0.30 | 0.37 | 0.17 | 0.16 | 0.68 | 0.47 | 0.29 | 0.56 | 0.76 | 0.19 | 0.47 | 0.11 | 0.12 | 0.11 | 0.19 |
| SANTA MONICA, CA | 1.00 | 0.53 | 0.39 | 0.90 | 0.03 | 0.51 | 0.48 | 0.19 | 0.14 | 0.41 | 0.26 | 0.54 | 0.22 | 0.46 | 0.08 | 0.18 |
| HALF MOON BAY, CA | 0.30 | 0.11 | 0.67 | 0.07 | 0.60 | 0.55 | 0.79 | 0.11 | 0.60 | 0.42 | 0.12 | 0.12 | 0.66 | 0.03 | 0.14 | 0.18 |
| ANACORTES, WA | 0.40 | 0.26 | 0.09 | 0.71 | 0.40 | 0.09 | 0.10 | 0.79 | 0.15 | 0.91 | 0.46 | 0.36 | 0.18 | 0.52 | 0.46 | 0.17 |
| MISSION VIEJO, CA | 0.53 | 0.48 | 0.24 | 0.14 | 0.21 | 0.53 | 0.52 | 0.22 | 0.54 | 0.55 | 0.34 | 0.30 | 0.28 | 0.15 | 0.28 | 0.17 |
| REDONDO BEACH, CA | 0.84 | 0.07 | 0.53 | 0.21 | 0.16 | 0.67 | 0.50 | 0.10 | 0.57 | 0.26 | 0.39 | 0.35 | 0.34 | 0.12 | 0.22 | 0.17 |
| BENICIA, CA | 0.38 | 0.10 | 0.26 | 0.25 | 0.15 | 0.68 | 0.30 | 0.51 | 0.43 | 0.52 | 0.50 | 0.59 | 0.11 | 0.19 | 0.35 | 0.16 |
| THOUSAND OAKS, CA | 0.49 | 0.59 | 0.17 | 0.19 | 0.30 | 0.44 | 0.45 | 0.31 | 0.64 | 0.61 | 0.14 | 0.40 | 0.22 | 0.08 | 0.21 | 0.16 |
| NEWPORT BEACH, CA | 0.93 | 0.65 | 0.38 | 0.35 | 0.28 | 0.37 | 0.41 | 0.10 | 0.22 | 0.68 | 0.21 | 0.19 | 0.14 | 0.42 | 0.07 | 0.16 |
| LOS ALTOS, CA | 0.90 | 0.39 | 0.43 | 0.50 | 0.17 | 0.69 | 0.57 | 0.01 | 0.91 | 0.54 | 0.15 | 0.10 | 0.07 | 0.10 | 0.01 | 0.15 |
| LOS ALAMITOS, CA | 0.42 | 0.20 | 0.21 | 0.15 | 0.24 | 0.60 | 0.35 | 0.27 | 0.60 | 0.56 | 0.45 | 0.44 | 0.24 | 0.09 | 0.36 | 0.15 |
| MONTARA, CA | 0.30 | 0.24 | 0.63 | 0.30 | 0.41 | 0.54 | 0.50 | 0.32 | 0.70 | 0.50 | 0.27 | 0.08 | 0.32 | 0.00 | 0.21 | 0.14 |
| SUMMERLAND, CA | 0.46 | 0.34 | 0.03 | 0.18 | 0.52 | 0.13 | 0.22 | 0.45 | 0.29 | 0.97 | 0.36 | 0.84 | 0.14 | 0.26 | 0.00 | 0.14 |
| KENTFIELD, CA | 0.66 | 0.79 | 0.26 | 0.34 | 0.12 | 0.19 | 0.43 | 0.24 | 0.65 | 0.71 | 0.41 | 0.05 | 0.09 | 0.33 | 0.02 | 0.14 |
| NEHALEM, OR | 0.10 | 0.60 | 0.25 | 0.85 | 0.76 | 0.00 | 0.00 | 0.89 | 0.12 | 0.97 | 0.19 | 0.02 | 0.31 | 0.22 | 0.52 | 0.13 |
| JENNER, CA | 0.00 | 0.90 | 0.18 | 0.09 | 0.36 | 0.21 | 0.16 | 0.60 | 0.04 | 0.80 | 0.03 | 0.72 | 0.12 | 0.51 | 0.29 | 0.13 |
| MALIBU, CA | 0.29 | 0.98 | 0.11 | 0.12 | 0.60 | 0.29 | 0.06 | 0.44 | 0.11 | 0.72 | 0.36 | 0.57 | 0.05 | 0.22 | 0.00 | 0.12 |
| LA CANADA FLINTRIDGE, CA | 0.44 | 0.06 | 0.20 | 0.04 | 0.17 | 0.66 | 0.67 | 0.09 | 0.71 | 0.60 | 0.41 | 0.14 | 0.25 | 0.13 | 0.16 | 0.12 |
| DILLON BEACH, CA | 0.21 | 0.47 | 0.61 | 0.59 | 0.62 | 0.31 | 0.56 | 0.05 | 0.26 | 0.87 | 0.04 | 0.02 | 0.20 | 0.23 | 0.18 | 0.11 |
| NICASIO, CA | 0.21 | 0.47 | 0.61 | 0.59 | 0.62 | 0.31 | 0.56 | 0.05 | 0.26 | 0.87 | 0.04 | 0.02 | 0.20 | 0.23 | 0.18 | 0.11 |
| TOMALES, CA | 0.21 | 0.47 | 0.61 | 0.59 | 0.62 | 0.31 | 0.56 | 0.05 | 0.26 | 0.87 | 0.04 | 0.02 | 0.20 | 0.23 | 0.18 | 0.11 |
| WALNUT CREEK, CA | 0.79 | 0.69 | 0.14 | 0.63 | 0.05 | 0.40 | 0.38 | 0.48 | 0.36 | 0.82 | 0.11 | 0.20 | 0.02 | 0.06 | 0.10 | 0.10 |
| CAMAS, WA | 0.22 | 0.08 | 0.12 | 0.14 | 0.55 | 0.24 | 0.24 | 0.33 | 0.95 | 0.14 | 0.60 | 0.26 | 0.18 | 0.11 | 0.45 | 0.10 |
| DEL MAR, CA | 0.97 | 0.16 | 0.23 | 0.16 | 0.00 | 0.39 | 0.25 | 0.00 | 0.13 | 0.64 | 0.28 | 0.51 | 0.07 | 0.75 | 0.07 | 0.10 |
| PLEASANTON, CA | 0.72 | 0.12 | 0.33 | 0.20 | 0.20 | 0.70 | 0.46 | 0.02 | 0.92 | 0.14 | 0.42 | 0.07 | 0.10 | 0.03 | 0.16 | 0.09 |
| LIVERMORE, CA | 0.48 | 0.13 | 0.34 | 0.28 | 0.21 | 0.60 | 0.44 | 0.10 | 0.87 | 0.17 | 0.38 | 0.10 | 0.29 | 0.02 | 0.24 | 0.09 |
| BODEGA BAY, CA | 0.14 | 0.72 | 0.22 | 0.21 | 0.40 | 0.17 | 0.27 | 0.49 | 0.05 | 0.92 | 0.03 | 0.16 | 0.14 | 0.47 | 0.17 | 0.09 |
| MUIR BEACH, CA | 0.38 | 0.98 | 0.11 | 0.01 | 0.00 | 0.01 | 0.69 | 0.80 | 0.29 | 0.62 | 0.01 | 0.04 | 0.00 | 0.32 | 0.09 | 0.08 |
| SEAL BEACH, CA | 0.65 | 0.21 | 0.16 | 0.48 | 0.20 | 0.42 | 0.28 | 0.58 | 0.12 | 0.84 | 0.10 | 0.32 | 0.12 | 0.14 | 0.21 | 0.08 |
| MOSS BEACH, CA | 0.16 | 0.45 | 0.56 | 0.40 | 0.66 | 0.47 | 0.28 | 0.12 | 0.84 | 0.34 | 0.20 | 0.04 | 0.06 | 0.01 | 0.14 | 0.07 |
| LOS GATOS, CA | 0.54 | 0.40 | 0.21 | 0.11 | 0.18 | 0.50 | 0.32 | 0.14 | 0.86 | 0.44 | 0.21 | 0.31 | 0.09 | 0.02 | 0.07 | 0.07 |
| ENCINITAS, CA | 0.55 | 0.23 | 0.26 | 0.29 | 0.26 | 0.23 | 0.31 | 0.08 | 0.58 | 0.58 | 0.48 | 0.29 | 0.10 | 0.14 | 0.15 | 0.07 |
| DANA POINT, CA | 0.47 | 0.27 | 0.16 | 0.32 | 0.34 | 0.32 | 0.39 | 0.33 | 0.28 | 0.74 | 0.13 | 0.28 | 0.16 | 0.17 | 0.13 | 0.06 |
| CORTE MADERA, CA | 0.81 | 0.45 | 0.04 | 0.74 | 0.17 | 0.20 | 0.14 | 0.14 | 0.79 | 0.70 | 0.46 | 0.15 | 0.03 | 0.05 | 0.03 | 0.06 |
| INVERNESS, CA | 0.14 | 0.54 | 0.30 | 0.43 | 0.39 | 0.25 | 0.36 | 0.16 | 0.07 | 0.96 | 0.03 | 0.02 | 0.47 | 0.22 | 0.20 | 0.05 |
| RIDGEFIELD, WA | 0.00 | 0.03 | 0.09 | 0.18 | 0.72 | 0.09 | 0.25 | 0.20 | 0.94 | 0.41 | 0.30 | 0.37 | 0.09 | 0.15 | 0.41 | 0.05 |
| LAFAYETTE, CA | 0.58 | 0.83 | 0.03 | 0.52 | 0.03 | 0.30 | 0.21 | 0.46 | 0.40 | 0.92 | 0.08 | 0.14 | 0.02 | 0.01 | 0.05 | 0.05 |
| MILL VALLEY, CA | 0.62 | 0.70 | 0.05 | 0.55 | 0.13 | 0.14 | 0.20 | 0.21 | 0.74 | 0.69 | 0.33 | 0.08 | 0.01 | 0.05 | 0.01 | 0.04 |
| STINSON BEACH, CA | 0.21 | 0.95 | 0.09 | 0.01 | 0.00 | 0.03 | 0.51 | 0.46 | 0.03 | 0.94 | 0.01 | 0.03 | 0.00 | 0.50 | 0.12 | 0.04 |
| TOPANGA, CA | 0.26 | 0.06 | 0.02 | 0.05 | 0.33 | 0.19 | 0.36 | 0.25 | 0.44 | 0.77 | 0.44 | 0.56 | 0.05 | 0.07 | 0.04 | 0.03 |
| WESTLAKE VILLAGE, CA | 0.20 | 0.44 | 0.01 | 0.05 | 0.34 | 0.28 | 0.19 | 0.28 | 0.55 | 0.79 | 0.10 | 0.20 | 0.06 | 0.04 | 0.05 | 0.03 |
| EL SEGUNDO, CA | 0.55 | 0.03 | 0.18 | 0.24 | 0.10 | 0.37 | 0.20 | 0.03 | 0.32 | 0.14 | 0.34 | 0.69 | 0.10 | 0.19 | 0.11 | 0.03 |
| AVILA BEACH, CA | 0.31 | 0.04 | 0.03 | 0.33 | 0.81 | 0.14 | 0.02 | 0.53 | 0.07 | 0.90 | 0.07 | 0.05 | 0.04 | 0.09 | 0.15 | 0.02 |
| DANVILLE, CA | 0.39 | 0.21 | 0.05 | 0.36 | 0.01 | 0.45 | 0.21 | 0.05 | 0.95 | 0.52 | 0.17 | 0.11 | 0.03 | 0.02 | 0.06 | 0.02 |
| ALAMO, CA | 0.40 | 0.32 | 0.02 | 0.60 | 0.02 | 0.17 | 0.18 | 0.67 | 0.28 | 0.98 | 0.08 | 0.06 | 0.01 | 0.00 | 0.04 | 0.02 |
| SOLANA BEACH, CA | 0.75 | 0.00 | 0.10 | 0.11 | 0.08 | 0.22 | 0.28 | 0.02 | 0.28 | 0.78 | 0.37 | 0.12 | 0.03 | 0.05 | 0.06 | 0.01 |
| BOLINAS, CA | 0.00 | 0.67 | 0.04 | 0.08 | 0.06 | 0.13 | 0.16 | 0.11 | 0.00 | 1.00 | 0.02 | 0.01 | 0.23 | 0.44 | 0.19 | 0.01 |
| HERMOSA BEACH, CA | 0.71 | 0.05 | 0.12 | 0.15 | 0.11 | 0.39 | 0.17 | 0.03 | 0.44 | 0.31 | 0.12 | 0.19 | 0.04 | 0.08 | 0.03 | 0.00 |
| MANHATTAN BEACH, CA | 0.44 | 0.02 | 0.08 | 0.09 | 0.13 | 0.33 | 0.14 | 0.01 | 0.45 | 0.28 | 0.14 | 0.33 | 0.02 | 0.03 | 0.02 | 0.00 |
