# Supplementary material for: Social-ecological vulnerability of fishing communities to climate change: A U.S. West Coast case study
Source: PLoS One. 2022 Aug 17;17(8):e0272120. doi: 10.1371/journal.pone.0272120 (PMC9385011; doi:10.1371/journal.pone.0272120)
Supplement: S7 Table — Fishing communities removed from analysis because of confidential landings data. Adaptive capacity, based on social indicators, is calculated so that smaller values (closer to 0) equal greater adaptive capacity and greater values (close to 1) equal lower adaptive capacity. Sensitivity is community economic reliance on commercial fishing. Values are from percentile ranks across all communities (included and removed). (DOCX) [file pone.0272120.s012.docx]

| Community | Adaptive Capacity Percentile rank | Sensitivity Percentile rank |
| --- | --- | --- |
| APTOS, CA | 0.093851133 | 0 |
| CAPITOLA, CA | 0.323624595 | 0 |
| CARMEL-BY-THE-SEA, CA | 0.058252427 | 0.613636364 |
| DAVENPORT, CA | 0.359223301 | 0 |
| FELTON, CA | 0.097087379 | 0 |
| FREEDOM, CA | 0.928802589 | 0 |
| GILROY, CA | 0.737864078 | 0 |
| LONG BEACH, CA | 0.919093851 | 0.613636364 |
| MARINA, CA | 0.886731392 | 0 |
| MORGAN HILL, CA | 0.475728155 | 0 |
| OCEANSIDE, CA | 0.750809061 | 0.724025974 |
| PACIFIC GROVE, CA | 0.190938511 | 0 |
| POINT REYES STATION, CA | 0.042071197 | 0.844155844 |
| SALINAS, CA | 0.922330097 | 0 |
| SEASIDE, CA | 0.854368932 | 0 |
| SOQUEL, CA | 0.414239482 | 0 |
| WATSONVILLE, CA | 0.996763754 | 0 |
| WILLOW CREEK, CA | 0.585760518 | 0 |
| CANNON BEACH, OR | 0.443365696 | 0 |
| GEARHART, OR | 0.601941748 | 0.863636364 |
| NETARTS, OR | 0.25566343 | 0.909090909 |
| SEASIDE, OR | 0.550161812 | 0.792207792 |
| SILETZ, OR | 0.699029126 | 0.788961039 |
| WALDPORT, OR | 0.265372168 | 0.850649351 |
| YACHATS, OR | 0.469255663 | 0 |
| ABERDEEN, WA | 0.83171521 | 0.74025974 |
| BAY CENTER, WA | 0.844660194 | 0.938311688 |
| BREMERTON, WA | 0.627831715 | 0.662337662 |
| BRINNON, WA | 0.605177994 | 0 |
| COPALIS BEACH, WA | 0.482200647 | 0 |
| COUPEVILLE, WA | 0.155339806 | 0.775974026 |
| FRIDAY HARBOR, WA | 0.194174757 | 0.827922078 |
| GRAYLAND, WA | 0.825242718 | 0 |
| HOQUIAM, WA | 0.663430421 | 0 |
| LONG BEACH, WA | 0.72815534 | 0.850649351 |
| NASELLE, WA | 0.488673139 | 0.896103896 |
| OAKVILLE, WA | 0.566343042 | 0 |
| OLYMPIA, WA | 0.556634304 | 0.762987013 |
| POINT ROBERTS, WA | 0.181229773 | 0 |
| PORT TOWNSEND, WA | 0.300970874 | 0.814935065 |
| POULSBO, WA | 0.13592233 | 0.785714286 |
| QUEETS, WA | 0.932038835 | 0 |
| QUILCENE, WA | 0.349514563 | 0.847402597 |
| RAYMOND, WA | 0.873786408 | 0.769480519 |
| SHELTON, WA | 0.912621359 | 0.834415584 |
| SOUTH BEND, WA | 0.873786408 | 0.792207792 |
| STANWOOD, WA | 0.177993528 | 0 |
| TAHOLAH, WA | 0.925566343 | 0.961038961 |
| TOKELAND, WA | 0.844660194 | 0.951298701 |
| WHIDBEY ISLAND STATION (AULT FIELD), WA | 0.527508091 | 0 |
