# Supplementary material for: Social-ecological vulnerability of fishing communities to climate change: A U.S. West Coast case study
Source: PLoS One. 2022 Aug 17;17(8):e0272120. doi: 10.1371/journal.pone.0272120 (PMC9385011; doi:10.1371/journal.pone.0272120)
Supplement: S1 Data — (DOCX) [file pone.0272120.s013.docx]

Data for the analysis presented in this manuscript came from a variety of sources.

Much of the relevant data are within the manuscript and its Supporting Information
files, including values calculated in this paper such as ecological sensitivity, exposure,
risk, and community exposure, sensitivity, adaptive capacity, and vulnerability.
Additionally, summarized data needed to calculate community exposure, sensitivity,
adaptive capacity, and vulnerability are provided along with R code at the time of
publication at <https://github.com/koehnl/CommunityVuln_PlosOne> (for code) or on Dryad at <https://doi.org/10.5061/dryad.547d7wm9d> (for data).

Certain data underlying the above values presented in the study are publicly available.
Specifically, through Aquamaps: https://www.aquamaps.org/ for species ranges to
determine ecological risk or by contacting Aquamaps at info.aquamaps@gmail.com.
Additional raster files needed to construct species range files are available here
through the github page cited in this paper: O'hara CC, Afflerbach JC, Scarborough C,
Kaschner K, Halpern BS. Aligning marine species range data to better serve science
and conservation. PLoS One. 2017 May 3;12(5):e0175739.
https://doi.org/10.1371/journal.pone.0175739 and at the associated git repository
https://github.com/OHI-Science/IUCN-AquaMaps (and as part of the code to rasterize
species range data available at the time of publication on github here:
https://github.com/koehnl/CommunityVuln_PlosOne). Social metric data for calculating adaptive capacity for communities are available through the CDC here:
<https://www.atsdr.cdc.gov/placeandhealth/svi/data_documentation_download.html>

The summarized tables of climate variables experienced by species in their ranges
(output from the climate models), needed to calculate ecological exposure, sensitivity,
and risk, will be available via Dryad (https://datadryad.org/stash) at the time of
acceptance and publication (here: <https://doi.org/10.5061/dryad.547d7wm9d>). The underlying physical and biogeochemical variables from the downscaled projections are available upon request from authors Mike Jacox at NOAA (michael.jacox@noaa.gov) or Jerome Fiechter at UC Santa Cruz ([fiechter@ucsc.edu](mailto:fiechter@ucsc.edu)).

Confidential vessel-level landings data may be acquired by direct request from the
Pacific Fisheries Information Network (PacFIN) (https://pacfin.psmfc.org/) or the

Departments of Fish and Wildlife in California, Oregon, and Washington, subject to a
non-disclosure agreement. Aggregated data used to determine top species landed for
each community and percent revenue from each species for each community, and all
associated R code is publicly available at
<https://github.com/koehnl/CommunityVuln_PlosOne> for R code and <https://doi.org/10.5061/dryad.547d7wm9d> for aggregated data (aggregated landings by ports can also be found here <https://reports.psmfc.org/pacfin/f?p=501:1000>:::::: and go to “All species by port group”.

Values for community reliance (from NOAA California Current Integrated Ecosystem
Assessment) are provided in the same Dryad repository. Also, data on PacFIN ports and species used are in the Dryad repository (here: <https://doi.org/10.5061/dryad.547d7wm9d>) formulated from https://pacfin.psmfc.org/pacfin_pub/codes.php.

All code used in analysis presented in this manuscript are available on github at
<https://github.com/koehnl/CommunityVuln_PlosOne>.
